# Use of Artificial Intelligence in Adolescents’ Mental Health Care: Systematic Scoping Review of Current Applications and Future Directions

**DOI:** 10.2196/70438

**Published:** 2025-06-06

**Authors:** Gauri Sharma, Mark J Yaffe, Pooria Ghadiri, Rushali Gandhi, Laura Pinkham, Genevieve Gore, Samira Abbasgholizadeh-Rahimi

**Affiliations:** 1 Department of Family Medicine, McGill University, 5858 Ch. de la Côte-des-Neiges, Montreal, QC, Canada, 1 5143987375; 2 Department of Electrical and Computer Engineering, McGill University, Montreal, QC, Canada; 3 Mila-Quebec AI Institute, Montreal, QC, Canada; 4 St. Mary’s Hospital Center, Montréal West Island Integrated University Health and Social Services Centre (CIUSSS ODIM), Montreal, QC, Canada; 5 Schulich Library of Physical Sciences, Life Sciences, and Engineering, McGill University, Montreal, QC, Canada; 6 Lady Davis Institute for Medical Research (LDI), Jewish General Hospital, Montreal, QC, Canada

**Keywords:** mental health, artificial intelligence, machine learning, adolescents, adolescents’ mental health

## Abstract

**Background:**

Given the increasing prevalence of mental health problems among adolescents, early intervention and appropriate management are needed to decrease mortality and morbidity. Artificial intelligence’s (AI) potential contributions, although significant in the field of medicine, have not been adequately studied in the context of adolescents’ mental health.

**Objective:**

This review aimed to identify AI interventions that have been tested, implemented, or both, for use in adolescents’ mental health care.

**Methods:**

We used the Arksey and O’Malley framework, further refined by Levac et al, along with the Joanna Briggs Institute methodology, to guide this scoping review. We searched 5 electronic databases from the inception date through July 2024 (inclusive). Four independent reviewers screened the titles and abstracts, read the full texts, and extracted data using a validated data extraction form. Disagreements were resolved by consensus, and if this was not possible, the opinion of a fifth reviewer was sought. We evaluated the risk of bias (ROB) for prognosis and diagnosis-related studies using the Prediction Model Risk of Bias Assessment Tool. We followed the PRISMA-ScR (Preferred Reporting Items for Systematic reviews and Meta-Analyses extension for Scoping Reviews) checklist for reporting.

**Results:**

Of the papers screened, 88 papers relevant to our eligibility criteria were identified. Among the included papers, AI was most commonly used for diagnosis (n=78), followed by monitoring and evaluation (n=19), treatment (n=10), and prognosis (n=6). As some studies addressed multiple applications, categories are not mutually exclusive. For diagnosis, studies primarily addressed suicidal behaviors (n=11) and autism spectrum disorder (n=7). Machine learning was the most frequently reported AI method across all application areas. The overall ROB for diagnostic and prognostic models was predominantly unclear (58%), while 20% of studies had a high ROB and 22% were assessed as low risk.

**Conclusions:**

In our review, we found that AI is being applied across various areas of adolescent mental health care, spanning diagnosis, treatment planning, symptom monitoring, and prognosis. Interestingly, most studies to date have concentrated heavily on diagnostic tools, leaving other important aspects of care relatively underexplored. This presents a key opportunity for future research to broaden the scope of AI applications beyond diagnosis. Moreover, future studies should emphasize the meaningful and active involvement of end users in the design, development, and validation of AI interventions, alongside improved transparency in reporting AI models, data handling, and analytical processes to build trust and support safe clinical implementation.

## Introduction

### Mental Health Among Adolescents

According to the World Health Organization, adolescence is defined as the age interval between 10 and 19 years [1]. It is a pivotal time of life when vulnerability to mental health issues increases [2] as it encompasses critical years for mental, social, and emotional growth [3]. It is a period when questioning of one’s identity is common, along with the gradual assertion of independence [4]. This may be accompanied by rebellion against parents, while self-expression within the world at large begins. During adolescence, there are important hormonal shifts, and the brain undergoes significant developmental changes in neural pathways and behavioral patterns that will remain throughout adulthood, impacting mental health [5]. Certain mental illnesses that begin in adolescence continue into adulthood, causing long-term morbidity and a significant burden on society [5,6].

Globally, it is estimated that while 1 in 7 adolescents experience mental health problems, these remain largely unrecognized and untreated [7]. A meta-analysis of 29 studies involving 80,879 youth during the COVID-19 pandemic revealed that depressive and anxiety symptoms among children and adolescents had doubled compared with prepandemic estimates, highlighting the impact of external stressors on this vulnerable population [8]. Reviews have further emphasized that this mental health burden is compounded by increased screen time and social media use, with complex bidirectional relationships identified between digital media exposure and psychological well-being [9,10]. Suicide is the fourth most significant cause of death among older adolescents (15‐19 y) [7], accounting for 9.1% of all mortalities in this age range [11]. In 2016, the worldwide incidence of excessive episodic drinking among adolescents aged 15 to 19 years was 13.6%, with males being the most vulnerable [12]. Mental health problems that begin early tend to continue throughout adulthood [13]. Therefore, there are serious consequences from such illnesses during adolescence and later in life [14]. This supports the need for early intervention and appropriate management of adolescents’ mental health problems [13,15].

### Role of Artificial Intelligence in Adolescents’ Mental Health Care

Artificial intelligence (AI) is a branch of computer science and engineering dedicated to developing systems that can perform tasks typically requiring human intelligence, such as learning, reasoning, and decision-making [16,17]. AI has been proven to have the ability to deliver significant benefits in a variety of sectors and levels of health care [18], including administrative planning [19], resource management [20], prevention [21], screening [22], diagnostics [23], and treatment [24,25]. Adolescents as a collective are deeply engaged with utilizing technology, particularly social media, and perceive it as a mainly positive experience [17]. However, recent reviews have revealed a nuanced relationship between technological engagement and mental health outcomes [9]. While social media can provide valuable social support and connection, excessive use has been associated with an increased risk of anxiety, depression, and other mental health challenges [26]. In light of this complex relationship, technology is increasingly seen as a possible avenue for developing targeted solutions to health problems [19].

Comprehensive reviews of AI-enabled mental health interventions have demonstrated promising results, particularly in the form of chatbots and digital health platforms that can provide immediate support and increased access to mental health resources [9]. These digital interventions have shown promise in delivering mental health care, offering new pathways for treatment and support, with reviews highlighting their potential to overcome barriers to traditional care access [10]. Furthermore, advanced machine learning (ML) approaches have demonstrated significant potential in predicting suicide risk through social media patterns, offering new possibilities for early intervention and prevention strategies [27].

At the patient-level, Voss et al [28] have demonstrated how a socialization intervention for autistic adolescents that combined wearable AI-assisted Google Glass helped them reflect on their moods and emotions, resulting in better socializing skills [28]. Similarly, McGinnis et al [29] developed a digital phenotype for adolescents’ internalizing psychopathology using wearable sensors, finding that tasks inducing positivity, anxiety, and fear could identify depressive, anxious, and trauma-related disorders with 75% accuracy [29]. Chang et al [30] assessed methylphenidate’s efficacy in attention deficit hyperactivity disorder (ADHD) treatment through neuroimaging markers, using a pattern recognition method to analyze volumetric differences in structural magnetic resonance imaging [30]. Their study employed support vector machine classification to distinguish between varying volumetric measures [30]. In a related study, Li et al [31] used ML techniques to examine gray matter volume correlations in adolescents with ADHD, successfully differentiating between ADHD and non-ADHD structural magnetic resonance imaging samples.

At the health care professionals’ (HCP) level, AI technologies are proving instrumental in supporting HCPs who work with underprivileged adolescents. These tools facilitate earlier intervention, enhance engagement with therapeutic services, and raise awareness of mental health conditions, such as through the deployment of therapeutic chatbots [32,33]. These technological interventions are particularly valuable given the findings of recent reviews demonstrating the effectiveness of digital mental health interventions in reaching underserved populations [9,10]. At the system level, AI has the potential to address data shortage, particularly in non-integrated health care settings, by aiding in data collection through patient tracking and surveillance during screening procedures [34]. The integration of AI-powered tools has shown promise in standardizing mental health assessments and improving the accuracy of early detection systems, particularly when combined with traditional clinical approaches [14]. Recognizing both the potential of AI and the existing gaps in the literature, we conducted a systematic scoping review to synthesize current evidence on AI applications in adolescent mental health. Our goal is to provide a foundational understanding that may inspire future research and innovation in this rapidly evolving field.

## Methods

### Study Design

We used the Arksey and O’Malley framework [35], further refined by Levac et al [36], along with the Joanna Briggs Institute methodology [37], to guide this scoping review. This framework includes the following steps: (1) defining the review topic, objective, and research questions; (2) identifying relevant publications; (3) choosing studies that matched our eligibility criteria; (4) extracting and charting data from included studies; (5) aggregating, summarizing, and reporting the findings; and (6) discussing the findings with relevant stakeholders including HCPs and AI researchers/experts.

The protocol of the study is registered and accessible on the Open Science Framework website [38] . We used the PRISMA-ScR (Preferred Reporting Items for Systematic Reviews and Meta-Analysis-Scoping Reviews) reporting guideline for reporting the study [39] (Checklist 1). We used the PROBAST (Prediction Model Risk of Bias Assessment Tool) to examine the risk of bias (ROB) in eligible studies. This tool evaluates the ROB and usability of diagnostic and prognostic predictions from studies that used AI models [40]. PROBAST is classified into 4 domains: participants, predictors, outcomes, and analysis, while ROB may be judged as low, high, or unclear [40]. By using PROBAST, we aimed to critically evaluate and provide insights into the potential biases associated with the relevant included studies.

### Eligibility Criteria

#### Overview

We used the Population, Intervention, Comparison, Outcomes, Setting, and Study (PICOS) design components to develop our search approach for peer-reviewed papers in English [41].

#### Population

We included studies addressing (1) individuals between 10 and 19 years of age and (2) HCPs who provide care to adolescents with mental health problems, such as nurses, social workers, dietitians, public health practitioners, family physicians, community-based workers, clinical psychologists, pediatricians, psychiatrists, and pediatric psychiatrists.

#### Intervention

This study includes any AI system, in accordance with McCarthy’s definition [42]. We included studies that “tested” or “implemented” or “tested and implemented” AI methods, such as computer heuristics, expert systems, fuzzy logic, knowledge representation, automated reasoning, data mining, and ML. We excluded studies related to robot-assisted care and studies using simulated data to replicate real-world situations [43].

#### Comparators/Control

There was no restriction under this design component.

#### Outcome

Our outcome of interest related to: (1) adolescents; (2) HCPs; and (3) health care system.

#### Setting and Study Design

We included studies in any health care setting, and all designs and publication types except reviews, opinion pieces, conference papers and abstracts, editorials, commentaries, news, and letters.

### Information Sources and Search Strategy

An experienced information specialist (GG) developed and executed comprehensive literature searches. The systematic literature search was conducted in an iterative manner across 5 electronic bibliographic databases: MEDLINE (Ovid), EMBASE (Ovid), Web of Science Core Collection, Compendex, and INSPEC. Initially, we included studies published until February 2020. Subsequently, given the rapid developments in the field of AI applications in adolescent mental health care, we extended our search through July 2024 (inclusive). This approach resulted in nearly tripling our included studies, ensuring our review reflected the current state of technological advancements in this domain. Retrieved records were managed with EndNote X9.3.3 (Clarivate) and imported into review software (ie, DistillerSR, Evidence Partners, Ottawa, ON) to facilitate the selection process (Multimedia Appendix 1).

### Selection Process

Four independent reviewers (PG, LP, GS, and RG) screened the titles and abstracts of the initially identified records. The same 4 reviewers then independently assessed the full texts of studies selected in Level 1 screening for eligibility. In cases where disagreements arose, the reviewers first discussed their differences to attempt a resolution through consensus. If a consensus could not be reached, a fifth reviewer (SAR) was consulted to make the final decision. Studies that met the eligibility criteria were then included for full data extraction.

### Data Collection

A data extraction form, informed by aspects of the Cochrane Effective Practice and Organization of Care (EPOC) review group data collection checklist [44], was created and completed with team members’ input. We extracted study characteristics (eg, country of research, aim of study), population characteristics (eg, adolescents’ gender, age, and race), intervention characteristics (eg, various AI methods used, performance measures of AI interventions), and outcome characteristics (eg, AI application on the continuum of adolescents’ mental health care).

### Assessment of Risk of Bias

Three reviewers independently (GS, RG, and PG) assessed the included studies using the PROBAST criteria to determine the ROB in each eligible study for PROBAST evaluation [40]. Fourth reviewer (SAR) validated the assessments.

### Synthesis

The techniques used to build preliminary synthesis comprised textual descriptions of the studies, grouping, clustering, and tabulation [45]. We used a narrative technique to synthesize the data after extracting it from the relevant studies. We particularly detailed the characteristics of AI interventions, the nature and conditions of adolescents’ mental health, and whether or not end users were engaged in the development or validation process of these interventions. For data synthesis, we used Distiller software and Microsoft Excel.

### Consultation

Throughout the review, we kept all study team members up to date and solicited their comments about the quality and accuracy of the ongoing data-gathering process. Furthermore, we had the opportunity to present our preliminary findings to a diverse group of participants (primary care researchers, AI-health care researcher, and family physician) at 2 virtual symposiums, FMF 2021 [46], and AAAI 2021 Spring Symposium Series on Applied AI in Healthcare: Safety, Community, and the Environment [47]. We carefully considered and integrated the received feedback into our review, enhancing its overall quality and robustness.

## Results

### Overview

**Figure 1.**
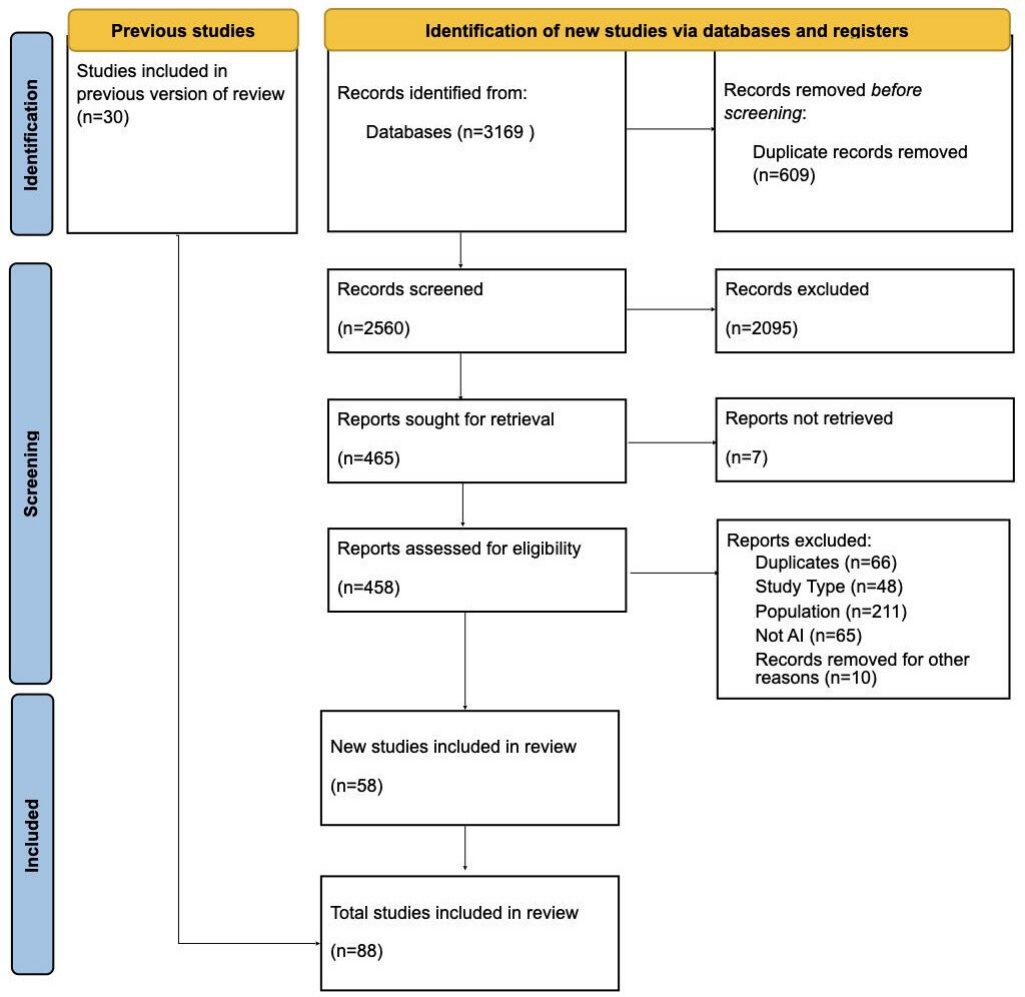
PRISMA (Preferred Reporting Items for Systematic Reviews and Meta-Analyses) flowchart of the selection procedure. AI: artificial intelligence.

Through searching the identified bibliographic databases, we obtained 4213 papers. After deduplication, we obtained 3364 papers. Finally, 88 papers were included after 2 rounds of screenings for data charting and analysis (Figure 1).

### Study Characteristics and Aims

#### Overview

The 88 papers included in this review aimed to test and implement an AI model in mental health care for adolescents.

#### Countries and Publication Dates

**Figure 2.**
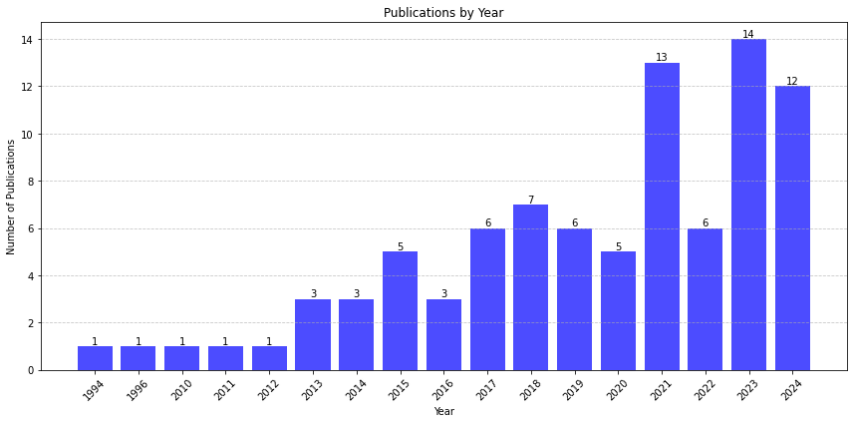
Frequency and timeline of artificial intelligence research studies by year of publication.

Considering the 88 included papers, despite a plateau trend from 1994 to 2012, the number of studies published annually on adolescents’ mental health using AI has progressively increased until 2018, when a decrease was observed. This trend continued with the start of the COVID-19 pandemic in early 2020. However, an increase in studies was observed after February 2020. Figure 2 depicts the timeline of AI-based papers (n=88). Four countries contributed the highest number of publications: the United States (n=26), China (n=18), the United Kingdom (n=5), and South Korea (n=5).

### Population Characteristics

#### Adolescents

Of the 88 papers, 68 stated their sample size. Among those 68 papers, 42 reported the sex distribution. The race/ethnicity of the participants was reported in 34 of the 88 papers (Table 1).

**Table 1.** Adolescents’ variables reported (N=88).

Reported variables	Values, n (%)
Sex	44 (50)
Sample size	68 (77)
Race/ethnicities^a^	34 (38.6)
Other sociodemographic^b^	50 (56.8)

a Race/ethnicities: Caucasian/White, Japanese, Chinese, Indian, Malay, Black, Oriental, African American, Asian-American, Hispanic, Native American/Alaskan, Asian, and Mixed.

b Other sociodemographic: parental marital status, parental educational level achieved, socioeconomic aspects (eg, parental and household income), place of residence, criminal records, maternal history of depression, neighborhood level of deprivation, adolescents’ traumatic events, and their level of education.

#### Health Care Professionals

No studies provided information regarding the total number, sex, gender, age, and race/ethnicity of the HCPs. Their only role was described as collaborators who assisted researchers in assigning eligible adolescents to a study or confirming the adolescents’ mental health disorders before using the AI systems. Adolescent psychiatrists, child psychiatrists, mental health professionals, pediatric-psychiatric clinicians, and adolescent forensic psychiatrists were among the HCPs involved in the mentioned process.

### Intervention

#### AI Methods

**Figure 3.**
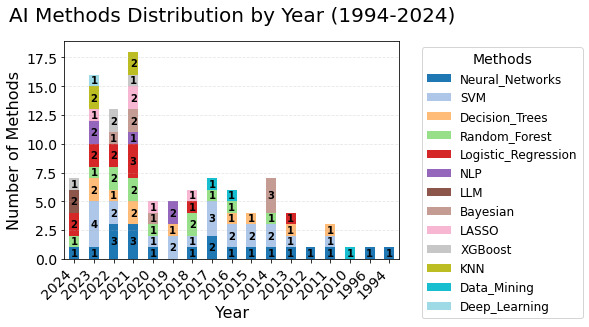
Distribution of AI methods in research studies over the years. AI: artificial intelligence; KNN: k-nearest neighbours; LLM: large language model; NLP: natural language processing; SVM: support vector machine.

Out of 88 included papers, 86 papers (97.7 %) reported on the testing and usage of off-the-shelf AI models. Such models were readily/instantly accessible or relevant to the specific context with slight modifications, but may have also been applicable to a wide range of other contexts or issues [48]. A total of 21 papers (23%) used support vector machine (Figure 3) as the most used AI method. Details of the AI methods are provided in the Multimedia Appendix 2.

#### Performance of AI systems

Out of 88 included papers, 11 papers (13%) did not report the model performance. Of all the methods used, fuzzy sets, data mining methods, and random tree classifiers were the ones with the highest performance accuracy within the available data sets for the defined task (accuracy: 90%‐100%). Among the included studies, 34 papers (38%) reported the performance of AI methods using “accuracy” as the sole evaluation metric. More details on the included papers’ AI methods and performance measures are provided in the Multimedia Appendix 2.

### Mental Health Disorders

Studies focused predominantly on mood disorders and depression (16 out of 88 studies, 18.2%), with depression being the most frequently studied condition (9 out of 88 studies, 10.2%). Suicide- and self-harm-related conditions were the second most studied area (15 studies, 17%), including investigations of suicide attempts, risk, and behavior (11 out of 88 studies, 12.5%) and nonsuicidal self-injury (2 out of 88 studies, 2.3%). Neurodevelopmental disorders were examined in 11 studies (12.5%), with autism spectrum disorder being notably prevalent (8 out of 88 studies, 9.1%). Stress and psychological conditions were investigated in 10 studies (11.4%), with psychological stress/pressure being the most common (5 out of 88 studies, 5.7%). General mental health conditions were explored in 9 studies (10.2%). Substance use disorders were investigated in 7 studies (8%), including alcohol-related disorders (3 out of 88 studies, 3.4%) and cannabis use disorders (2 out of 88 studies, 2.3%). The remaining studies (20 out of 88 studies, 22.7%) covered diverse conditions, including eating disorders, school violence, psychosis, and various behavioral conditions. The details about the mental health disorders can be found in Multimedia Appendix 3.

### AI Applications on the Continuum of Adolescents’ Mental Health Care

The included papers reported on the use of AI in adolescent mental health care for the following applications: (1) 78 (89%) papers focused on the use of AI for the facilitation and improvement of the diagnostic processes, such as more accurate diagnosis, enhanced disease pathogenesis knowledge, and identification of associated disease risk factors; (2) 10 (11%) papers used AI for improving treatment by boosting treatment accuracy and implementing more targeted interventions; (3) 19 (21%) papers reported on the application of AI to improve monitoring and evaluation; and (4) 6 (6%) papers used AI for improving prognosis, for example, by discovering new disease biomarkers. More details can be found in Multimedia Appendix 3.

### Legal Information and Data Privacy

The legal information on data privacy and security using AI was not reported in any of the included papers.

### AI Delivery Mechanism

AI delivery mechanism refers to how the AI interventions were administered, for example, a smartphone app, computer software, and website. Among the included papers, 45 (50%) papers reported using different delivery mechanisms, including computer software (n=14), smartwatch (n=1), microblog (n=9), and virtual reality network interface (n=20).

### Data Type Used to Develop AI Interventions

Data type refers to the operational data acquired from patients as a result of their participation in the studies with the intent of utilizing intelligent models for a very specific process of tagging phrases, qualities, characteristics, and images [49]. The data type reported in all of the included papers is provided in Table 2.

**Table 2.** Types of data used to develop artificial intelligence interventions (N=88).

Type of data	Values, n (%)
Clinical data	41 (47)
Image and computer vision data	21 (23)
Text data	26 (30)

### Risk of Bias

**Figure 4.**
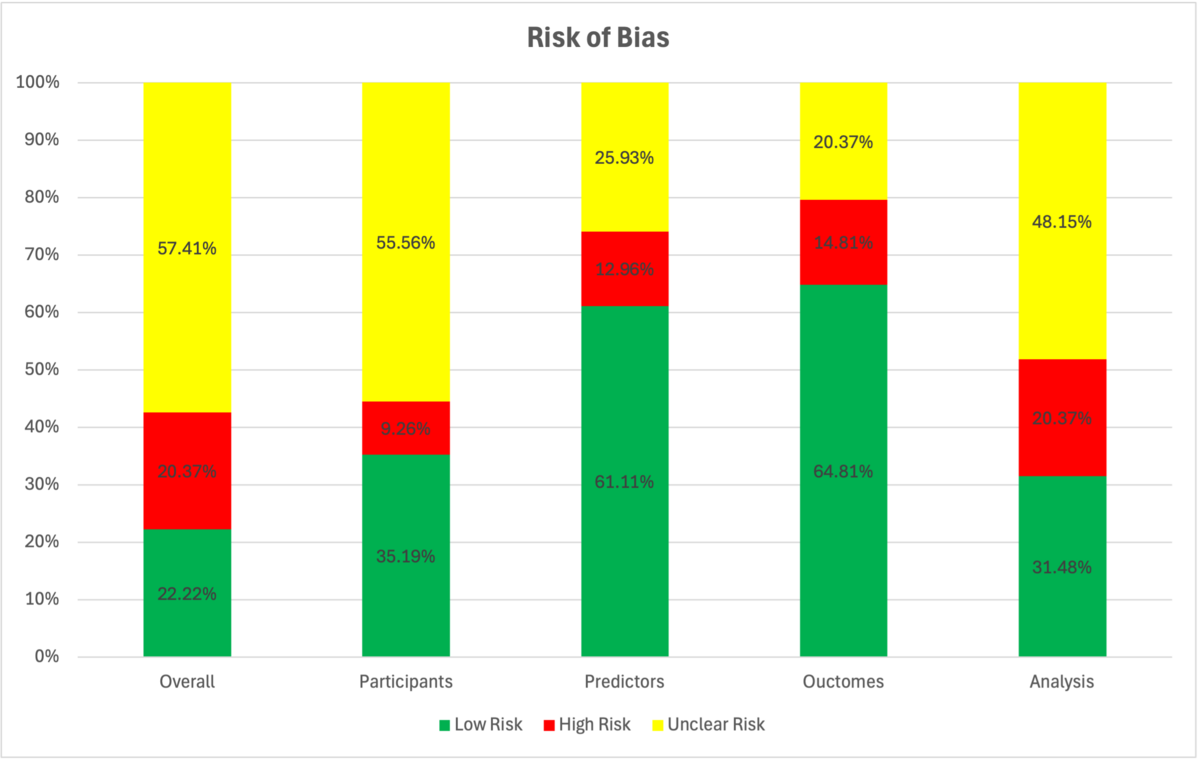
Risk of bias graph: assessing the risk of bias in 5 categories, namely overall, participants, predictors, analysis, and outcome (presented as percentages).

We identified the papers that were eligible to be evaluated using PROBAST. About 60% (53) of our included papers qualified to be analyzed using that tool. Among those, 10 (20%) papers were at high ROB according to our assessment with PROBAST (Figure 4), 31 (58%) papers were at “unclear risk” and 12 (22%) papers were at “low risk.” Details for each domain are provided in Figure 4. The detailed ROB table is available in Multimedia Appendix 4.

## Discussion

### Principal Findings

This review encompassed 88 studies examining the application of AI interventions in adolescent mental health care. These studies were critically evaluated, yielding the following key observations.

### AI Models, Performance, and Risk of Bias

ML was the most commonly used AI method in adolescents’ mental health care. Of all the methods reported, fuzzy sets, data mining, and random tree classifiers were the ones with the highest performance accuracy within the available data sets for the defined task (Accuracy: 90%‐100%). Most of the included papers (86/88, 97 %) reported on the testing and use of an off-the-shelf AI model. The findings indicate that off-the-shelf models cannot be used directly in all clinical and mental applications, given that models with extensive training data sets fail to reflect the complexities of clinical mental cohorts [50]. Studies have shown that even models trained on extensive datasets can fail to reflect real-world clinical complexities, particularly due to issues like small or nonrepresentative datasets, lack of robust validation, and insufficient adaptation to individual or cohort-specific differences [51]. Obtaining representative training data is fundamental to data-driven AI models’ performance in clinical settings. Otherwise, the data might be misinterpreted because of a lack of relevant information [52]. Furthermore, we observed a lack of standardized evaluation metrics, with 38% (34/88) of studies relying solely on accuracy. While accuracy is useful, it does not account for class imbalances or other key performance aspects such as sensitivity, specificity, and *F*_1_-score, which are critical in health care applications.

Additionally, our PROBAST assessment found that the majority (31/53, 58%) of our included studies had overall “unclear risk”. While some aspects of these studies may have been well-conducted and transparent, there was insufficient information to confidently assess the ROB in at least one of the 4 PROBAST domains (Participants, Predictors, Outcome, Analysis). Therefore, there may be potential biases or methodological issues that could affect the validity or reliability of the results, but these are not clearly documented or described. We encourage future studies to have more detailed reporting and transparency in research methodology to ensure that studies can be accurately evaluated and their findings confidently applied in practice.

In our review, we also observed that there was no mention of adherence to a particular reporting framework or guideline in any of the included papers. Studies that report on the usage of AI methods in general and in the context of physical and mental health care should follow validated frameworks or guidelines in reporting their findings [53]. Transparent reporting of AI models’ data and methodologies can facilitate identification of the possible biases and promote an accurate assessment of the efficacy, reproducibility, and reuse of AI and ML methods in future research [53].

### Mental Health Applications of AI in the Continuum of Care and Practice

We discovered AI interventions in adolescents’ mental health care in four major domains: (1) facilitating diagnostic processes such as identifying mental health related diagnostic imaging and biomarkers; (2) monitoring and evaluation such as improving follow-up visits; (3) treatment such as improving treatment accuracy; and (4) prognosis such as assisting with identifying prognostic-related biomarkers. Our findings were consistent with the previous literature on the use of AI in assisting mental health diagnosis, prognosis, treatment, and monitoring and evaluation [54]. Previous studies showed that AI interventions might enhance mental health treatments in several ways, including predicting treatment response [55], possibly avoiding or substituting impotent medication experiments [56], and invasive, costly brain stimulation therapies or time-consuming psychotherapies [56]. As for monitoring and evaluating mental health problems, AI was shown to remotely monitor and detect subjective and objective markers of psychotic recurrence [57]. Predicting whether adolescents may develop mental health problems is essential for accurate and early intervention to avoid major unfavorable consequences in the future. Additionally, studies have shown that applying AI methods to panel data, that is, data collected in a series of repeated observations of the same subjects over some extended time frame to measure the change, might help improve the accuracy of prognoses for mental health patients [58]. For instance, by combining imaging, electronic medical records, genetic, and speech data to predict depression patterns [59], risk of suicide [27], and future substance abuse among adolescents [60]. While our results showed that AI was used for the 4 main elements of diagnosis, prognosis, treatment, and monitoring and evaluation purposes in adolescents’ mental health care, most articles were concerned with the diagnosis only, and many fewer used AI in other applications. This gap needs to be addressed in future studies.

### End Users’ Involvement

Our analysis highlighted that “end users” have been slightly or inadequately involved in AI interventions’ development, validation, and testing in adolescents’ mental health care. This limited engagement with HCPs and patients during the intervention’s design process raises concerns about the suitability of AI interventions to meet their specific needs in clinical practice [61]. Involving end users in designing and developing such interventions can help produce better applications and significantly boost the likelihood of adoption. Our findings were in line with earlier research on the need to keep stakeholders in the loop when developing, testing, and validating AI interventions to screen for bias [61,62]. The failure to incorporate human elements into previous technological paradigm changes resulted in poor usage and major accidental mistakes [63]. To maximize the effectiveness and usability of AI in adolescent mental health care, it is essential to use a user-centered design approach that emphasizes the importance of involving end users throughout the entire process, including the design, development, and validation stages. Similarly, engaging diverse stakeholders throughout the AI lifecycle is important for creating effective and equitable solutions. Integrating equity, diversity, and inclusion (EDI) principles—using frameworks such as EDAI [64]—can help ensure AI systems are designed and validated with the needs and perspectives of all end users in mind, reducing bias and improving usability. Future research should prioritize EDI-oriented, user-centered approaches for adolescent mental health AI interventions.

### Biological Sex and Gender-Related Concerns

Sex and gender interplay in the emergence of diseases [65] such as depression [66], coping mechanisms [67], and influencing various factors such as diagnosis, prognosis, symptomatology expression, and therapeutic efficacy [68]. This interaction helps provide more accurate and customized recommendations tailored to each unique patient [69]. Our results indicated that the distinction between biological sex and gender-related characteristics was not reported, and few projects explicitly accounted for this when presenting demographic data. Additionally, these findings revealed that the sex distribution was specified in nearly three-fourths of the included studies, but only for adolescents and not for HCPs. None of the papers examined gender-related indicators for either patients or HCPs. These findings mirrored related research that highlighted the importance of sex and gender differences being reported explicitly as a potential source of mental and clinical heterogenicity among adolescents. This approach helps reduce health disparities and identifies the role of interindividual variances when incorporating them with AI [70-72].

### Geographic Distributions and Ethno-Racial Information

According to our results, most AI research in adolescent mental health was conducted in North American and East Asian contexts. Our findings also show that the race/ethnicity of patient participants was reported in less than a third of the included papers, with no discussion of the races/ethnicities of participating HCPs. Furthermore, for those papers that included patient ethnicity, we discovered that the obtained data were primarily associated with Caucasian/White populations, raising concerns about the dataset’s representativeness, and potential biases. Similarly, research [73] evaluating the models’ performance in predicting suicide, underlined the relevance of how ethno-racial unrepresentative datasets would impact the forthcoming AI models’ prediction results. This would lead to less benefit and more risks for patients who are Black, American Indian/Alaska Native, or whose ethno-racial demographic is unknown, compared with White, Hispanic, and Asian patients [73].

AI interventions could make predictions that discriminate against marginalized and vulnerable patient populations, leading to unsatisfactory patient outcomes due to the imbalanced health care datasets used to develop them [74,75]. Given the clear ethnic and racial differences in health care datasets (eg, disease biomarkers, prevalence, and outcomes), the inclusion of this information is likely to improve the accuracy of AI for all populations of the study [76]. By ignoring ethno-racial distinctions in the AI models, we are creating tools that do not consider diverse groups of adolescents, resulting in less effective, biased, and poor performance, particularly for minority groups with different ethnic/racial backgrounds [64]. Therefore, we propose that future research properly consider and report ethno-racial differences in AI algorithms for adolescent mental health care using frameworks such as EDAI [64].

### Limitations

We have added a new understanding of AI use in adolescent mental health care, but we also note some limitations in our research. First, since only English papers were considered, there may be publication bias, as studies published in other languages may yield different findings. Second, our search strategy may not have collected all relevant studies because we used the World Health Organization’s definition of adolescent to determine our inclusion criteria, and the definition of adolescent can still vary by country and charter. While terminologies like “adolescent,” “youth,” and “young people” are commonly used interchangeably [77,78] and may have distinct definitions in different settings/locations, this study focused mainly on the second decade (10-19 years) of life. Third, limitations in the search methodology may have resulted in the omission of certain pertinent publications, such as gray literature (eg, simulated data and robotics) and lower-level evidentiary records, including opinion pieces, editorials, comments, news, letters, and conference abstracts without full-text articles. These sources were excluded to ensure the inclusion of peer-reviewed, high-quality evidence and to minimize the risk of incorporating incomplete or nonrigorous findings. While gray literature and conference papers can provide insights into emerging AI implementations, they often lack the methodological transparency and validation required for robust analysis [79].

### Conclusions

This review provides information on the AI interventions that have been tested and implemented for use in adolescents’ mental health care. Overall, we found AI supporting adolescents’ mental health care across the included papers. In our included papers, AI has been used for various purposes in adolescent mental health care, including facilitating and enhancing diagnostic processes by enabling more accurate diagnosis, increasing our understanding of disease pathogenesis, and identifying associated risk factors. Additionally, AI has contributed to improving treatment outcomes by enhancing treatment accuracy and enabling the implementation of targeted interventions. It has also been used for enhancing monitoring and evaluation processes while aiding in prognosis by identifying novel disease biomarkers. While our results showed that AI was used for the 4 main elements of diagnosis, prognosis, treatment, and monitoring and evaluation purposes in adolescents’ mental health care, most papers were concerned with the diagnosis only, and many fewer used AI in other areas. This gap needs to be addressed in future studies. Moreover, future studies are encouraged to work on the meaningful and active involvement of end users in designing, developing, and validating AI interventions, ensuring inclusivity and diversity, and better reporting of AI models, data collection, and analysis processes. Last, as the integration of AI into health care, particularly in the context of adolescent mental health, is still emerging, it is essential to invest in education and capacity-building for HCPs, patients, and AI developers.

## Supplementary material

10.2196/70438 Multimedia Appendix 1 Full search strategy.

10.2196/70438 Multimedia Appendix 2 Some of the extracted data from the included studies.

10.2196/70438 Multimedia Appendix 3 AI application on the continuum of adolescents’ mental health care.

10.2196/70438 Multimedia Appendix 4 Risk of bias (ROB) table.

10.2196/70438 Checklist 1 Preferred reporting items for systematic reviews and meta-analyses extension for scoping reviews (PRISMA-ScR) checklist.

## Abbreviations:

ADHD: attention deficit hyperactivity disorder
AI: artificial intelligence
EPOC: Cochrane Effective Practice and Organization of Care Review Group
HCP: health care professional
ML: machine learning
PICOS: Population, Intervention, Comparison, Outcomes, Setting, and Study
PRISMA-ScR: Preferred Reporting Items for Systematic reviews and Meta-Analyses extension for Scoping Reviews
PROBAST: Prediction model Risk of Bias Assessment Tool
ROB: risk of bias
