# Supplementary material for: Use of Artificial Intelligence in Adolescents’ Mental Health Care: Systematic Scoping Review of Current Applications and Future Directions
Source: JMIR Ment Health. 2025 Jun 6;12:e70438. doi: 10.2196/70438 (PMC12165596; doi:10.2196/70438)
Supplement: Multimedia Appendix 1 [file mental-v12-e70438-s001.pdf]

Multimedia Appendix 1. Full search strategy

Table of contents:

Medline (2024-07-19) 2
Embase (2024-07-19) 5
Web of Science (2024-07-19) 8
Compendex (2024-07-19) …….. 10
INSPEC (2024-07-19) 12

Medline (2024-19-07)

PICOS	Concepts Research	Strategy keywords	Research	Research
Population	Adolescents	adolescent/ or adolescent development/ or adolescent health services/ or adolescent, institutionalized/ or adolescent, hospitalized/ or psychology, adolescent/ or adolescent psychiatry/ or adolescent medicine/ or adolescent behavior/ or adolescent health/	#1	2261638
		adolescen* or high school* or teenag* or teen age* or youth).ti,ab,kf	#2	495577
	Total	#1 or #2	#3	2401172
	Conditions	exp Mental Disorders/ or Mental Health/ or Mentally Ill Persons/	#4	1548547
		(mental* adi (disease* or disorder* or health* or ill*)).ti,ab,kf.	#5	333155
		(psychiatr* or psycholog*).ti,ab,kf.	#6	669112
	Total	#4 or #5 or #6	#7	2067644
Intervention	Artificial Intelligence (Controlled Vocabulary)	exp Artificial Intelligence/ OR DATA MINING/	#8	210629

	Artificial Intelligence (Free text)	("artificial intelligence*" or "computational intelligence*" or "machine intelligence*" or "automated reasoning" or "bayesian network*" or "bayes network*" or "naive bayes" or "bayesian learning" or "computer heuristic*" or "computer reasoning" or "data mining" or "text mining" or "expert system*" or "fuzzy logic" or "fuzzy cognitive" or "knowledge representation*" or "knowledge acquisition*" or "machine learning" or "learning machine*" or "natural language processing*" or "neural network*" or "deep learning" or "support vector*" or "hidden markov model*" or "random forest*" or "random decision forest*" or "supervised learning" or "unsupervised learning" or "autoencoder*" or "Generative adversarial network*" or "reservoir computing" or "shallow learning" or "echo state network*" or "case-based reasoning" or "metaheuristic*" or "soft computing" or "approximate reasoning" or "evolutionary computing" or "genetic algorithm*" or "bio-inspired algorithm*" or (competitive learning or learning algorithm* or fuzzy system* or clustering algorithm* or pattern classifi* or document classifi* or fuzzy inference* or fuzzy classifi* or fuzzy rule* or fuzzy control system*)).ti,ab,kf.	#9	359829
	Total 1	#8 OR #9	#10	432179
	Total 2	#3 AND #10	# 11	9630

Settings	health care setting	(clinic? or practi* or refer* or visit* or outpatient* or consult* or communit* or ambulatory or centre? or center? or office).ti,ab.	#12	4901659
		(family or physician$).af. or practiceS.mp. or primary care.af. or exp<br>Primary Health Care/ or primary.mp. or general practS.af. or gp.tw. or<br>gps.tw	#13	5262508
		(nurse$1 or social work* or dietitian* or public health or health worker* or clinical psychologist* or pediatric* or paediatric* or psychiatrist* or psychotherapist*).ti,ab,kf	#14	1273357
	Total	#12 OR #13 OR #14	#15	8776729
Total results		#3 AND #7 AND #10 AND #15	#16	776
		Limit #16 to English	#17	765

Embase (2024-19-07)

PICOS	Concepts Research	Strategy keywords	Research	Research
Population	Adolescents	exp adolescent/ or exp adolescent behavior/ or adolescent development/ or exp adolescent disease/ or adolescent health/ or adolescent obesity/ or adolescent pregnancy/ or adolescent smoking/	#1	2037408
		adolescen* or high school* or teenag* or teen age* or youth).ti,ab,kf	#2	643637
	Total	#1 or #2	#3	2221608
	Conditions	exp mental disease/ or exp mental health/ or mental patient/ or adolescent depression/	#4	3138980
		(mental* adi (disease* or disorder* or health* or ill*)).ti,ab,kf.	#5	411878
		(psychiatr* or psycholog*).ti,ab,kf.	#6	933515
	Total	#4 or #5 or #6	#7	3582630
Intervention	Artificial Intelligence (Controlled Vocabulary)	exp artificial intelligence/ or exp machine learning/ or expert system/ or fuzzy logic/ or natural language processing/ or genetic algorithm/	#8	550301

	Artificial Intelligence (Free text)	("artificial intelligence*" or "computational intelligence*" or "machine intelligence*" or "automated reasoning" or "bayesian network*" or "bayes network*" or "naive bayes" or "bayesian learning" or "computer heuristic*" or "computer reasoning" or "data mining" or "text mining" or "expert system*" or "fuzzy logic" or "fuzzy cognitive" or "knowledge representation*" or "knowledge acquisition*" or "machine learning" or "learning machine*" or "natural language processing*" or "neural network*" or "deep learning" or "support vector*" or "hidden markov model*" or "random forest*" or "random decision forest*" or "supervised learning" or "unsupervised learning" or "autoencoder*" or "Generative adversarial network*" or "reservoir computing" or "shallow learning" or "echo state network*" or "case-based reasoning" or "metaheuristic*" or "soft computing" or "approximate reasoning" or "evolutionary computing" or "genetic algorithm*" or "bio-inspired algorithm*" or (competitive learning or learning algorithm* or fuzzy system* or clustering algorithm* or pattern classifi* or document classifi* or fuzzy inference* or fuzzy classifi* or fuzzy rule* or fuzzy control system*)).ti,ab,kf.	#9	422845
	Total 1	#8 OR #9	#10	656558

Settings	health care setting	(clinic? or practi* or refer* or visit* or outpatient* or consult* or communit* or ambulatory or centre? or center? or office).ti,ab,kf.	#11	7045910
		(family or physician$).af. or practiceS.mp. or primary care.af. or exp Primary Health Care/ or primary.mp. or general practS.af. or gp.tw. or gps.tw	#12	7045910
	Total	#11 OR #12	#13	11581770
Total results		#3 AND #7 AND #10 AND #13	#14	1406
		Limit #14 to English	#15	1385

Web of Science (2024-19-07)

PICOS	Concepts Research	Strategy keywords	Research	Results
Population		TS=(adolescen* OR "high school*" OR teenag* OR "teen age*" OR youth)<br>Indexes= WOS.SCI,WOS.ISTP,WOS.BSCI,WOS.ESCI<br>Timespan=All years	#1	626344
	Conditions	TS=((mental* NEAR1 (disease* OR disorder* OR health* OR ill*)) OR psychiatr* OR psycholog*)<br>Indexes= WOS.SCI,WOS.ISTP,WOS.BSCI,WOS.ESCI<br>Timespan=All years	#2	918333
Intervention	Artificial Intelligence (Free text)	TS=("artificial intelligence*" OR "computational intelligence*" OR "machine intelligence*" OR "automated reasoning" OR "bayesian network*" OR "bayes network*" OR "naive bayes" OR "bayesian learning" OR "computer heuristic*" OR "computer reasoning" OR "data mining" OR "text mining" OR "expert system*" OR "fuzzy logic" OR "fuzzy cognitive" OR "knowledge representation*" OR "knowledge acquisition*" OR "machine learning" OR "learning machine*" OR "natural language processing*" OR "neural network*" OR "deep learning" OR "support vector*" OR "hidden markov model*" OR "random forest*" OR "random decision forest*" OR "supervised learning" OR "unsupervised learning" OR "autoencoder*" OR "Generative adversarial network*" OR "reservoir computing" OR "shallow learning" OR "echo state network*" OR "case-based reasoning" OR "metaheuristic*" OR "soft computing" OR "approximate reasoning" OR "evolutionary computing" OR "genetic algorithm*" OR "bio-inspired algorithm*" OR ("competitive learning" OR "learning algorithm*" OR "fuzzy system*" OR "clustering algorithm*" OR "pattern classifi*" OR "document classifi*" OR "fuzzy inference*" OR "fuzzy classifi*" OR "fuzzy rule*" OR "fuzzy control system*"))<br>Indexes= WOS.SCI,WOS.ISTP,WOS.BSCI,WOS.ESCI	#3	1865779

Settings	health care settings	TS=("clinic" OR "clinics" OR practi* OR refer* OR visit* OR outpatient* OR consult* OR communit* OR ambulatory OR centre* OR center* OR office OR family OR physician* OR primary OR "gp" OR "gps" OR "nurse" OR "nurses"<br>OR "social work*" OR dietitian* OR "public health" OR "health worker*" OR<br>"clinical psychologist*" OR pediatric* OR paediatric* OR psychiatrist* OR psychotherapist*<br>Indexes=WOS.SCI,WOS.ISTP,WOS.BSCI,WOS.ESCI<br>Timespan-=All years	#4	13161059
Total results		#4 AND #3 AND #2 AND #1 AND LANGUAGE: (English)<br>Indexes= WOS.SCI,WOS.ISTP,WOS.BSCI,WOS.ESCI<br>Timespan=All years	#5	372

Compendex (2024-19-07)

PICOS	Concepts Research	Strategy Keywords	Research	Results
Total results		(((("clinic" OR "clinics" OR practi* OR refer* OR visit* OR outpatient* OR consult* OR communit* OR ambulatory OR centre* OR center* OR office OR family OR physician* OR primary OR "gp" OR "gps" OR "nurse" OR "nurses" OR "social work*" OR dietitian* OR "public health" OR "health worker*" OR "clinical psychologist*" OR pediatric* OR paediatric* OR psychiatrist* OR psychotherapist*) AND ("artificial intelligence*" OR "computational intelligence*" OR "machine intelligence*" OR "automated reasoning" OR "bayesian network*" OR "bayes network*" OR "naive bayes" OR "bayesian learning" OR "computer heuristic*" OR "computer reasoning" OR "data mining" OR "text mining" OR "expert system*" OR "fuzzy logic" OR "fuzzy cognitive" OR "knowledge representation*" OR "knowledge acquisition*" OR "machine learning" OR "learning machine*" OR "natural language processing*" OR "neural network*" OR "deep learning" OR "support vector*" OR "hidden markov model*" OR "random forest*" OR "random decision forest*" OR "supervised learning" OR "unsupervised learning" OR "autoencoder*" OR "Generative adversarial network*" OR "reservoir computing" OR "shallow learning" OR "echo state network*" OR "case-based reasoning" OR "metaheuristic*" OR "soft computing" OR "approximate reasoning" OR "evolutionary computing" OR "genetic algorithm*" OR "bio-inspired algorithm*" OR ("competitive learning" OR "learning algorithm*" OR "fuzzy system*" OR "clustering algorithm*" OR "pattern classifi*" OR "document classifi*" OR "fuzzy inference*" OR "fuzzy	#1	411

		classifi*" OR "fuzzy rule*" OR "fuzzy control system*")) AND (adolescen* OR "high school*" OR teenag* OR "teen age*" OR youth) AND ((mental 0NEAR/1 disease) OR (mental 0NEAR/1 disorder) OR (mental 0NEAR/1 health) OR (mental 0NEAR/1 illness) OR psychiatr* OR psycholog*))) AND (((cpx) WN DB) AND ({english} WN LA)))		

INSPEC (2024-19-07)

PICOS	Concepts Research	Strategy Keywords	Research	Results
Total Results		(((("clinic" OR "clinics" OR practi* OR refer* OR visit* OR outpatient* OR consult* OR communit* OR ambulatory OR centre* OR center* OR office OR family OR physician* OR primary OR "gp" OR "gps" OR "nurse" OR "nurses" OR "social work*" OR dietitian* OR "public health" OR "health worker*" OR "clinical psychologist*" OR pediatric* OR paediatric* OR psychiatrist* OR psychotherapist*) AND ("artificial intelligence*" OR "computational intelligence*" OR "machine intelligence*" OR "automated reasoning" OR "bayesian network*" OR "bayes network*" OR "naive bayes" OR "bayesian learning" OR "computer heuristic*" OR "computer reasoning" OR "data mining" OR "text mining" OR "expert system*" OR "fuzzy logic" OR "fuzzy cognitive" OR "knowledge representation*" OR "knowledge acquisition*" OR "machine learning" OR "learning machine*" OR "natural language processing*" OR "neural network*" OR "deep learning" OR "support vector*" OR "hidden markov model*" OR "random forest*" OR "random decision forest*" OR "supervised learning" OR "unsupervised learning" OR "autoencoder*" OR "Generative adversarial network*" OR "reservoir computing" OR "shallow learning" OR "echo state network*" OR "case-based reasoning" OR "metaheuristic*" OR "soft computing" OR "approximate reasoning" OR "evolutionary computing" OR "genetic algorithm*" OR "bio-inspired algorithm*" OR ("competitive learning" OR "learning algorithm*" OR "fuzzy system*" OR "clustering algorithm*" OR "pattern classifi*" OR "document classifi*" OR "fuzzy inference*" OR "fuzzy	#1	236

		classifi*" OR "fuzzy rule*" OR "fuzzy control system*")) AND (adolescen* OR "high school*" OR teenag* OR "teen age*" OR youth) AND ((mental 0NEAR/1 disease) OR (mental 0NEAR/1 disorder) OR (mental 0NEAR/1 health) OR (mental 0NEAR/1 illness) OR psychiatr* OR psycholog*))) AND (((ins) WN DB) AND ({english} WN LA)))		
