# Supplementary material for: Use of Artificial Intelligence in Adolescents’ Mental Health Care: Systematic Scoping Review of Current Applications and Future Directions
Source: JMIR Ment Health. 2025 Jun 6;12:e70438. doi: 10.2196/70438 (PMC12165596; doi:10.2196/70438)
Supplement: Multimedia Appendix 2 [file mental-v12-e70438-s002.pdf]

**Multimedia Appendix 2:** Some of the extracted data from the included studies

| Title | Objective | Input information | Performance | AI Methods | Compared with other methods? | Eligible for PROBAST (Yes/No) |
|---|---|---|---|---|---|---|
| EEG classification of adolescents with type I and type II of bipolar disorder [1] | To classify the EEG signal of adolescents with BD I from BD II using different analysis including neural network | Electroencephalogram signal | Best average accuracy rate for testing data set was for DISR: 91.83 ± 0.41 % | Multilayer Perceptron Neural Networks (MIM, CMIM, FCBF and DISR) | Yes | Yes |
| Virtual reality-based facial expressions understanding for teenagers with autism [2] | To present an analysis of physiological as well as eye tracking data from a usability study aimed at evaluating the efficacy of an innovative VR-based system for facial emotional expression identification task | Physiological signals | 94% for Gaussian mixture and 91% for k-means | Gaussian mixture and k-means clustering methods (machine learning algorithm) | No | No |
| Integrating human mobility and social media for adolescent psychological stress detection [3] | To propose a co-training-based stress detection model based on teens' daily GPS trajectories and microblog data. | Positional and temporal outlier features from teens' daily trajectories to facilitate stress detection. | Best accuracy: 88.92%, Best F1-measure: 91.02% | Compared different frameworks and come up with a new result (Co-training framework) =>One classifier is | Yes | Yes |

| | | | | | | |
|---|---|---|---|---|---|---|
| | | | | conditional random field (CRF), The other classifier is deep neural network (DNN), random forest (RF), Support Vector Machine, NB (Naive Bayes), GBDT (Gradient Boosted Decision Tree), Co Training (considering all methods together) | | |
| Evaluation on life satisfaction of left-behind junior high school children based on LVQ network [4] | To evaluate the life satisfaction of left-behind children using neural network and classify them with less labor and time. | Data obtained from Chinese adolescent students' life satisfaction scale. | The absolute and relative correct rate of prediction were 97.8% and 100%, and the trained network exhibited high performance. | Learning Vector Quantization Neural Network (LVQ NN) | No | Yes |
| Adolescents psychological well-being estimation based on a data mining algorithm [5] | To create a software that allows to estimate a psychological well-being of adolescents 12-17, based on a questionnaire autonomously, without participation of a psychologist 2) reduce, | Responses of about 200 adolescents aged 12-17 years from 11 rural schools. | Accuracy of determining psychological well-being is already at the level of 72-91% | Group method of data handling (GMDH) which is called twice-multilayered modified polynomial neural networks | No | Yes |

| | | | | | | |
|---|---|---|---|---|---|---|
| | correct and expand the number/kind of questions used in assessing the adolescents psychological well-being, using machine learning methods. | | | | | |
| Detecting Adolescent Psychological Pressures from Micro-Blog [6] | To propose a pressure detection strategy on micro-blog to timely and effective detect teenagers' psychological pressures status and psychological pressures changes. | Teenagers' tweets on micro blog platform | Gaussian Process: Showed best accuracy (highest precision and recall rates) between 0.826-0.820-0.823 | Tested five classifiers (Naive Bayes, Support Vector Machines, Artificial Neural Network, Random Forest, and Gaussian Process Classifier) | Yes | Yes |
| The clinical application of fMRI data in a single-patient diagnostic conundrum: Classifying brain response to experimental pain to distinguish between gastrointestinal, depressive and eating disorder symptoms. [7] | Used a novel machine learning approach to classify (i) the patient's functional brain imaging during an experimental pain paradigm, and (ii) patient self-report psychological measures, to categorize the diagnostic phenotype most closely approximated by the patient. | Using age as a covariate, created pain-related brain activation masks in the 23 healthy control women who previously performed the same task, characterizing brain response to high versus low pain, and anticipation of | 1) Accuracy of Support Vector Machine (SVM) for 'brain activation/brain-based classifications: 84% 2) Accuray of SVM for 'psychological (behavioural) variables/based classifications': 54% | Support Vector Machine | No | Yes |

| | | | | | | |
|---|---|---|---|---|---|---|
| | | high versus low pain. | | | | |
| Frequency-specific functional connectivity density as an effective biomarker for adolescent generalized anxiety disorder [8] | 1) To investigate how Generalized Anxiety Disorder (GAD) affects the brain's short range functional connectivity density S-FCD (Functional Cognitive Disorder) and short range functional connectivity density and L-FCD in adolescents across different frequency bands 2) To examine whether the frequency-specific Functional Connectivity Density (FCD)could help to discriminate patients with GAD from Healthy Controls | Data from 31 adolescent patients with GAD and 28 matched healthy controls; | Performance measure: Best Support Vector Machine (SVM) classification performance was derived from combining both S-FCD and L-FCD in which the AUC value of 0.9414 and the corresponding sensitivity, specificity, and accuracy of 87.15, 92.92, and 89.83%, respectively | Support Vector Machine | No | Yes |
| Multivariate classification of autism spectrum disorder using frequency-specific resting-state functional connectivity-A multi-center study [9] | To use Multivariate pattern analysis (MVPA), by the virtue of multi-centre datasets to find a robust objective neuroimage biomarker to diagnose ASD. | Data of adolescents with autism spectrum disorder. | A relatively high classification accuracy of 79.17% (AUC: 0.7917) (77.78% for sensitivity, 80.47% for specificity, permutation test | Support Vector Machine | No | Yes |

| | | | | | | |
|---|---|---|---|---|---|---|
| | | | p b 0.001, 1000 times) | | | |
| Identification of chief characteristics of alcohol consumption traits in schools using rough set and formal concept analysis [10] | To extract the major attributes that correlates most to a teenager's drinking habits from the Portuguese high school alcohol consumption dataset. | Reports from schools that were completed using questionnaires. | 1) For Alcoholic trait: 100 accuracy 2) For non-alcoholic trait: 90-100% | Fuzzy sets- Data mining | No | Yes |
| Cortical thickness predicts the first onset of major depression in adolescence [11] | To predict the future onset of depression in adolescents using machine learning based on baseline cortical thickness from MRI scans. It investigates whether brain structure can serve as an early biomarker for depression risk. | MRI Data | Performance measure: Overall accuracy of 69.7% (69.3 % sensitivity, 70% specificity; $p = 0.021$) | Support Vector Machine | No | Yes |
| Pattern classification of response inhibition in ADHD: Toward the development of neurobiological markers for ADHD [12] | 1) Gaussian Process Classifiers (GPCs) of task-based fMRI data during the tracking Stop task in 30 boys with and 30 healthy boys can identify distributed neurofunctional patterns that will provide accurate | fMRI data | Performance measures: Overall classification accuracy of 77% (sensitivity of 90% and specificity of 63%, ($P < 0.001$, positive predictive value | Gaussian process classifiers (GPC) | Yes | Yes |

| | | | | | | |
|---|---|---|---|---|---|---|
| | diagnostic predictors of ADHD. 2) To use traditional univariate analyses to replicate previous findings of reduced function in inhibitory regions of ventrolateral prefrontal cortex (VLPFC) and the basal ganglia in a relatively large cohort of 30 ADHD patients. | | (PPV) was 71.05% and the negative predictive value (NPP) was 86.36%) | | | |
| Multiparametric MRI characterization and prediction in autism spectrum disorder using graph theory and machine learning [13] | 1) To evaluate multi-parametric functional and structural MRI of the brain in autism spectrum disorder (ASD) versus typically developed (TD) children using small-world network analysis based on graph theory to derive local and global efficiency, 2) To use machine-learning algorithms to evaluate the ability of these multiparametric MRI matrices to classify ASD versus TD groups, and 3) To employ machine-learning algorithms of these | MRI data | The ‘random tree classifier’ had the highest classification accuracy (100%), (close to 100% sensitivity and specificity for correctly identifying ASD patients with the full dataset, and 70% accuracy for differentiating ASD patients from TD children using 80% percentage split cross | To differentiate the two groups based on the selected imaging features, a total of 67 available classifiers, including support vector machine (SVM), Bayes network (BayesNet), radial basis function (RBF), and sequential minimal optimization (SMO) algorithms, were tested with batch-mode scripts developed in WEKA software. | Yes | Yes |

| | | | | | | |
|---|---|---|---|---|---|---|
| | multiparametric MRI matrices to predict ASD clinical phenotypic outcomes, such as the revised autism diagnostic interview (ADI-R), autism diagnostic observation schedule (ADOS), and intelligence quotient (IQ) scores reflecting different aspects of social and learning abilities of subjects | | validation. Based on the 4 imaging features, the random tree classifier also had the highest accuracy (98%) for the full dataset for two-group classification, with 68% accuracy for 10-fold cross validation. | The 'random tree classifier' was another AI technique used among 67 classifiers. | | |
| Predicting Depression in Adolescents Using Mobile and Wearable Sensors: Multimodal Machine Learning–Based Exploratory Study [14] | To predict depression scores and changes in depression levels in adolescents using passively sensed mobile and wearable data. | Smartphone and Fitbit sensor data (calls, location, screen use, heart rate, etc.) and weekly PHQ-9 depression scores from 55 adolescents. | Best RMSE was 2.83 for depression score prediction and 3.21 for change in depression level using personalized models. | Linear (LASSO, Elastic Net) and nonlinear (Random Forest, XGBoost, AdaBoost) regression-based models with personalized strategies. | Yes | No |
| Quantification of the influence of friends and antisocial behaviour in adolescent | To analyze and quantify the predictive value of different personal, family and environmental variables on the consumption of | Number of joints consumed per week, consumption by the peer | Not reported. | Poisson regression model (PRM) and a data mining using a decision tree (DT) | No | Yes |

| | | | | | | |
|---|---|---|---|---|---|---|
| consumption of cannabis using the ZINB model and data mining. [15] | cannabis in adolescence. | group, nights out during the week, gender, the production of forbidden behaviour and the use of other substances. | | | | |
| Study of the factors associated with substance use in adolescence using Association Rules [16] | To analyze the factors related to the use of addictive substances in adolescence using association rules, descriptive tools included data mining. | The adolescents answered an anonymous questionnaire on personal, family and environmental risk factors related to substance use. | The best rules predicted substance use with confidence scores ranging from 0.8032 to 1.0000, depending on the substance and associated factors | Data mining | No | No |
| Relationship of personality, environmental, and DICA variables to adolescent hopelessness: A neural network sensitivity approach [17] | To identify critical factors from a set of psychiatric diagnoses, personality traits, and family and social support variables that relate to hopelessness in adolescents. | Data from millon adolescent personality inventory, the parental bonding questionnaire, the social support questionnaire, the hopelessness scale for | Accuracy more than 80%, the neural network model was able to obtain a good fit for the data (R2 = .9908), whereas the linear regression did not (R2 = .3316). | Neural Networks | Yes | Yes |

| | | | | | | |
|---|---|---|---|---|---|---|
| | | children and were interviewed by trained clinicians on the diagnostic interview for children and adolescents. | | | | |
| Detecting dysfunctional behavior in adolescents: the examination of relationships using neural networks [18] | To identify the most important mental health variables from a set of personality, family support, and social support variables that related to hopelessness by modeling these relationships using a neural network | Data collected from Millon Adolescent Personality Inventory (MAPI) the Diagnostic Interview for Children and Adolescents (DICA);the Parental Bonding Questionnaire; the Social Support Questionnaire, and the Hopelessness Scale for children. | The neural network predicted hopelessness with 79% accuracy within 25% error and 93% within 50% error. | Artificial Neural Networks | No | Yes |
| Predicting Teenager's Future | To predict teenagers' future adolescent stress level from micro-blog. | Data from micro-blog | Nearest mean approach=> Error:  0.7161- | Nearest mean approach, Linear interpolation, | No | Yes |

| | | | | | | |
|---|---|---|---|---|---|---|
| Stress Level from Micro-Blog [19] | | | 0.7501 2) Linear interpolation => Error: 0.7755-0.8036 3) Exponential smoothing=> 4) Gaussian Process Regression(GPR)=> Error: 0.4794-0.4973 5) Supported Vector Regression (SVR)=> Error: 0.5429- 0.5656 Gaussian Process Regression (GPR) showed the lowest possible error compared to other methods! (GPR error: 0.4794-0.4973). Error for the rest of the ML methods used were more than 50%. | Exponential smoothing, Gaussian Process Regression (GPR), Supported Vector Regression (SVR) | | |

| | | | | | | |
|---|---|---|---|---|---|---|
| Predictors of Adolescents' First Episode of Homelessness Following Substance Use Treatment [20] | To identify predictors of youths' first episode of homelessness during the 12 months after substance use treatment entry. | Data from survey on children using receiving substance use treatment. | Not reported. | LASSO machine learning regression | No | Yes |
| Adolescent binge drinking disrupts normal trajectories of brain functional organization and personality maturation [21] | To identify the disrupting effects of adolescent binge drinking on the developmental trajectories of both brain and personality. | Information about brain functional architecture, personality traits, and genetic variants in 19-year-old individuals. | Accuracy: 71.2%, AUC: 0.900 | Support Vector Machine | No | Yes |
| Identifying suicidal adolescents from mental health records using natural language processing [22] | 1) To generate a manually annotated reference standard of an adolescent cohort that was inclusive of all mental health conditions and 2) To apply and modify an existing Natural Language Processing (NLP) approach for mention-level extraction of specified clinical constructs. | EHR documentation related to suicide risk | 1) Inter-annotator agreement on the subset of 100 documents was very high: 0.96, 98% accuracy, >80% f1 score at both document and patient level 2) Recall results: ranged from 74% to 90% | Natural Language Processing (NLP) | No | Yes |
| Negative Symptoms in early-onset | (1) The prevalence of Negative Symptoms (NS) at first | Data extracted from the electronic | PPV 0.91, recall 0.73 | Natural Language Processing (NLP) | No | Yes |

| | | | | | | |
|---|---|---|---|---|---|---|
| psychosis and their association with antipsychotic treatment failure. [23] | presentation to mental health services and (2) Whether NS predicted eventual development of multiple treatment failure (MTF) prior to the age of 18. | health records having Marder Factor NS and antipsychotic use. | | | | |
| Dissociable psychosocial profiles of adolescent substance users. [24] | To examine the role of the individual, family, school, peer, and social environment on alcohol (lifetime and risky), tobacco (risky only), and cannabis use (lifetime and riskiness). | Substance use behavior alongside risk and protective factors across individual, family, school, peer and social domains were used. | Accuracy more than 88%, Model A (Lifetime Alcohol): Precision: 0.8106 Recall: 0.8326 AROC: 0.8924 F1 Score: 0.8101 , Model B (Alcohol Risk): Precision: 0.6816 Recall: 0.8810 AROC: 0.9051 F1 Score: 0.7688 , Model C (Tobacco Risk): Precision:0.5437 Recall: 0.8979 AROC: 0.8814 F1 Score: 0.6723 , Model D (Cannabis Lifetime): | Logistic regression with Elastic Net regularization (LASSO, ridge regression) | No | Yes |

| | | | | | | |
|---|---|---|---|---|---|---|
| | | | Precision: 0.4484 Recall: 0.9314 AROC: 0.9156 F1 Score: 0.6039, Model E (Cannabis Risk): Precision: 0.3100 Recall: 0.9606 AROC: 0.9247 F1 Score: 0.4687 | | | |
| Type and timing of childhood maltreatment and reduced visual cortex volume in children and adolescents with reactive attachment disorder. [25] | To investigate the effect of type and timing of childhood adversities on structural alterations in regional gray matter (GM) volume in maltreated children with RAD. | High-resolution magnetic resonance imaging datasets with RAD | Only information available regarding accuracy: Reasonable accuracy based on type of maltreatment and number of types of maltreatment ($r = 0.650$, $p < 0.05$). | Random forest regression with conditional inference trees | No | Yes |
| Decreased resting-state interhemispheric functional connectivity correlated with neurocognitive | To examine functional connectivity between homotopic brain regions in drug-naïve, first-episode patients with adolescent onset schizophrenia (AOS). | Data from MRI scans | Sensitivity of 100%, specificity of 87.09%, and accuracy of 94.93% | Support Vector Machine | No | No |

| | | | | | | |
|---|---|---|---|---|---|---|
| deficits in drug-naive first-episode adolescent-onset schizophrenia. [26] | | | | | | |
| Prediction of outcome in internet-delivered cognitive behaviour therapy for paediatric obsessive-compulsive disorder: A machine learning approach [27] | To test four different machine learning methods in the prediction of treatment response in a sample of paediatric OCD patients who had received internet-delivered cognitive behaviour therapy (ICBT). | Clinical baseline variables were used to predict strictly defined treatment response status three months after ICBT. | 1) A linear model: 83% % [95% confidence interval (CI) (52–98%)]- 2) L1 Elastic Net (Lasso): Accuracy in the test sample was 75% [95% CI (43–95%)]- 3) Random Forests: Accuracy was 75% [95% CI (43–95%)]- 4) Support Vector Machines: Accuracy was 75% [95% CI (43–95%)]- . All Accuracies between 75 to 83% | 1) A linear model 2) L1 Elastic Net (Lasso), 3) Random Forests 4) Support Vector Machines | Yes | Yes |
| Applying deep neural networks to unstructured text notes in electronic medical records | To use Natural Language Processing (NLP) and ML to identify the phenotype of interest: youth | EMR documents | 1) A Brute Force search method using Natural Language Processing | 1) A brute force search method using Natural Language Processing (NLP) | No | Yes |

| | | | | | | |
|---|---|---|---|---|---|---|
| for phenotyping youth depression [28] | patients ages 12–18 with DSM-IV (Diagnostic and Statistical Manual of Mental Disorders) defined major depressive disorder or dysthymic disorder. | | (NLP) package: sensitivity=80%, specificity=88% ) sensitivity 93.5%; specificity 68%; positive predictive value (precision) 77%) DL0_2: 87% accurate | package: 2) Deep Neural Network using NLP package | | |
| Neural Predictors of Initiating Alcohol Use During Adolescence [29] | Identifying variables that can generate individual-level predictions of initiating alcohol use during adolescence by age 18 (Underaged drinking) | Mix of demographic, behavioral, neuropsychological, and neuroimaging data | 74% accurate, with sensitivity (74%) and specificity (73%) | Random forest classification model | No | Yes |
| Prediction Models for suicide attempts among adolescents using machine learning techniques [30] | To develop and evaluate suicide attempts prediction models based on six different machine learning (ML) algorithms for Korean adolescents using data from online surveys. | Data were extracted from the 2011-2018 Korea Youth Risk Behavior Survey (KYRBS). | The performance of the six ML models on the internal testing dataset was good, with both the area under the receiver operating characteristic curve (AUROC) and area under the precision-recall curve | Classic ML (CML) methods, namely logistic regression (LR), random forest (RF), artificial neural networks (ANN), support vector machines (SVM), and extreme gradient boosting (XGB) | Yes | Yes |

| | | | | | | |
|---|---|---|---|---|---|---|
| | | | (AUPRC) ranging from 0.92 to 0.94. Although the AUROC of all models on the external testing dataset (2018 KYRBS) ranged from 0.93 to 0.95, the AUPRC of the models was approximately 0.5. | | | |
| Large language models perform on par with experts identifying mental health factors in adolescent online forums [31] | To investigate performance of two top-performing LLMs (GPT3.5 and GPT4) on extracting mental health factors in adolescent social media posts to verify whether they can be on par with expert annotators. | Reddit Posts | GPT-4 demonstrated performance comparable to human inter-annotator agreement, with notably higher results on synthetic data. However, the model occasionally struggles with negation and factuality. The superior performance on | LLM (GPT 3.5 and 4.0) | Yes | No |

| | | | | | | |
|---|---|---|---|---|---|---|
| | | | synthetic data appears to stem from the increased complexity of real-world data rather than any inherent model advantage. GPT-3.5 achieved average results at the category level, performing better on Positive Only metrics (0.57). In contrast, GPT-4 outperforms GPT-3.5, particularly in Positive Only metrics (0.63) and subcategory accuracy (0.48 vs. 0.39). | | | |
| The cross-sectional study of depressive symptoms and associated factors among adolescents | To investigate the association between depressive symptoms and diet- and lifestyle-related behaviors among adolescents | Data from Survey using the Center for Epidemiological Studies | Not reported. | Backpropagation (BP) neural network model | No | No |

| | | | | | | |
|---|---|---|---|---|---|---|
| by backpropagation neural network [32] | | Depression Scale | | | | |
| Dimensional measures of psychopathology in children and adolescents using large language models [33] | To estimate dimensional psychopathology from narrative clinical notes | EMR narrative clinical notes | Linear regression best fit: Kendall's rank correlation tau = 0.14, 0.22, 0.17, 0.21, and 0.15 for arousal, cognitive, negative, positive, and social domains, respectively; all ps < .001. Pearson's r = 0.24, 0.34, 0.30, 0.32, and 0.28, respectively; all ps < .001. | LLM (chatGPT 4) | Yes | No |
| A novel application of a data mining technique to study intersections in the social determinants of mental health among young Canadians [34] | To evaluate a new data mining technique to identify social locations of young Canadians where differences in mental health between adolescent males and females were most pronounced | 2018 National health and health behaviours survey | Not reported. | Recursive partitioning for subgroup identification (SIDES) | No | No |

| | | | | | | |
|---|---|---|---|---|---|---|
| Prevention of Suicidal Relapses in Adolescents With a Smartphone Application: Bayesian Network Analysis of a Preclinical Trial Using In Silico Patient Simulations [35] | To present our development of a Bayesian Network (BN) algorithm as a medical device in accordance with the American Psychiatric Association digital healthcare guidelines and to provide results from a preclinical phase | | Not reported. | Bayesian Network | No | No |
| Identify adolescents' help-seeking intention on suicide through self- and caregiver's assessments of psychobehavioral problems: deep clustering of the Tokyo TEEN Cohort study [36] | To identify adolescents' help seeking intention on suicide through self and caregiver's assessment on psychobehavioural problems. | Self-reported questionnaires | Odds ratio and z-score; The results were robust in the sensitivity analysis: the inclusion of participants who became untraceable during the follow-up surveys, the clustering without standardizing binary variables, and the omission of missing value imputation for psychopathologi | Deep learning model | No | No |

| | | | | | | |
|---|---|---|---|---|---|---|
| | | | cal assessments in clustering and for predictors in regression analysis | | | |
| Predicting suicide attempts and suicide deaths among adolescents following outpatient visits [37] | To examine whether a model trained and validated using data from all age groups works as well for adolescents or whether it could be improved in regards to suicide attempts and deaths in outpatient visits | EMR of outpatient visits | The AUC produced by the existing model for specialty mental health visits estimated in adolescents alone (0.796; [0.789, 0.802]) was not significantly different than the AUC of the recalibrated existing model (0.794; [0.787, 0.80]) or the newly-learned model (0.795; [0.789, 0.801]). Predicted risk following primary care visits was also similar: existing (0.855; [0.844, 0.866]), recalibrated | LASSO (least absolute shrinkage and selection operator) variable + generalized estimating equations (GEE) selection | Yes | Yes |

| | | | | | | |
|---|---|---|---|---|---|---|
| | | | (0.85 [0.839, 0.862]), newly-learned (0.842, [0.829, 0.854]). | | | |
| Assessment and Prediction of Depression and Anxiety Risk Factors in Schoolchildren: Machine Learning Techniques Performance Analysis [38] | To use machine learning techniques to predict the risk factors associated with schoolchildren's depression and anxiety | Questionnaire data | The results indicated that the support vector machine (SVM) and random forest (RF) models had the highest accuracy levels for depression (SVM: 92.5%; RF: 76.4%) and anxiety (SVM: 92.4%; RF: 78.6%) | 5 machine learning techniques (random forest [RF], neural network, decision tree, support vector machine [SVM], and naive Bayes) | Yes | Yes |
| Machine learning techniques for identifying mental health risk factor associated with schoolchildren cognitive ability living in politically violent environments [39] | To examine the impact of living in politically violent environments on the mental health and cognitive development of children | The dataset consists of primary data extracted from the national Health Behavior in School-Aged Children (HBSC) study | The results indicated that the random forest (RF) had the highest performance of balanced accuracy (87%), followed by neural network and SVM. While the lowest balanced accuracy rate | Gradient Boosting (GB), Support Vector Machine (SVM), Random Forest (RF), Artificial Neural Network (ANN), k-Nearest Neighbors (kNN), and Decision Tree (DT) algorithms | Yes | No |

| | | | | | | |
|---|---|---|---|---|---|---|
| | | | was found in the kNN algorithm. All ML models used in our analysis showed an accuracy rate (F1-score) above 75% in identifying cognitive ability. It was determined that the RF algorithm’s predictive power differed significantly from that of the other models | | | |
| A Bayesian learning model to predict the risk for cannabis use disorder (CUD) [40] | To help stem this public health concern, a model is needed that predicts for an adolescent or young adult cannabis user their personalized risk of developing CUD in adulthood. | Add Health database | The AUC of this model was 0.69 and its E/O was 0.953. For external validation: The AUC was 0.71 and the E/O was 1.10. | Bayesian Network | Yes | Yes |
| What Factors Are Most Closely Associated With Mood Disorders in | To explore the importance of these factors in the development of mood | Questionnaire | Gradient-boosted decision tree (GBDT) + Logistic | *k*-nearest neighbors (KNN), logistic regression (LR), gradient- | No | No |

| | | | | | | |
|---|---|---|---|---|---|---|
| Adolescents During the COVID-19 Pandemic? A Cross-Sectional Study Based on 1,771 Adolescents in Shandong Province, China [41] | disorders in adolescents during the pandemic | | Regression (LR) model had the best performance for both the GAD-7 (average AUC = 0.819) and PHQ-9 (average AUC = 0.857) | boosted decision tree (GBDT), and a combination of the GBDT and LR (GBDT+LR). | | |
| Predicting Adolescent Mental Health Outcomes Across Cultures: A Machine Learning Approach [42] | To demonstrate how data- and theory-driven methods can be integrated to identify the most important preadolescent risk factors in predicting adolescent mental health | Data were collected from the Parenting Across Cultures Project | Accuracy | Support Vector Machines (SVMs) with linear kernel | No | Yes |
| A Comparative Analysis of Decision Tree and Support Vector Machine on Suicide Ideation Detection [43] | To increase the capabilities of Decision Tree and Support Vector Machine algorithm, enhancing the performance of already existing research about suicide ideation detection with improved accuracy. | Reddit forum | Accuracy, precision, recall, F1, AUC; Decision tree: 81.57 82.97 79.84 81.37 81.58; SVM: 90.80 93.96 87.38 90.55 96.77 | Decision tree and support vector algorithm | Yes | No |
| Investigating online activity in UK adolescent mental health | To assess the feasibility of using a natural language processing (NLP) application for | EMR | The Natural Language Processing (NLP) | Natural Language Processing (NLP) | No | No |

| | | | | | | |
|---|---|---|---|---|---|---|
| patients: A feasibility study using a natural language processing approach for electronic health records [44] | extraction of free-text online activity mentions in adolescent mental health patient electronic health records (EHRs) | | application performed with good precision (0.97) and recall (0.94) for identification of online activity mentions. | | | |
| Exploring Korean adolescent stress on social media: a semantic network analysis [45] | To provide basic data to establish desirable stress coping strategies for adolescents based on a big data-based network analysis of social media for Korean adolescent stress. | Social media and blogs | Not reported. | Data based network analysis | No | No |
| Natural Language Processing Insight into LGBTQ+ Youth Mental Health During the COVID-19 Pandemic: Longitudinal Content Analysis of Anxiety-Provoking Topics and Trends in Emotion in LGBTeens Microcommunity Subreddit [46] | To address this knowledge gap by harnessing natural language processing methodologies to investigate the evolution of conversation topics in the most popular subreddit for LGBTQ+ youth. | Subreddit posts | Not reported. | Natural Language Processing (NLP) | No | No |

| | | | | | | |
|---|---|---|---|---|---|---|
| Machine learning for suicide risk prediction in children and adolescents with electronic health records [47] | To predict suicidal behavior among children and adolescents based on their longitudinal clinical records, and determining short- and long-term risk factors | EMR data | The proposed models predicted suicidal behavior with an overall AUC > 0.80 across all prediction time windows. The model performed similarly in terms of AUC for 0- to 270-day prediction windows (AUC = 0.84–0.86). The predictive performance declines for the one-year prediction window (AUC = 0.81, 95% confidence interval [CI] 0.76–0.86) since fewer patients had clinical records 1 year before the | Machine learning | Yes | Yes |

| | | | | | | |
|---|---|---|---|---|---|---|
| | | | observation point. For all prediction windows, the model detected 53–62% of suicide cases with 90% specificity. Consistent with the low prevalence (from 0.43 to 0.95%) of suicidal behavior in the studied cohort, the PPVs across all prediction windows ranged from 3 to 6% for 90% specificity, and from 4 to 8% for 95% specificity. | | | |
| Comparing machine learning to a rule-based approach for predicting suicidal behavior among adolescents: Results from a | To predict future suicidal behavior than a simple decision rule that classifies every adolescent with history of suicide ideation at baseline as at risk (current practice) | Survey | The AUC of the Random Forest (0.79) and Lasso regression (0.76) were both higher than the AUC of the decision rule (0.64). The | Random Forest and the Lasso Regression | Yes | No |

| | | | | | | |
|---|---|---|---|---|---|---|
| longitudinal population-based survey [48] | | | Random Forest achieved slightly (but non-significantly) higher sensitivity than the decision rule (0.37 versus 0.34), with the same specificity (0.94). With Lasso Regression the sensitivity increased significantly (0.52), but at the expense of the specificity (0.85). | | | |
| Health-behaviors associated with the growing risk of adolescent suicide attempts: A data-driven cross-sectional study [49] | To Identify and examine the associations between health behaviors and increased risk of adolescent suicide attempts, while controlling for socio-economic and demographic differences. | Survey | The non-parametric Bayesian tree ensemble model outperformed all other models, with 80.0% accuracy in goodness-of-fit (F-score: 0.802) and 78.2% in predictive | Machine learning Bagging, random forest, Bayesian, Support Vector Machine (SVM) | Yes | No |

| | | | | | | |
|---|---|---|---|---|---|---|
| | | | accuracy (F-score: 0.785) | | | |
| Word usage in spontaneous speech as a predictor of depressive symptoms among youth at high risk for mood disorders [50] | To examine whether digital phenotyping of spontaneous speech, such as the use of specific word categories during speech samples, was associated with depressive symptoms in youth who were at familial and clinical risk for mood disorders. | Randomized trial data collection from therapy | Not reported. | Machine learning | No | Yes |
| Predicting suicidal thoughts and behavior among adolescents using the risk and protective factor framework: A large-scale machine learning approach [51] | To develop a prediction algorithm for Suicidal thoughts and behaviors (STB) among adolescents using the risk and protective factor framework and social determinants of health. | Survey | ROC; Naïve Bayes 0.52, Logistic regression 0.53, lightGBM 0.91, K-NN 0.84) | Machine learning (Naïve bayes, logistic regression, light GBM, K-NN) | Yes | Yes |
| Machine-Learning prediction of comorbid substance use disorders in ADHD youth using Swedish registry data [52] | To identify of at-risk youth would help allocate scarce resources for prevention programs. | National register | The area under the receiver operating characteristic curve (AUC) was 0.73(95%CI 0.70-0.76) for the random forest model | Random forest, neural network | No | Yes |

| | | | | | | |
|---|---|---|---|---|---|---|
| | | | (RF). Removing prior diagnosis from the predictors, the RF model was still able to achieve significant AUCs when predicting all SUD diagnoses (0.69, 95%CI 0.66-0.72) or new diagnoses (0.67, 95%CI: 0.64, 0.71) during age 18-19. | | | |
| A machine learning algorithm-based model for predicting the risk of non-suicidal self-injury among adolescents in western China: A multicentre cross-sectional study [53] | To develop a predictive model, aiming to more accurately assess the risk of Non-Suicidal Self-Injury (NSSI) in Chinese adolescents. | Data from schools | The Accuracy and area under the receiver operating characteristic curve (AUC) values of the XGBoost model were 0.956 and 0.870 in the training set, and 0.954 and 0.830 in the testing set, respectively. | eXtreme Gradient Boosting (XGBoost) model and multivariate logistic regression model | Yes | Yes |

| | | | | | | |
|---|---|---|---|---|---|---|
| Prediction of non-suicidal self-injury in adolescents at the family level using regression methods and machine learning [54] | To improve the accuracy of family-level risk prediction for Non-Suicidal Self-Injury (NSSI) | Survey | The AUCs of the two models, logistic regression and random forest, were 0.852 and 0.835, respectively. | Logistic regression model and Random forest model | Yes | Yes |
| Identifying the risk of depression in a large sample of adolescents: An artificial neural network based on random forest [55] | To develop an artificial neural network (ANN) prediction model incorporating random forest (RF) screening ability for predicting the risk of depression in adolescents and identifies key risk factors to provide a new approach for primary care screening of depression among adolescents. | PHQ-9 | Ten variables were included in the final prediction model with a model accuracy of 85.03%, AUC of 0.892, specificity of 89.79%, and sensitivity of 70.81%. | Artificial neural network (ANN), Random forest (RF) | No | No |
| Machine learning based identification of structural brain alterations underlying suicide risk in adolescents [56] | To examine structural brain alterations in adolescents that can discriminate individuals with suicide risk from typically developing adolescents using ML algorithms. | MRI scans from 158 adolescents. | Achieves a classification accuracy of 74.79% using support vector machine (SVM) with sensitivity of 75.90% and specificity of 74.07%. | Machine learning, including support vector machine (SVM), k-nearest neighbors (K-NN), and ensemble methods. | Yes | Yes |

| | | | | | | |
|---|---|---|---|---|---|---|
| Detecting Psychological Disorders with Stylometry: the Case of ADHD [57] | To explore the potential of stylometry and Natural Language Processing (NLP) in detecting Attention deficit hyperactivity disorder (ADHD) from autobiographical narratives of adolescents. | Autobiographical narratives collected from participants | SVM classifier accuracy of 92% in differentiating ADHD from control group narratives. | Natural Language Processing (NLP), Stylometry | Yes | Yes |
| Determining the Level of Depression using BDI-II through Voice Recognition [58] | To determine the level of depression in individuals using voice recognition analyzed through SVM and DT algorithms based on the BDI-II questionnaire. | Voice recordings of participants answering the BDI-II questionnaire. | SVM algorithm accuracy rate of 40.5%, Decision Tree algorithm accuracy rate of 28.57%. | Support Vector Machine (SVM) and Decision Tree (DT) | Yes | Yes |
| Exploring the Impact of Co-Experiencing Stressor Events for Teens Stress Forecasting. [59] | To leverage the intra-group impact of co-experiencing stressor events to supplement sparse individual stress series and improve individual stress prediction. | Microblog data analyzing stress levels and events | Uses a cluster-based NARX recurrent neural network; validated on a dataset of 124 high school students. | NARX recurrent neural network | Yes | Not applicable |
| The application of artificial intelligence technology in education influences Chinese adolescent's | To examine the impact of artificial intelligence in education on adolescents' emotional perception. | Reaction times to emotional faces and survey responses | Shows a negative effect of AI on adolescents' emotional perception. | Machine learning methods | No | Not applicable |

| | | | | | | |
|---|---|---|---|---|---|---|
| emotional perception. [60] | | | | | | |
| Analyzing and Identifying Teens' Stressful Periods and Stressor Events from a Microblog. [61] | To analyze and identify teens' stressful periods and the stressor events from their microblog postings, understanding stress from appearance to essence. | Microblog posts from students. | Stressful periods: recall 0.761, precision 0.737, F1-measure 0.734; Top-3 stressor events: recall 0.763, precision 0.756, F1-measure 0.759. | Data mining techniques and Poisson-based statistical models. | Yes | Not applicable |
| ASD-SAENet: A Sparse Autoencoder, and Deep-Neural Network Model for Detecting Autism Spectrum Disorder (ASD) Using fMRI Data [62] | To develop a deep-learning model to classify ASD from typical control subjects using fMRI data. | fMRI data from the ABIDE dataset. | Achieves an average accuracy of 70.8%, demonstrating superior specificity of 79.1%. | Sparse Autoencoder (SAE) and Deep Neural Network (DNN) | Yes | Yes |
| Bullying and psychotic symptoms in youth with bipolar disorder. [63] | Examine the association between psychosis in pediatric bipolar disorder with bullying victimization. | Bullying experiences, de mographic information, and clinical variables | Odds ratio for bullying = 7.3 (95%CI = 2–32); Machine learning model accuracy = 75%, sensitivity = 77.91%, specificity = 69.05%, AUC = 0.86. | Naïve Bayes | Yes | Not applicable |

| | | | | | | |
|---|---|---|---|---|---|---|
| Prediction by data mining of suicide attempts in Korean adolescents: a national study [64] | To develop a prediction model for suicide attempts in Korean adolescents using decision tree analysis. | Sociodemographic and psychological variables from a national dataset. | Showed a suicide attempt rate of 9.5% in the studied group, with specific impacts from factors like depression severity, delinquency, and family intimacy. | Decision tree analysis, data mining. | No | Yes |
| Using Expert System Application to Diagnose Online Game Addiction in Junior High School Students: Case Study in Five Big City in Indonesia [65] | To develop and describe the use of an expert system to diagnose online game addiction in junior high school students in Indonesia. | User, addiction, symptom, question, solution, and rule data. | Among 1000 Junior High School Students, 69% have a low-level addiction, 25% medium-level, and 6% high-level. | Expert system with certainty factor method | Yes | Not applicable |
| A novel, complex systems approach to modelling risk of psychological distress in young adolescents [66] | To model risk factors contributing to psychological distress in adolescents using Bayesian networks. | Includes factors like eating behavior, social connectedness, and physical activity. | Describes the influence of factors like unhealthy eating and low social connectedness on psychological distress without specific numerical metrics. | Bayesian networks | Yes | Yes |

| | | | | | | |
|---|---|---|---|---|---|---|
| Machine Learning Classification of First-Onset Drug-Naive MDD Using Structural MRI [67] | To classify adolescent patients with Major Depressive Disorder (MDD) from healthy controls using only structural MRI data. | Structural MRI data | Utilizes Support Vector Machine (SVM) , demonstrating potential for classification. TPR, TNR, Accuracy, Precision, F1, BCR | Support Vector Machine (SVM) and other machine learning models | Yes | Yes |
| Hippocampal subfield alterations in pediatric patients with post-traumatic stress disorder [68] | To investigate hippocampal subfield volume using an automated segmentation method and explore the subfield-centered functional connectivity aberrations related to anatomical changes in a homogeneous population of traumatized children with and without PTSD. | MRI scans for volumetric analysis | Identified significant mean volume reductions in specific hippocampal subfields, with classification accuracy of PTSD identification at 69%. | Machine learning using random forest classifiers | No | Yes |
| The Pandemic's Toll on Young Adolescents: Prevention and Intervention Targets to Preserve Their | To identify vulnerable young adolescents during the COVID-19 pandemic and provide insights for strategies to help adolescents cope better in crisis situations. | Survey data on psychosocial and lifestyle domains | Utilized longitudinal data and machine learning models to identify predictors of mental health issues, focusing | Logistic regression and gradient boosting (XGBoost) | Yes | Yes |

| | | | | | | |
|---|---|---|---|---|---|---|
| Mental Health [69] | | | on positive affect, stress, anxiety, and depressive symptoms. | | | |
| Understanding posttraumatic stress trajectories in adolescent females: A strength-based machine learning approach examining risk and protective factors including online behaviors. [70] | To investigate the trajectories of posttraumatic stress symptoms (PTSS) in adolescent females who have experienced childhood sexual abuse (CSA) or other potentially traumatic events (PTEs), focusing on psychosocial risk and protective factors, including online behaviors. | Data collected from assessments including psychological evaluations and questionnaires over three years. | Identifies four PTSS trajectories: resilient, emerging, recovering, and chronic, with the resilient trajectory encompassing 52.1% of the sample. | Latent growth mixture modeling (LGMM) and LASSO logistic regression to analyze data. | Yes | Yes |
| Classification of Adolescent Psychiatric Patients at High Risk of Suicide Using the Personality Assessment Inventory by Machine Learning [71] | To develop and validate a machine learning model to classify adolescent psychiatric patients at high risk of suicide using Personality Assessment Inventory data. | Personality Assessment Inventory data along with demographic and clinical data | Utilized various machine learning techniques like Random Forest (RF), Artificial Neural Network (ANN), support vector machine (SVM), achieving AUROCs above 0.9 with the best performance | Machine learning models including logistic regression, random forest, artificial neural networks, support vector machine, and extreme gradient boosting | Yes | Yes |

| | | | | | | |
|---|---|---|---|---|---|---|
| | | | from the RF model. | | | |
| Psychological pain and sociodemographic factors classified suicide attempt and non-suicidal self-injury in adolescents. [72] | To utilize machine learning to explore the psychological similarities and differences between suicide attempt (SA) and non-suicidal self-injury (NSSI), focusing on psychological pain. | Psychological assessments and sociodemograp hic data | AUCs: 0.61–0.87 | Multilayer Perceptron (MLP), SGD (Stochastic Gradient Descent), AdaBoost, Support Vector Classifier (SVC), Random Forest, Naïve Bayes | Yes | Yes |
| Unraveling how the adolescent brain deals with criticism using dynamic causal modeling (DCM) [73] | To explore the dynamic causal interactions within the neural network in adolescents when dealing with criticism using fMRI and DCM. | fMRI data during exposure to criticism and praise | Effective connectivity changes among brain regions | Bayesian Method | Yes | Not applicable |
| Predicting juvenile offending: A comparison of data mining methods. [74] | To compare logistic regression and data mining techniques (decision trees, artificial neural networks, and support vector machines) for predicting juvenile offending. | Data from a large sample of adolescents | Accuracy rates of 95% and above, supported by receiver operating characteristic analyses. | Decision Trees, Artificial Neural Networks, Support Vector Machines | Yes | Yes |
| Analysis of stress and academic-sports commitment through Self-organizing | To analyze the relationship between stress variables, resilience, coping, and academic and sports commitment in | Psychological and sociodemograp hic data from adolescent male athletes. | Utilizes self-organizing maps to identify clusters of athletes with similar | Self-organizing Artificial Neural Networks (SOM). | Yes | Yes |

| | | | | | | |
|---|---|---|---|---|---|---|
| Artificial Neural Networks. [75] | adolescent athletes using Self-organizing Artificial Neural Networks (SOM). | | psychological and sociodemographic characteristics. | | | |
| Age, Quality of Life, and Mental Well-Being in Adolescent Population: A Network Model Tree Analysis. [76] | To analyze the relationship between health-related quality of life (HRQoL) and mental well-being among adolescents of different age and gender groups using network model trees. | Survey data from KIDSCREEN-27 and Warwick–Edinburgh Mental Wellbeing Scale (WEMWBS). | Not reported. | Network model trees to analyze HRQoL and mental well-being. | No | Yes |
| Automated Risk Assessment for School Violence: a Pilot Study. [77] | To evaluate an automated risk assessment method for predicting school violence using machine learning based on manual annotations of interviews. | Interview transcriptions and demographic data | The novel machine learning algorithm achieved an AUC of 91.02% to 91.45% in predicting the risk of school violence. | Machine learning algorithms analyzing interview content | No | Yes |
| Psychotherapeutic Tool for Addressing Depression in Teenagers Through Video Games. [78] | To develop a video game as a psychotherapeutic tool for addressing depression in teenagers. | Game interaction and user feedback | 80% of the patients found the video game tool helpful in facilitating the healing of depression. | Video game development | Yes | Not applicable |

| | | | | | | |
|---|---|---|---|---|---|---|
| SOLWOE—A Novel Way to Diagnose Depression Among Teenagers. [79] | To diagnose depression among teenagers using a novel hybrid model incorporating questionnaires and AI-driven voice interviews. | Responses from questionnaires and voice interviews | Focuses on methodology rather than specific results. | Hybrid model including Regression and Support Vector Machine | Yes | Not applicable |
| A Virtual Conversational Agent for Teens with Autism Spectrum Disorder: Experimental Results and Design Lessons. [80] | To develop a virtual conversational agent, LISSA, that helps teens with Autism Spectrum Disorder (ASD) improve their social skills through practice and feedback. | Dialogue interactions, nonverbal cues | User engagement metrics and qualitative feedback through thematic analysis of interviews. | Machine learning models for real-time feedback and dialog management, including hidden Markov models. | Yes | Not applicable |
| Exploration of Adolescent Depression Risk Prediction Based on Census Surveys and General Life Issues. [81] | To develop predictive models for assessing depression risk in adolescents using census survey data. | National Survey of Children's Health (NSCH) data | Accuracy, Area Under the Curve (AUC) | Machine learning models including logistic regression, Support Vector Machine, and deep learning. | Yes | Not applicable |
| Tracking Representational Flexibility Development through Speech Data Mining. [82] | To evaluate the development of representational flexibility in adolescents with autism using VR-based simulation games. | Verbal utterances during VR interaction | Focuses on developing methods for real-time, in-situ performance assessment of flexibility development. | Speech data mining using machine learning for performance assessment | Yes | Not applicable |
| Evaluating a treatment selection | To evaluate a treatment selection approach for | Depressive symptoms | No significant difference in | Machine learning techniques used | Yes | Not applicable |

| | | | | | | |
|---|---|---|---|---|---|---|
| approach for online single-session interventions for adolescent depression. [83] | single-session interventions for adolescent depression, using a Personalized Advantage Index (PAI). | scores and additional psychological and demographic data | depression outcomes between matched and non-matched interventions; weak predictive relationship between expected and observed RTI. | for treatment prediction and selection | | |
| Predicting Lifetime Suicide Attempts in a Community Sample of Adolescents Using Machine Learning Algorithms. Assessment [84] | To predict lifetime suicide attempts among adolescents using machine learning models and a variety of reported variables. | Variables collected from a community sample regarding mental health, behavior, and demographics. | Achieved balanced accuracies of .76 using data 3 years prior and .85 using contemporaneou s data. | Machine learning algorithms including logistic regressions, elastic net regressions, and gradient boosting machines. | Yes | Not applicable |
| Multimodal Web Application to Infer Emotional Intelligence of Adolescent Counsellor.[85] | To develop a scalable web application that uses multimodal data to assess the emotional intelligence of adolescent counsellors. | Includes resume analysis, Multiple Choice Question (MCQ) responses, and audio responses. | Evaluation includes comparison of AI-generated results with expert ratings, demonstrating the effectiveness of multimodal assessments in identifying suitable counsellors. | Utilizes multimodal analysis including text, audio, and machine learning techniques to assess candidates. | Yes | Not applicable |

| | | | | | | |
|---|---|---|---|---|---|---|
| Designing a Clinical Decision Support System for Recommending Computerized Cognitive Rehabilitation Programs: the Experience of Attention Deficit Hyperactivity Disorder. [86] | To design a clinical decision support system that proposes suitable computerized cognitive rehabilitation programs for children and adolescents with Attention Deficit Hyperactivity Disorder (ADHD). | Data includes cognitive test results and expert recommendations. | Effectiveness of the system is mentioned in terms of its ability to prescribe appropriate rehabilitation programs based on individual deficits. | The system uses rule-based algorithms to recommend specific cognitive rehabilitation programs. | Yes | Not applicable |
| Predicting Academic Success of Autistic Students in Higher Education [87] | To develop predictive models for the academic success of autistic bachelor students and compare these models against those of students with other health conditions and those without any conditions. | Administrative and academic performance data from the university's student information system | Predictive accuracy more precise for autistic students compared to non-autistic peers | Machine learning models, propensity score weighting | Yes | Not applicable |
| Neurocognitive Risk Phenotyping to Predict Mood Symptoms in Adolescence. [88] | To predict mood disorders in adolescence using neurocognitive variables to identify neurocognitive predictors of symptom expression and clinical profiles. | Neurocognitive data including reward processing and executive functioning | Not reported. | Machine learning models including penalized mixed-effects models | Yes | Yes |

*Criteria to evaluate the performance of Al models:

1. Accuracy: A model's measurement's proximity to the standard or real value, calculated as the number of correct predictions divided by the total number of predictions.
2. F1 Score: The weighted average of precision and recall.
3. Sensitivity: The metric that assesses a model's ability to predict true positives in each accessible category.
4. Specificity: The metric that assesses a model's ability to predict true negatives in each accessible category.
5. Precision or Positive Predictive Value (PPV): The proportion of cases labelled as positive that was genuinely positive.
6. Area Under Curve (AUC): The probability that a classifier will rank a randomly chosen positive instance higher than a randomly chosen negative example.
7. Negative Predictive Value (NPV): The proportions of positive and negative results in statistics and diagnostic tests that are true positive and true negative results.
8. Confidence Interval (CI): The probability that a parameter will fall between a pair of values around the mean.
9. Recall: The measure with which the model recognizes True Positives.

References

1. Khaleghi A, Sheikhani A, Mohammadi MR, Nasrabadi AM, Vand SR, Zarafshan H, Moeini M. EEG classification of adolescents with type I and type II of bipolar disorder. Australas Phys Eng Sci Med. 2015 Dec;38(4):551-9. doi: 10.1007/s13246-015-0375-0. PMID: 26472650.
2. Bekele, E., Zheng, Z., Swanson, A., Davidson, J., Warren, Z., Sarkar, N. (2013). Virtual Reality-Based Facial Expressions Understanding for Teenagers with Autism . In: Stephanidis, C., Antona, M. (eds) Universal Access in Human-Computer Interaction. User and Context Diversity. UAHCI 2013. Lecture Notes in Computer Science, vol 8010. Springer, Berlin, Heidelberg. https://doi.org/10.1007/978-3-642-39191-0_50
3. Jin, L., Xue, Y., Li, Q., Feng, L. (2016). Integrating Human Mobility and Social Media for Adolescent Psychological Stress Detection. In: Navathe, S., Wu, W., Shekhar, S., Du, X., Wang, S., Xiong, H. (eds) Database Systems for Advanced

Applications. DASFAA 2016. Lecture Notes in Computer Science(), vol 9643. Springer, Cham. https://doi.org/10.1007/978-3-319-32049-6_23

4. Y. -y. Gan.(2012)) Evaluation on life satisfaction of left-behind junior high school children based on LVQ network, 8th International Conference on Natural Computation, Chongqing, China, 2012, pp. 405-408, doi: 10.1109/ICNC.2012.6234755.
5. S. V. Tyulyupo, A. A. Andrakhanov, B. A. Dashieva and A. V. Tyryshkin, "Adolescents Psychological Well-Being Estimation Based on a Data Mining Algorithm," 2018 IEEE 13th International Scientific and Technical Conference on Computer Sciences and Information Technologies (CSIT), Lviv, Ukraine, 2018, pp. 475-478, doi: 10.1109/STC-CSIT.2018.8526628.
6. Xue, Y., Li, Q., Jin, L., Feng, L., Clifton, D.A., Clifford, G.D. (2014). Detecting Adolescent Psychological Pressures from Micro-Blog. In: Zhang, Y., Yao, G., He, J., Wang, L., Smalheiser, N.R., Yin, X. (eds) Health Information Science. HIS 2014. Lecture Notes in Computer Science, vol 8423. Springer, Cham. https://doi.org/10.1007/978-3-319-06269-3_10
7. Strigo IA, Murray SB, Simmons AN, Bernard RS, Huang JS, Kaye WH. The clinical application of fMRI data in a single-patient diagnostic conundrum: Classifying brain response to experimental pain to distinguish between gastrointestinal, depressive and eating disorder symptoms. J Clin Neurosci. 2017 Nov;45:149-153. doi: 10.1016/j.jocn.2017.07.023. Epub 2017 Aug 16. PMID: 28823587.
8. Zhang Z, Liao M, Yao Z, Hu B, Xie Y, Zheng W, Hu T, Zhao Y, Yang F, Zhang Y, Su L, Li L, Gutknecht J, Majoe D. Frequency-Specific Functional Connectivity Density as an Effective Biomarker for Adolescent Generalized Anxiety Disorder. Front Hum Neurosci. 2017 Dec 5;11:549. doi: 10.3389/fnhum.2017.00549. PMID: 29259549; PMCID: PMC5723402.

9. Chen H, Duan X, Liu F, Lu F, Ma X, Zhang Y, Uddin LQ, Chen H. Multivariate classification of autism spectrum disorder using frequency-specific resting-state functional connectivity--A multi-center study. Prog Neuropsychopharmacol Biol Psychiatry. 2016 Jan 4;64:1-9. doi: 10.1016/j.pnpbp.2015.06.014. Epub 2015 Jul 4. PMID: 26148789.
10. Thakur, S. (2016). Identification of Chief Characteristics of Alcohol Consumption Traits in Schools Using Rough Set and Formal Concept Analysis.
11. Foland-Ross LC, Sacchet MD, Prasad G, Gilbert B, Thompson PM, Gotlib IH. Cortical thickness predicts the first onset of major depression in adolescence. Int J Dev Neurosci. 2015 Nov;46:125-31. doi: 10.1016/j.ijdevneu.2015.07.007. Epub 2015 Aug 24. PMID: 26315399; PMCID: PMC4604750.
12. Hart H, Chantiluke K, Cubillo AI, Smith AB, Simmons A, Brammer MJ, Marquand AF, Rubia K. Pattern classification of response inhibition in ADHD: toward the development of neurobiological markers for ADHD. Hum Brain Mapp. 2014 Jul;35(7):3083-94. doi: 10.1002/hbm.22386. Epub 2013 Oct 11. PMID: 24123508; PMCID: PMC4190683.
13. Zhou Y, Yu F, Duong T. Multiparametric MRI characterization and prediction in autism spectrum disorder using graph theory and machine learning. PLoS One. 2014 Jun 12;9(6):e90405. doi: 10.1371/journal.pone.0090405. PMID: 24922325; PMCID: PMC4055499.
14. Mullick, T., Radovic, A., Shaaban, S., & Doryab, A. (2022). Predicting depression in adolescents using mobile and wearable sensors: Multimodal machine learning–based exploratory study. JMIR Formative Research, 6(6), e35807. https://doi.org/10.2196/35807

15. Gervilla E, Cajal B, Palmer A. Quantification of the influence of friends and antisocial behaviour in adolescent consumption of cannabis using the ZINB model and data mining. Addict Behav. 2011 Apr;36(4):368-74. doi: 10.1016/j.addbeh.2010.12.007. Epub 2010 Dec 10. PMID: 21190799.

16. García EG, Blasco BC, López RJ, Pol AP. Study of the factors associated with substance use in adolescence using Association Rules. Adicciones. 2010;22(4):293-9. PMID: 21152847.

17. Kashani JH, Nair SS, Rao VG, Nair J, Reid JC. Relationship of personality, environmental, and DICA variables to adolescent hopelessness: a neural network sensitivity approach. J Am Acad Child Adolesc Psychiatry. 1996 May;35(5):640-5. doi: 10.1097/00004583-199605000-00019. PMID: 8935211.

18. Reid JC, Nair SS, Kashani JH, Rao VG. Detecting dysfunctional behavior in adolescents: the examination of relationships using neural networks. Proc Annu Symp Comput Appl Med Care. 1994:743-6. PMID: 7950023; PMCID: PMC2247956.

19. Y. Li, J. Huang, H. Wang and L. Feng, "Predicting Teenager's Future Stress Level from Micro-Blog," 2015 IEEE 28th International Symposium on Computer-Based Medical Systems, Sao Carlos, Brazil, 2015, pp. 208-213, doi: 10.1109/CBMS.2015.25.

20. DiGuiseppi GT, Davis JP, Leightley D, Rice E. Predictors of Adolescents' First Episode of Homelessness Following Substance Use Treatment. J Adolesc Health. 2020 Apr;66(4):408-415. doi: 10.1016/j.jadohealth.2019.11.312. Epub 2020 Feb 10. PMID: 32057607.

21. Ruan H, Zhou Y, Luo Q, Robert GH, Desrivières S, Quinlan EB, Liu Z, Banaschewski T, Bokde ALW, Bromberg U, Büchel C, Flor H, Frouin V, Garavan H, Gowland P, Heinz A, Ittermann B, Martinot JL, Martinot MP, Nees F, Orfanos DP, Poustka L, Hohmann S, Fröhner JH, Smolka MN, Walter H, Whelan R, Li F, Schumann G, Feng J; IMAGEN Consortium. Adolescent binge drinking disrupts normal trajectories of brain functional organization and personality maturation. Neuroimage Clin. 2019;22:101804. doi: 10.1016/j.nicl.2019.101804. Epub 2019 Mar 31. PMID: 30991616; PMCID: PMC6451196.
22. Velupillai S, Epstein S, Bittar A, Stephenson T, Dutta R, Downs J. Identifying Suicidal Adolescents from Mental Health Records Using Natural Language Processing. Stud Health Technol Inform. 2019 Aug 21;264:413-417. doi: 10.3233/SHTI190254. PMID: 31437956.
23. Downs J, Dean H, Lechler S, Sears N, Patel R, Shetty H, Hotopf M, Ford T, Kyriakopoulos M, Diaz-Caneja CM, Arango C, MacCabe JH, Hayes RD, Pina-Camacho L. Negative Symptoms in Early-Onset Psychosis and Their Association With Antipsychotic Treatment Failure. Schizophr Bull. 2019 Jan 1;45(1):69-79. doi: 10.1093/schbul/sbx197. PMID: 29370404; PMCID: PMC6293208.
24. Fitzgerald, A., Mac Giollabhui, N., Dolphin, L., Whelan, R., & Dooley, B. (2018). Dissociable psychosocial profiles of adolescent substance users. PLOS ONE, 13(8), e0202498. https://doi.org/10.1371/journal.pone.0202498
25. Fujisawa TX, Shimada K, Takiguchi S, Mizushima S, Kosaka H, Teicher MH, Tomoda A. Type and timing of childhood maltreatment and reduced visual cortex volume in children and adolescents with reactive attachment disorder. Neuroimage Clin. 2018 Jul 23;20:216-221. doi: 10.1016/j.nicl.2018.07.018. PMID: 30094171; PMCID: PMC6080635.

26. Liu Y, Guo W, Zhang Y, Lv L, Hu F, Wu R, Zhao J. Decreased Resting-State Interhemispheric Functional Connectivity Correlated with Neurocognitive Deficits in Drug-Naive First-Episode Adolescent-Onset Schizophrenia. Int J Neuropsychopharmacol. 2018 Jan 1;21(1):33-41. doi: 10.1093/ijnp/pyx095. PMID: 29228204; PMCID: PMC5795351.
27. Lenhard F, Sauer S, Andersson E, Månsson KN, Mataix-Cols D, Rück C, Serlachius E. Prediction of outcome in internet-delivered cognitive behaviour therapy for paediatric obsessive-compulsive disorder: A machine learning approach. Int J Methods Psychiatr Res. 2018 Mar;27(1):e1576. doi: 10.1002/mpr.1576. Epub 2017 Jul 28. PMID: 28752937; PMCID: PMC6877165.
28. Geraci J, Wilansky P, de Luca V, Roy A, Kennedy JL, Strauss J. Applying deep neural networks to unstructured text notes in electronic medical records for phenotyping youth depression. Evid Based Ment Health. 2017 Aug;20(3):83-87. doi: 10.1136/eb-2017-102688. Epub 2017 Jul 24. PMID: 28739578; PMCID: PMC5566092.
29. Squeglia LM, Ball TM, Jacobus J, Brumback T, McKenna BS, Nguyen-Louie TT, Sorg SF, Paulus MP, Tapert SF. Neural Predictors of Initiating Alcohol Use During Adolescence. Am J Psychiatry. 2017 Feb 1;174(2):172-185. doi: 10.1176/appi.ajp.2016.15121587. Epub 2016 Aug 19. Erratum in: Am J Psychiatry. 2017 Jan 1;174(1):80. doi: 10.1176/appi.ajp.2016.1741correction. PMID: 27539487; PMCID: PMC5288131.
30. Lim JS, Yang CM, Baek JW, Lee SY, Kim BN. Prediction Models for Suicide Attempts among Adolescents Using Machine Learning Techniques. Clin Psychopharmacol Neurosci. 2022 Nov 30;20(4):609-620. doi: 10.9758/cpn.2022.20.4.609. PMID: 36263637; PMCID: PMC9606439.

31. Lorge, I., Joyce, D. W., & Kormilitzin, A. (2024). Large Language Models Perform on Par with Experts Identifying Mental Health Factors in Adolescent Online Forums. arXiv preprint arXiv:2404.16461.

32. Lv J, Guo X, Meng C, Fei J, Ren H, Zhang Y, Qin Z, Hu Y, Yuan T, Liang L, Li C, Yue J, Gao R, Song Q, Zhao X, Mei S. The cross-sectional study of depressive symptoms and associated factors among adolescents by backpropagation neural network. Public Health. 2022 Jul;208:52-58. doi: 10.1016/j.puhe.2022.04.017. Epub 2022 Jun 7. PMID: 35687956.

33. McCoy, T. H., & Perlis, R. H. (2024). Dimensional measures of psychopathology in children and adolescents using large language models. Biological Psychiatry, 96(12), 940–947. https://doi.org/10.1016/j.biopsych.2024.05.008

34. McIsaac MA, Reaume M, Phillips SP, Michaelson V, Steeves V, Davison CM, Vafaei A, King N, Pickett W. A novel application of a data mining technique to study intersections in the social determinants of mental health among young Canadians. SSM Popul Health. 2021 Oct 21;16:100946. doi: 10.1016/j.ssmph.2021.100946. PMID: 34746359; PMCID: PMC8551646.

35. Mouchabac S, Leray P, Adrien V, Gollier-Briant F, Bonnot O. Prevention of Suicidal Relapses in Adolescents With a Smartphone Application: Bayesian Network Analysis of a Preclinical Trial Using In Silico Patient Simulations. J Med Internet Res. 2021 Sep 30;23(9):e24560. doi: 10.2196/24560. PMID: 34591030; PMCID: PMC8517816.

36. Nagaoka D, Uno A, Usami S, Tanaka R, Minami R, Sawai Y, Okuma A, Yamasaki S, Miyashita M, Nishida A, Kasai K, Ando S. Identify adolescents' help-seeking intention on suicide through self- and caregiver's assessments of psychobehavioral

problems: deep clustering of the Tokyo TEEN Cohort study. Lancet Reg Health West Pac. 2023 Dec 13;43:100979. doi: 10.1016/j.lanwpc.2023.100979. PMID: 38456092; PMCID: PMC10920037.

37. Penfold RB, Johnson E, Shortreed SM, Ziebell RA, Lynch FL, Clarke GN, Coleman KJ, Waitzfelder BE, Beck AL, Rossom RC, Ahmedani BK, Simon GE. Predicting suicide attempts and suicide deaths among adolescents following outpatient visits. J Affect Disord. 2021 Nov 1;294:39-47. doi: 10.1016/j.jad.2021.06.057. Epub 2021 Jul 1. PMID: 34265670; PMCID: PMC8820270.

38. Qasrawi R, Vicuna Polo SP, Abu Al-Halawa D, Hallaq S, Abdeen Z. Assessment and Prediction of Depression and Anxiety Risk Factors in Schoolchildren: Machine Learning Techniques Performance Analysis. JMIR Form Res. 2022 Aug 31;6(8):e32736. doi: 10.2196/32736. PMID: 35665695; PMCID: PMC9475423.

39. Qasrawi R, Vicuna Polo S, Abu Khader R, Abu Al-Halawa D, Hallaq S, Abu Halaweh N, Abdeen Z. Machine learning techniques for identifying mental health risk factor associated with schoolchildren cognitive ability living in politically violent environments. Front Psychiatry. 2023 May 26;14:1071622. doi: 10.3389/fpsyt.2023.1071622. PMID: 37304448; PMCID: PMC10250653.

40. Rajapaksha RMDS, Filbey F, Biswas S, Choudhary P. A Bayesian learning model to predict the risk for cannabis use disorder. Drug Alcohol Depend. 2022 Jul 1;236:109476. doi: 10.1016/j.drugalcdep.2022.109476. Epub 2022 Apr 29. PMID: 35588608.

41. Ren Z, Xin Y, Wang Z, Liu D, Ho RCM, Ho CSH. What Factors Are Most Closely Associated With Mood Disorders in Adolescents During the COVID-19 Pandemic? A Cross-Sectional Study Based on 1,771 Adolescents in Shandong Province, China. Front Psychiatry. 2021 Sep 16;12:728278. doi: 10.3389/fpsyt.2021.728278. PMID: 34603106; PMCID: PMC8481827.
42. Rothenberg WA, Bizzego A, Esposito G, Lansford JE, Al-Hassan SM, Bacchini D, Bornstein MH, Chang L, Deater-Deckard K, Di Giunta L, Dodge KA, Gurdal S, Liu Q, Long Q, Oburu P, Pastorelli C, Skinner AT, Sorbring E, Tapanya S, Steinberg L, Tirado LMU, Yotanyamaneewong S, Alampay LP. Predicting Adolescent Mental Health Outcomes Across Cultures: A Machine Learning Approach. J Youth Adolesc. 2023 Aug;52(8):1595-1619. doi: 10.1007/s10964-023-01767-w. Epub 2023 Apr 19. PMID: 37074622; PMCID: PMC10113992.
43. Santoso, M.S., Suryadi, J.J., Marchellino, K., Nabiilah, G.Z., & Rojali (2023). A Comparative Analysis of Decision Tree and Support Vector Machine on Suicide Ideation Detection. International Conference on Computer Science and Computational Intelligence.
44. Sedgwick R, Bittar A, Kalsi H, Barack T, Downs J, Dutta R. Investigating online activity in UK adolescent mental health patients: a feasibility study using a natural language processing approach for electronic health records. BMJ Open. 2023 May 25;13(5):e061640. doi: 10.1136/bmjopen-2022-061640. PMID: 37230520; PMCID: PMC10230886.
45. Song J, Yang J, Yoo S, Cheon K, Yun S, Shin Y. Exploring Korean adolescent stress on social media: a semantic network analysis. PeerJ. 2023 Mar 24;11:e15076. doi: 10.7717/peerj.15076. PMID: 36992939; PMCID: PMC10042152.

46. Stevens HR, Acic I, Rhea S. Natural Language Processing Insight into LGBTQ+ Youth Mental Health During the COVID-19 Pandemic: Longitudinal Content Analysis of Anxiety-Provoking Topics and Trends in Emotion in LGBTeens Microcommunity Subreddit. JMIR Public Health Surveill. 2021 Aug 17;7(8):e29029. doi: 10.2196/29029. PMID: 34402803; PMCID: PMC8372845.

47. Su, C., Aseltine, R., Doshi, R. et al. Machine learning for suicide risk prediction in children and adolescents with electronic health records. Transl Psychiatry 10, 413 (2020). https://doi.org/10.1038/s41398-020-01100-0

48. van Vuuren CL, van Mens K, de Beurs D, Lokkerbol J, van der Wal MF, Cuijpers P, Chinapaw MJM. Comparing machine learning to a rule-based approach for predicting suicidal behavior among adolescents: Results from a longitudinal population-based survey. J Affect Disord. 2021 Dec 1;295:1415-1420. doi: 10.1016/j.jad.2021.09.018. Epub 2021 Sep 17. PMID: 34620490.

49. Wei Z, Mukherjee S. Health-Behaviors Associated With the Growing Risk of Adolescent Suicide Attempts: A Data-Driven Cross-Sectional Study. Am J Health Promot. 2021 Jun;35(5):688-693. doi: 10.1177/0890117120977378. Epub 2020 Dec 10. PMID: 33297721.

50. Weintraub MJ, Posta F, Ichinose MC, Arevian AC, Miklowitz DJ. Word usage in spontaneous speech as a predictor of depressive symptoms among youth at high risk for mood disorders. J Affect Disord. 2023 Feb 15;323:675-678. doi: 10.1016/j.jad.2022.12.047. Epub 2022 Dec 14. PMID: 36528134; PMCID: PMC9848879.

51. Weller O, Sagers L, Hanson C, Barnes M, Snell Q, Tass ES. Predicting suicidal thoughts and behavior among adolescents using the risk and protective factor framework: A large-scale machine learning approach. PLoS One. 2021 Nov 3;16(11):e0258535. doi: 10.1371/journal.pone.0258535. PMID: 34731169; PMCID: PMC8565727.

52. Zhang-James Y, Chen Q, Kuja-Halkola R, Lichtenstein P, Larsson H, Faraone SV. Machine-Learning prediction of comorbid substance use disorders in ADHD youth using Swedish registry data. J Child Psychol Psychiatry. 2020 Dec;61(12):1370-1379. doi: 10.1111/jcpp.13226. Epub 2020 Apr 1. PMID: 32237241; PMCID: PMC7754321.

53. Zhong Y, He J, Luo J, Zhao J, Cen Y, Song Y, Wu Y, Lin C, Pan L, Luo J. A machine learning algorithm-based model for predicting the risk of non-suicidal self-injury among adolescents in western China: A multicentre cross-sectional study. J Affect Disord. 2024 Jan 15;345:369-377. doi: 10.1016/j.jad.2023.10.110. Epub 2023 Oct 26. PMID: 37898476.

54. Zhou SC, Zhou Z, Tang Q, Yu P, Zou H, Liu Q, Wang XQ, Jiang J, Zhou Y, Liu L, Yang BX, Luo D. Prediction of non-suicidal self-injury in adolescents at the family level using regression methods and machine learning. J Affect Disord. 2024 May 1;352:67-75. doi: 10.1016/j.jad.2024.02.039. Epub 2024 Feb 13. PMID: 38360362.

55. Zhou Y, Zhang X, Gong J, Wang T, Gong L, Li K, Wang Y. Identifying the risk of depression in a large sample of adolescents: An artificial neural network based on random forest. J Adolesc. 2024 Oct;96(7):1485-1497. doi: 10.1002/jad.12357. Epub 2024 Jun 4. PMID: 38837218.

56. Bajaj, S., Blair, K.S., Dobbertin, M. et al. Machine learning based identification of structural brain alterations underlying suicide risk in adolescents. Discov Ment Health 3, 6 (2023). https://doi.org/10.1007/s44192-023-00033-6

57. Barrios, J., Gabay, S., Cafiero, F., & Debbané, M. (2023, October 17). Detecting Psychological Disorders with Stylometry: the Case of ADHD in Adolescent Autobiographical Narratives. https://doi.org/10.31234/osf.io/s5cm3

58. J. B. Balano, V. L. Huerto, S. Sanchez, A. Saharkhiz and J. D. Goma, "Determining the Level of Depression using BDI-II through Voice Recognition," 2019 IEEE 6th International Conference on Industrial Engineering and Applications (ICIEA), Tokyo, Japan, 2019, pp. 387-391, doi: 10.1109/IEA.2019.8715187.

59. Li, Q., Zhao, L., Xue, Y., Jin, L., Feng, L. (2017). Exploring the Impact of Co-Experiencing Stressor Events for Teens Stress Forecasting. In: Bouguettaya, A., et al. Web Information Systems Engineering – WISE 2017. WISE 2017. Lecture Notes in Computer Science(), vol 10570. Springer, Cham. https://doi.org/10.1007/978-3-319-68786-5_25

60. Lai T, Zeng X, Xu B, Xie C, Liu Y, Wang Z, Lu H, Fu S. The application of artificial intelligence technology in education influences Chinese adolescent's emotional perception. Curr Psychol. 2023 May 11:1-9. doi: 10.1007/s12144-023-04727-6. Epub ahead of print. PMID: 37359676; PMCID: PMC10173215.

61. Li Q, Xue Y, Zhao L, Jia J, Feng L. Analyzing and Identifying Teens' Stressful Periods and Stressor Events From a Microblog. IEEE J Biomed Health Inform. 2017 Sep;21(5):1434-1448. doi: 10.1109/JBHI.2016.2586519. Epub 2016 Jun 30. PMID: 27390193.

62. Almuqhim F, Saeed F. ASD-SAENet: A Sparse Autoencoder, and Deep-Neural Network Model for Detecting Autism Spectrum Disorder (ASD) Using fMRI Data. Front Comput Neurosci. 2021 Apr 8;15:654315. doi: 10.3389/fncom.2021.654315. PMID: 33897398; PMCID: PMC8060560.

63. Acosta JR, Librenza-Garcia D, Watts D, Francisco AP, Zórtea F, Raffa B, Kohmann A, Mugnol FE, Motta GL, Tramontina S, Passos IC. Bullying and psychotic symptoms in youth with bipolar disorder. J Affect Disord. 2020 Mar 15;265:603-610. doi: 10.1016/j.jad.2019.11.101. Epub 2019 Nov 22. PMID: 31787423.

64. Bae SM, Lee SA, Lee SH. Prediction by data mining, of suicide attempts in Korean adolescents: a national study. Neuropsychiatr Dis Treat. 2015 Sep 16;11:2367-75. doi: 10.2147/NDT.S91111. PMID: 26396521; PMCID: PMC4577255.

65. Aziz, A., Setyawan, B.W., Saddhono, K. (2021). Using expert system application to diagnose online game addiction in junior high school students: Case study in five big city in Indonesia. Ingénierie des Systèmes d'Information, Vol. 26, No. 5, pp. 445-452. https://doi.org/10.18280/isi.260503

66. Beaudequin, D., Schwenn, P., McLoughlin, L.T. et al. A novel, complex systems approach to modelling risk of psychological distress in young adolescents. Sci Rep 11, 9428 (2021). https://doi.org/10.1038/s41598-021-88932-y

67. D. Kim et al., "Machine Learning Classification of First-Onset Drug-Naive MDD Using Structural MRI," in IEEE Access, vol. 7, pp. 153977-153985, 2019, doi: 10.1109/ACCESS.2019.2949128.

68. Li L, Pan N, Zhang L, Lui S, Huang X, Xu X, Wang S, Lei D, Li L, Kemp GJ, Gong Q. Hippocampal subfield alterations in pediatric patients with post-traumatic stress disorder. Soc Cogn Affect Neurosci. 2021 Mar 5;16(3):334-344. doi: 10.1093/scan/nsaa162. PMID: 33315100; PMCID: PMC7943370.

69. Kiss O, Alzueta E, Yuksel D, Pohl KM, de Zambotti M, Műller-Oehring EM, Prouty D, Durley I, Pelham WE 3rd, McCabe CJ, Gonzalez MR, Brown SA, Wade NE, Marshall AT, Sowell ER, Breslin FJ, Lisdahl KM, Dick AS, Sheth CS, McCandliss

BD, Guillaume M, Van Rinsveld AM, Dowling GJ, Tapert SF, Baker FC. The Pandemic's Toll on Young Adolescents: Prevention and Intervention Targets to Preserve Their Mental Health. J Adolesc Health. 2022 Mar;70(3):387-395. doi: 10.1016/j.jadohealth.2021.11.023. Epub 2022 Jan 26. PMID: 35090817; PMCID: PMC8789404.

70. Haag AC, Bonanno GA, Chen S, Herd T, Strong-Jones S, S S, Noll JG. Understanding posttraumatic stress trajectories in adolescent females: A strength-based machine learning approach examining risk and protective factors including online behaviors. Dev Psychopathol. 2023 Oct;35(4):1794-1807. doi: 10.1017/S0954579422000475. Epub 2022 May 30. PMID: 35635211; PMCID: PMC9708933.
71. Kim KW, Lim JS, Yang CM, Jang SH, Lee SY. Classification of Adolescent Psychiatric Patients at High Risk of Suicide Using the Personality Assessment Inventory by Machine Learning. Psychiatry Investig. 2021 Nov;18(11):1137-1143. doi: 10.30773/pi.2021.0191. Epub 2021 Nov 5. PMID: 34732031; PMCID: PMC8600215.
72. Bao J, Wan J, Li H, Sun F. Psychological pain and sociodemographic factors classified suicide attempt and non-suicidal self-injury in adolescents. Acta Psychol (Amst). 2024 Jun;246:104271. doi: 10.1016/j.actpsy.2024.104271. Epub 2024 Apr 16. PMID: 38631150.
73. Chen Q, Bonduelle SLB, Wu GR, Vanderhasselt MA, De Raedt R, Baeken C. Unraveling how the adolescent brain deals with criticism using dynamic causal modeling. Neuroimage. 2024 Feb 1;286:120510. doi: 10.1016/j.neuroimage.2024.120510. Epub 2024 Jan 5. PMID: 38184159.

74. Ang RP, Goh DH. Predicting juvenile offending: a comparison of data mining methods. Int J Offender Ther Comp Criminol. 2013 Feb;57(2):191-207. doi: 10.1177/0306624X11431132. Epub 2011 Dec 12. PMID: 22158911.
75. Bellod, H. C., Ramón, V. B., Fernández, E. C., & Luján, J. F. G. (2021). Analysis of stress and academic-sports commitment through Self-organizing Artificial Neural Networks. Retos: nuevas tendencias en educación física, deporte y recreación, (42), 136-144.
76. Cilar Budler, L., Stiglic, G. Age, quality of life and mental well-being in adolescent population: a network model tree analysis. Sci Rep 13, 17667 (2023). https://doi.org/10.1038/s41598-023-44493-w
77. Barzman D, Ni Y, Griffey M, Bachtel A, Lin K, Jackson H, Sorter M, DelBello M. Automated Risk Assessment for School Violence: a Pilot Study. Psychiatr Q. 2018 Dec;89(4):817-828. doi: 10.1007/s11126-018-9581-8. PMID: 29713946.
78. Arogundade, O. et al. (2022). Psychotherapeutic Tool for Addressing Depression in Teenagers Through Video Games. In: Abraham, A., et al. Hybrid Intelligent Systems. HIS 2021. Lecture Notes in Networks and Systems, vol 420. Springer, Cham. https://doi.org/10.1007/978-3-030-96305-7_45
79. Anandkumar, K. M., Srinivas, V. A., Jayasurya, J., & Lakshman, K. R. (2023, February). SOLWOE—A Novel Way to Diagnose Depression Among Teenagers. In International Conference On Innovative Computing And Communication (pp. 589-600). Singapore: Springer Nature Singapore.
80. Mohammad Rafayet Ali, Seyedeh Zahra Razavi, Raina Langevin, Abdullah Al Mamun, Benjamin Kane, Reza Rawassizadeh, Lenhart K. Schubert, and Ehsan Hoque. 2020. A Virtual Conversational Agent for Teens with Autism Spectrum Disorder:

Experimental Results and Design Lessons. In Proceedings of the 20th ACM International Conference on Intelligent Virtual Agents (IVA '20). Association for Computing Machinery, New York, NY, USA, Article 2, 1–8. https://doi.org/10.1145/3383652.3423900

81. Li, Q., Wu, Y., Xu, Z., & Zhou, H. (2024). Exploration of Adolescent Depression Risk Prediction Based on Census Surveys and General Life Issues. arXiv preprint arXiv:2401.03171.
82. F. Ke, J. Moon and Z. Sokolikj, "Tracking Representational Flexibility Development through Speech Data Mining," 2020 IEEE Frontiers in Education Conference (FIE), Uppsala, Sweden, 2020, pp. 1-4, doi: 10.1109/FIE44824.2020.9273818.
83. Ahuvia IL, Mullarkey MC, Sung JY, Fox KR, Schleider JL. Evaluating a treatment selection approach for online single-session interventions for adolescent depression. J Child Psychol Psychiatry. 2023 Dec;64(12):1679-1688. doi: 10.1111/jcpp.13822. Epub 2023 May 14. PMID: 37183368.
84. Jankowsky K, Steger D, Schroeders U. Predicting Lifetime Suicide Attempts in a Community Sample of Adolescents Using Machine Learning Algorithms. Assessment. 2024 Apr;31(3):557-573. doi: 10.1177/10731911231167490. Epub 2023 Apr 24. PMID: 37092544; PMCID: PMC10903120.
85. P. Agarwal et al., "Multimodal Web Application to Infer Emotional Intelligence of Adolescent Counsellor," 2019 Grace Hopper Celebration India (GHCI), Bangalore, India, 2019, pp. 1-5, doi: 10.1109/GHCI47972.2019.9071881.
86. A. Bashiri et al., "Designing a Clinical Decision Support System for Recommending Computerized Cognitive Rehabilitation Programs: the Experience of Attention Deficit Hyperactivity Disorder," 2018 2nd National and 1st International Digital Games

Research Conference: Trends, Technologies, and Applications (DGRC), Tehran, Iran, 2018, pp. 34-39, doi: 10.1109/DGRC.2018.8712064.

87. Bakker T, Krabbendam L, Bhulai S, Meeter M, Begeer S. Predicting academic success of autistic students in higher education. Autism. 2023 Aug;27(6):1803-1816. doi: 10.1177/13623613221146439. Epub 2023 Jan 5. PMID: 36602222; PMCID: PMC10374996.

88. Kaiser RH, Moser AD, Neilson C, Jones J, Peterson EC, Ruzic L, Rosenberg BM, Hough CM, Sandman C, Schneck CD, Miklowitz DJ. Neurocognitive risk phenotyping to predict mood symptoms in adolescence. J Psychopathol Clin Sci. 2024 Jan;133(1):90-102. doi: 10.1037/abn0000866. Epub 2023 Dec 7. PMID: 38059934; PMCID: PMC10752243.
