# Supplementary material for: Use of Artificial Intelligence in Adolescents’ Mental Health Care: Systematic Scoping Review of Current Applications and Future Directions
Source: JMIR Ment Health. 2025 Jun 6;12:e70438. doi: 10.2196/70438 (PMC12165596; doi:10.2196/70438)
Supplement: Multimedia Appendix 3 [file mental-v12-e70438-s003.pdf]

Multimedia Appendix 3: AI application on the continuum of adolescents' mental healthcare

Authors' name	Illness/disease	Mediation of Diagnostic Process	Treatment	Monitoring and evaluation	Prognosis
Bekele E. et al., 2013 [1]	Autism Spectrum Disorder	Helps to identify certain emotions and differences in how processing emotional faces		Helps to improve monitoring communicatio n skills	
Zhou Y. et al., 2014 [2]	Autism Spectrum Disorder			Helps to better monitor its related biomarkers	Helps prognosis using its related biomarkers
Chen H. et al., 2016 [3]	Autism Spectrum Disorder	e-related biomarkers			
Hart H. et al., 2014 [4]	Attention Deficit Hyperactivity Disorder	Helps to improve the diagnostic accuracy and optimally clinical outcomes			
Khaleghi A. et al., 2015 [5]	Bipolar disorders type 1 and 2	Helps to differentiate between two subtypes			
Ang RP. et al., 2013 [6]	Adolescents' offending and delinquency	Helps with diagnosis		Helps to improve the follow-ups	
Strigo IA. et al., 2017 [7]	Eating disorder	Helps with diagnosis and disease classification			
Zhang Z. et al., 2017 [8]	Generalized Anxiety Disorder -	1) Helps with diagnosis and disease-related biomarkers 2) Increase the knowledge about disease pathogenesis			
Reid JC. et al., 1994 [9]	Hopelessness		Helps to improve treatment	Helps to better monitor	
Kashani JH. et al., 1996 [10]	Hopelessness	Helps to improve diagnosis	Helps to improve treatment		
Barzman D. et al., 2018	School violence	Helps with disease			

[11]		assessment			
Velupillai S. et al., 2019 [12]	Suicidality	Helps to identify disease			
DiGuiseppi GT. et al., 2020 [13]	Homelessness (following substance use treatment)	Helps with diagnosis			
Fitzgerald A. et al., 2018 [14]	Substance use disorder/behavior	Helps to identify associated risk factors			
Garcia E.G. et al., 2010 [15]	Substance use disorder/behavior	Helps to identify associated risk factors			
Gervilla E. et al., 2011 [16]	Cannabis use disorder	Helps with diagnosis			
Ruan H. et al., 2019 [17]	Alcohol binge drinking	Helps with diagnosis	Helps with targeted interventions		
Squeglia L. et al., 2017 [18]	Alcohol use disorder (underaged drinking)	Helps with diagnosis			
Thakur S. et al., 2016 [19]	Alcohol consumption traits	Helps to identify the alcoholic vs non- alcoholic			
Foland-Ross L. C. et al., 2015 [20]	Depression	Helps understand patterns of brain structure			
Geraci J. et al., 2017 [21]	Depressive/Dysthymi c disorder	Helps with diagnosis			
Downs J. et al., 2019 [22]	Early onset psychosis			Helps in better monitoring antipsychotic-treated adolescents	
Liu, Y. et al., 2018 [23]	Acute onset schizophrenia	Helps with diagnosis			
Lenhard F. et al., 2018 [24]	Obsessive-compulsive disorder			Helps with better monitoring	
Fujisawa T. X. et al., 2018 [25]	Reactive attachment disorder	Helps to improve diagnosis accuracy	Helps to improve treatment accuracy	Helps with better monitoring	
Xue Y. et	Unspecified outcomes		Helps with		

al., 2014 [26]	of other psychological stress/pressure level (mild-moderate level)		treatment		
Jin L. et al., 2016 [27]	Unspecified outcomes of other psychological stress/pressure level	Helps with diagnosis			
Li Y. et al., 2015 [28]	Unspecified outcomes of other psychological stress/pressure level	Helps with diagnosis			
Tyulyupo S. V. et al., 2018 [29]	Psychological well-being estimation			Helps with better monitoring in rural schools	
Gan Y, 2012 [30]	Left behind adolescents' life satisfaction				Helps with better evaluation
Lim J.S. et al., 2022 [31]	Suicide Attempts	Helps in early identification and intervention for suicide risk		Helps in suicide monitoring and prevention	
Lorge I. et al., 2024 [32]	School bullying and eating disorders	Helps to identify individuals who are facing bullying and risk of eating disorders		To help with time efficient monitoring of prevalent mental health factors in adolescents from Reddit posts	
Lv J. et al., 2022 [33]	Depressive symptoms	Helps identify the factors with significant impact on depressive symptoms			
McCoy T.H. et al., 2024 [34]	General Psychopathology	Helps to estimate Research Domain Criteria associated with clinical outcomes			
McIsaac M.A. et al., 2021 [35]	Social determinants of mental health	Helps to identify social locations of adolescents		To determine the differences in mental health experiences between genders	
Mouchabac S. et al., 2021 [36]	Suicidal Relapses	Helps to prevent relapse of suicide attempts within several months		Helps to prevent relapse of suicide attempts	

				within several months	
Nagaoka D. et al., 2024 [37]	Help seeking intention on suicide	Helps to identify clusters impacting adolescents help seeking intention on suicide			
Penfold R.B. et al., 2021 [38]	Suicide attempts and death	Helps for suicide risk prediction		Identify individuals who are at risk and need further evaluation	The prediction target was 90-day risk of suicide attempt following a visit
Qasrawi R. et al., 2022 [39]	Depression and anxiety	Helps prediction of risk factors associated with depression and anxiety			
Qasrawi R. et al., 2023 [40]	Mental health risk factors	Helps to determine the impact of living in politically violent environments on the mental health and cognitive development of children			
Rajapaksha R.M.D.S. et al., 2022 [41]	Cannabic use disorder				Helps to predict personalize d CUD risk in adulthood
Ren Z. et al., 2021[42]	Mood disorders	Helps determine the factors associated with mood disorders			
Rothenberg W.A. et al., 2023 [43]	Mental health	Helps to identify the most important preadolescent risk factors in predicting adolescent mental health			
Santoso	Suicide Ideation	Helps detect			Helps

M.S. et al., 2023 [44]		suicidal behaviour			minimize the number of suicide attempts
Sedgwick R. et al., 2023 [45]	Mental health	Helps understand the online behaviour and its effect on mental health			
Song J.H. et al., 2023 [46]	Stress	Help understand the words on social media that are associated with stress in adolescent			
Stevens H.R. et al., 2021[47]	Mental Health	Helps understand anxiety provoking subjects on reddit for LGBTQ+ adolescents			
Su C. et al., 2020 [48]	Suicide risk prediction			Prediction of suicide risk in adolescents	
van Vuuren C.L. et al., 2021[49]	Suicide behaviour	Helps to better predict suicide behaviour			
Wei Z. et al., 2020 [50]	Suicide Attempts	Helps us predict suicide attempt in adolescent			
Weintraub M.J. et al., 2023 [51]	Depression	Helps us determine depressive symptoms with adolescents with high risk of mood disorders			
Weller O. et al., 2021 [52]	Suicide thoughts and behaviour	Helps us to predict suicide thoughts and behaviours			
Zhang-James Y. et al., 2020 [53]	Substance use disorder in ADHD	Helps predict substance use disorder in ADHD patients		SUD risks can be monitored using longitudinal models over years during child development	
Zhong Y. et	Non-suicidal self	Helps predict non			

al., 2024 [54]	injury	suicidal self-injury			
Zhou S.C. et al., 2024 [55]	Non-suicidal self injury	Helps predict non suicidal self-injury			
Zhou Y. et al., 2024 [56]	Depression			Identifies depression risk in adolescents and provides a methodological reference for large-scale primary screening	
Bajaj S. et al., 2023 [57]	Suicide risk	Identifies structural brain alterations in adolescents that can discriminate individuals with suicide risk from typically developing adolescents using machine learning			
Balano J.B. et al., 2019 [58]	Depression	Uses voice recognition to analyze depression levels in participants based on the BDI-II questionnaire			
Li Q. et al., 2017 [59]	Psychological stress	Identifies stressful periods and stressor events from teens' microblog posts			
Lai T. et al., 2023 [60]	Effects of AI technology on emotional perception	Examines the impact of AI in education on adolescents' emotional perception in China			
Kim D. et al., 2019 [61]	Major Depressive Disorder (MDD)	Used machine learning on MRI data to classify MDD			

Almuqhim F. et al., 2021 [62]	Autism Spectrum Disorder	Develops a deep-learning model to classify ASD from typical control subjects using fMRI data			
Acosta J.R. et al., 2020 [63]	Bipolar disorder	Investigates the association between bullying and the presence of psychotic symptoms in youth with bipolar disorder			
Aziz A. et al., 2021[64]	Online game addiction	Focuses on diagnosing online game addiction among Junior High School students using an expert system			
Beaudequin D. et al., 2021[65]	Psychological distress	Proposes a Bayesian network model to analyze lifestyle and psychosocial variables affecting psychological well-being or distress			
Mullick T. et al., 2022 [66]	Depression	Predicts depression score and change using machine learning models			
Li L. et al., 2021 [67]	Post-traumatic stress disorder (PTSD)	Investigates hippocampal subfields in pediatric PTSD patients to identify structural and functional abnormalities			
Kiss O. et al., 2022 [68]	COVID-19 related mental health issues	Analyzes the psychological impact of the COVID-19 pandemic on young			

		adolescents			
Haag A.C. et al., 2023 [69]	Posttraumatic stress symptoms	Investigates PTSS trajectories in adolescent females following potentially traumatic events			
Kim K.W. et al., 2021 [70]	Various psychiatric conditions	Focuses on classifying high-risk suicide groups among adolescents using machine learning techniques			
Bao J. et al., 2024 [71]	Psychological pain related to suicide	Focuses on classifying SA and NSSI using machine learning			
Chen Q. et al., 2024 [72]	Sensitivity to criticism	Examines neural responses and interactions within the brain when adolescents are exposed to criticism			
Li Q. et al., 2017 [73]	Psychological stress	Focuses on forecasting stress levels in teenagers using co-experiencing stressor events			
Carratalá B.H. et al., 2021 [74]	Stress in academic and sports settings	Analyzes the relationship between stress, coping, resilience, and commitment using AI techniques			
Budler L.C. et al., 2023 [75]	Quality of life and mental well-being	Examines the relationship between HRQoL and mental well-being among Slovenian adolescents			
Arogundade O. et al., 2022 [76]	Depression	Focuses on using video games as a tool to address	Video game therapy		

		depression in teenagers			
Anandkumar K.M. et al., 2023 [77]	Depression	Aims to diagnose depression in teenagers using hybrid model combining Regression and SVM	Self-Care and Guided-Care		
Ali M.R. et al., 2020 [78]	Autism Spectrum Disorder (ASD)	Uses a virtual agent to improve social skills in teenagers with ASD	Social skills training	Monitoring engagement	
Li Q. et al., 2024 [79]	Depression	Predicts depression risk in adolescents using census data and AI			
Ke F. et al., 2020 [80]	Autism Spectrum Disorder (ASD)	Explores representational flexibility development through virtual reality and speech data mining			
Ahuvia I.L. et al., 2023 [81]	Depression	Investigates treatment-matching algorithm for online single-session interventions	Intervention matching	Intervention effectiveness	
Jankowsky K. et al., 2024 [82]	Risk of suicide attempts	Utilizes machine learning to predict suicide attempts among adolescents			
Agarwal P. et al., 2019 [83]	Emotional intelligence assessment	Develops AI-based tool to infer emotional intelligence for counselors			
Bashiri A. et al., 2018 [84]	ADHD	Focuses on recommending computerized cognitive rehabilitation programs	Cognitive rehabilitation		

Bakker T. et al., 2023 [85]	Autism Spectrum Disorder (ASD)	Predicts academic success among autistic students			
Kaiser R.H. et al., 2024 [86]	Mood disorders	Uses neurocognitive variables to predict future mood symptoms		Daily mood monitoring	Predicts mood disorder trajectories
Bae S.M. et al., 2015 [87]	Suicide attempts	Predicts suicide attempts based on sociodemographic and psychological variables			Focus on identifying at-risk adolescents to predict potential suicide attempts
Barrios J. et al., 2023 [88]	Attention-deficit/hyperactivity disorder (ADHD)	Supports ADHD diagnosis using stylometric analysis of autobiographical narratives			

References

1. Bekele, E., et al. *Virtual reality-based facial expressions understanding for teenagers with autism*. in *7th International Conference on Universal Access in Human-Computer Interaction: Design Methods, Tools, and Interaction Techniques for eInclusion, UAHCI 2013, Held as Part of 15th International Conference on Human-Computer Interaction, HCI 2013, July 21, 2013 - July 26, 2013*. 2013. Las Vegas, NV, United states: Springer Verlag.
2. Zhou, Y., Yu, F., & Duong, T. (2014). Multiparametric MRI characterization and prediction in autism spectrum disorder using graph theory and machine learning. *PLOS ONE, 9*(6), e90405. https://doi.org/10.1371/journal.pone.0090405
3. Chen H, Duan X, Liu F, Lu F, Ma X, Zhang Y, Uddin LQ, Chen H. Multivariate classification of autism spectrum disorder using frequency-specific resting-state functional connectivity--A multi-center study. Prog Neuropsychopharmacol Biol Psychiatry. 2016 Jan 4;64:1-9. doi: 10.1016/j.pnpbp.2015.06.014. Epub 2015 Jul 4. PMID: 26148789.
4. Hart H, Chantiluke K, Cubillo AI, Smith AB, Simmons A, Brammer MJ, Marquand AF, Rubia K. Pattern classification of response inhibition in ADHD: toward the development of neurobiological markers for ADHD. Hum Brain Mapp. 2014 Jul;35(7):3083-94. doi: 10.1002/hbm.22386. Epub 2013 Oct 11. PMID: 24123508; PMCID: PMC4190683.
5. Khaleghi A, Sheikhani A, Mohammadi MR, Nasrabadi AM, Vand SR, Zarafshan H, Moeini M. EEG classification of adolescents with type I and type II of bipolar disorder.

Australas Phys Eng Sci Med. 2015 Dec;38(4):551-9. doi: 10.1007/s13246-015-0375-0. PMID: 26472650.

6. Ang RP, Goh DH. Predicting juvenile offending: a comparison of data mining methods. Int J Offender Ther Comp Criminol. 2013 Feb;57(2):191-207. doi: 10.1177/0306624X11431132. Epub 2011 Dec 12. PMID: 22158911.
7. Strigo IA, Murray SB, Simmons AN, Bernard RS, Huang JS, Kaye WH. The clinical application of fMRI data in a single-patient diagnostic conundrum: Classifying brain response to experimental pain to distinguish between gastrointestinal, depressive and eating disorder symptoms. J Clin Neurosci. 2017 Nov;45:149-153. doi: 10.1016/j.jocn.2017.07.023. Epub 2017 Aug 16. PMID: 28823587.
8. Zhang Z, Liao M, Yao Z, Hu B, Xie Y, Zheng W, Hu T, Zhao Y, Yang F, Zhang Y, Su L, Li L, Gutknecht J, Majoe D. Frequency-Specific Functional Connectivity Density as an Effective Biomarker for Adolescent Generalized Anxiety Disorder. Front Hum Neurosci. 2017 Dec 5;11:549. doi: 10.3389/fnhum.2017.00549. PMID: 29259549; PMCID: PMC5723402.
9. Reid JC, Nair SS, Kashani JH, Rao VG. Detecting dysfunctional behavior in adolescents: the examination of relationships using neural networks. Proc Annu Symp Comput Appl Med Care. 1994:743-6. PMID: 7950023; PMCID: PMC2247956.
10. Kashani JH, Nair SS, Rao VG, Nair J, Reid JC. Relationship of personality, environmental, and DICA variables to adolescent hopelessness: a neural network sensitivity approach. J Am Acad Child Adolesc Psychiatry. 1996 May;35(5):640-5. doi: 10.1097/00004583-199605000-00019. PMID: 8935211.
11. Barzman D, Ni Y, Griffey M, Bachtel A, Lin K, Jackson H, Sorter M, DelBello M. Automated Risk Assessment for School Violence: a Pilot Study. Psychiatr Q. 2018 Dec;89(4):817-828. doi: 10.1007/s11126-018-9581-8. PMID: 29713946.
12. Velupillai S, Epstein S, Bittar A, Stephenson T, Dutta R, Downs J. Identifying Suicidal Adolescents from Mental Health Records Using Natural Language Processing. Stud Health Technol Inform. 2019 Aug 21;264:413-417. doi: 10.3233/SHTI190254. PMID: 31437956.
13. DiGuiseppi GT, Davis JP, Leightley D, Rice E. Predictors of Adolescents' First Episode of Homelessness Following Substance Use Treatment. J Adolesc Health. 2020 Apr;66(4):408-415. doi: 10.1016/j.jadohealth.2019.11.312. Epub 2020 Feb 10. PMID: 32057607.
14. Fitzgerald, A., Mac Giollabhui, N., Dolphin, L., Whelan, R., & Dooley, B. (2018). Dissociable psychosocial profiles of adolescent substance users. PLOS ONE, 13(8), e0202498. https://doi.org/10.1371/journal.pone.0202498
15. García EG, Blasco BC, López RJ, Pol AP. Study of the factors associated with substance use in adolescence using Association Rules. Adicciones. 2010;22(4):293-9. PMID: 21152847.
16. Gervilla E, Cajal B, Palmer A. Quantification of the influence of friends and antisocial behaviour in adolescent consumption of cannabis using the ZINB model and data mining. Addict Behav. 2011 Apr;36(4):368-74. doi: 10.1016/j.addbeh.2010.12.007. Epub 2010 Dec 10. PMID: 21190799.
17. Ruan H, Zhou Y, Luo Q, Robert GH, Desrivières S, Quinlan EB, Liu Z, Banaschewski T, Bokde ALW, Bromberg U, Büchel C, Flor H, Frouin V, Garavan H, Gowland P, Heinz A, Ittermann B, Martinot JL, Martinot MP, Nees F, Orfanos DP, Poustka L, Hohmann S,

Fröhner JH, Smolka MN, Walter H, Whelan R, Li F, Schumann G, Feng J; IMAGEN Consortium. Adolescent binge drinking disrupts normal trajectories of brain functional organization and personality maturation. Neuroimage Clin. 2019;22:101804. doi: 10.1016/j.nicl.2019.101804. Epub 2019 Mar 31. PMID: 30991616; PMCID: PMC6451196.

18. Squeglia LM, Ball TM, Jacobus J, Brumback T, McKenna BS, Nguyen-Louie TT, Sorg SF, Paulus MP, Tapert SF. Neural Predictors of Initiating Alcohol Use During Adolescence. Am J Psychiatry. 2017 Feb 1;174(2):172-185. doi: 10.1176/appi.ajp.2016.15121587. Epub 2016 Aug 19. Erratum in: Am J Psychiatry. 2017 Jan 1;174(1):80. doi: 10.1176/appi.ajp.2016.1741correction. PMID: 27539487; PMCID: PMC5288131.
19. Thakur, S. (2016). Identification of Chief Characteristics of Alcohol Consumption Traits in Schools Using Rough Set and Formal Concept Analysis.
20. Foland-Ross LC, Sacchet MD, Prasad G, Gilbert B, Thompson PM, Gotlib IH. Cortical thickness predicts the first onset of major depression in adolescence. Int J Dev Neurosci. 2015 Nov;46:125-31. doi: 10.1016/j.ijdevneu.2015.07.007. Epub 2015 Aug 24. PMID: 26315399; PMCID: PMC4604750.
21. Geraci J, Wilansky P, de Luca V, Roy A, Kennedy JL, Strauss J. Applying deep neural networks to unstructured text notes in electronic medical records for phenotyping youth depression. Evid Based Ment Health. 2017 Aug;20(3):83-87. doi: 10.1136/eb-2017-102688. Epub 2017 Jul 24. PMID: 28739578; PMCID: PMC5566092.
22. Downs J, Dean H, Lechler S, Sears N, Patel R, Shetty H, Hotopf M, Ford T, Kyriakopoulos M, Diaz-Caneja CM, Arango C, MacCabe JH, Hayes RD, Pina-Camacho L. Negative Symptoms in Early-Onset Psychosis and Their Association With Antipsychotic Treatment Failure. Schizophr Bull. 2019 Jan 1;45(1):69-79. doi: 10.1093/schbul/sbx197. PMID: 29370404; PMCID: PMC6293208.
23. Liu Y, Guo W, Zhang Y, Lv L, Hu F, Wu R, Zhao J. Decreased Resting-State Interhemispheric Functional Connectivity Correlated with Neurocognitive Deficits in Drug-Naive First-Episode Adolescent-Onset Schizophrenia. Int J Neuropsychopharmacol. 2018 Jan 1;21(1):33-41. doi: 10.1093/ijnp/pyx095. PMID: 29228204; PMCID: PMC5795351.
24. Lenhard F, Sauer S, Andersson E, Månsson KN, Mataix-Cols D, Rück C, Serlachius E. Prediction of outcome in internet-delivered cognitive behaviour therapy for paediatric obsessive-compulsive disorder: A machine learning approach. Int J Methods Psychiatr Res. 2018 Mar;27(1):e1576. doi: 10.1002/mpr.1576. Epub 2017 Jul 28. PMID: 28752937; PMCID: PMC6877165.
25. Fujisawa TX, Shimada K, Takiguchi S, Mizushima S, Kosaka H, Teicher MH, Tomoda A. Type and timing of childhood maltreatment and reduced visual cortex volume in children and adolescents with reactive attachment disorder. Neuroimage Clin. 2018 Jul 23;20:216-221. doi: 10.1016/j.nicl.2018.07.018. PMID: 30094171; PMCID: PMC6080635.
26. Xue, Y., Li, Q., Jin, L., Feng, L., Clifton, D.A., Clifford, G.D. (2014). Detecting Adolescent Psychological Pressures from Micro-Blog. In: Zhang, Y., Yao, G., He, J., Wang, L., Smalheiser, N.R., Yin, X. (eds) Health Information Science. HIS 2014. Lecture Notes in Computer Science, vol 8423. Springer, Cham. https://doi.org/10.1007/978-3-319-06269-3_10

27. Jin, L., Xue, Y., Li, Q., Feng, L. (2016). Integrating Human Mobility and Social Media for Adolescent Psychological Stress Detection. In: Navathe, S., Wu, W., Shekhar, S., Du, X., Wang, S., Xiong, H. (eds) Database Systems for Advanced Applications. DASFAA 2016. Lecture Notes in Computer Science(), vol 9643. Springer, Cham. https://doi.org/10.1007/978-3-319-32049-6_23
28. Y. Li, J. Huang, H. Wang and L. Feng, "Predicting Teenager's Future Stress Level from Micro-Blog," 2015 IEEE 28th International Symposium on Computer-Based Medical Systems, Sao Carlos, Brazil, 2015, pp. 208-213, doi: 10.1109/CBMS.2015.25.
29. S. V. Tyulyupo, A. A. Andrakhanov, B. A. Dashieva and A. V. Tyryshkin, "Adolescents Psychological Well-Being Estimation Based on a Data Mining Algorithm," 2018 IEEE 13th International Scientific and Technical Conference on Computer Sciences and Information Technologies (CSIT), Lviv, Ukraine, 2018, pp. 475-478, doi: 10.1109/STC-CSIT.2018.8526628.
30. Y. -y. Gan.(2012)) Evaluation on life satisfaction of left-behind junior high school children based on LVQ network, 8th International Conference on Natural Computation, Chongqing, China, 2012, pp. 405-408, doi: 10.1109/ICNC.2012.6234755.
31. Lim JS, Yang CM, Baek JW, Lee SY, Kim BN. Prediction Models for Suicide Attempts among Adolescents Using Machine Learning Techniques. Clin Psychopharmacol Neurosci. 2022 Nov 30;20(4):609-620. doi: 10.9758/cpn.2022.20.4.609. PMID: 36263637; PMCID: PMC9606439.
32. Lorge, I., D.W. Joyce, and A. Kormilitzin, *Large Language Models Perform on Par with Experts Identifying Mental Health Factors in Adolescent Online Forums*. 2024: USA.
33. Lv, J., et al., *The cross-sectional study of depressive symptoms and associated factors among adolescents by backpropagation neural network.* Public Health, 2022. **208**: p. 52-58.
34. McCoy, T.H. and R.H. Perlis, *Dimensional measures of psychopathology in children and adolescents using large language models.* Biological psychiatry, 2024.
35. McIsaac, M.A., et al., *A novel application of a data mining technique to study intersections in the social determinants of mental health among young Canadians.* SSM-POPULATION HEALTH, 2021. **16**.
36. Mouchabac, S., et al., *Prevention of Suicidal Relapses in Adolescents With a Smartphone Application: Bayesian Network Analysis of a Preclinical Trial Using In Silico Patient Simulations.* JOURNAL OF MEDICAL INTERNET RESEARCH, 2021. **23**(9).
37. Nagaoka, D., et al., *Identify adolescents' help-seeking intention on suicide through self- and caregiver's assessments of psychobehavioral problems: deep clustering of the Tokyo TEEN Cohort study.* LANCET REGIONAL HEALTH-WESTERN PACIFIC, 2024. **43**.
38. Penfold, R.B., et al., *Predicting suicide attempts and suicide deaths among adolescents following outpatient visits.* JOURNAL OF AFFECTIVE DISORDERS, 2021. **294**: p. 39-47.
39. Qasrawi, R., et al., *Assessment and Prediction of Depression and Anxiety Risk Factors in Schoolchildren: Machine Learning Techniques Performance Analysis.* JMIR formative research, 2022. **6**(8): p. e32736.
40. Qasrawi, R., et al., *Machine learning techniques for identifying mental health risk factor associated with schoolchildren cognitive ability living in politically violent environments.* FRONTIERS IN PSYCHIATRY, 2023. **14**.

41. Rajapaksha, R.M.D.S., et al., *A Bayesian learning model to predict the risk for cannabis use disorder.* Drug and alcohol dependence, 2022. **236**: p. 109476.
42. Ren, Z., et al., *What Factors Are Most Closely Associated With Mood Disorders in Adolescents During the COVID-19 Pandemic? A Cross-Sectional Study Based on 1,771 Adolescents in Shandong Province, China.* Frontiers in Psychiatry, 2021. **12**: p. 728278.
43. Rothenberg, W.A., et al., *Predicting Adolescent Mental Health Outcomes Across Cultures: A Machine Learning Approach.* Journal of youth and adolescence, 2023. **52**(8): p. 1595-1619.
44. Santoso, M.S., et al., *A Comparative Analysis of Decision Tree and Support Vector Machine on Suicide Ideation Detection.* Procedia Computer Science, 2023: p. 518-23.
45. Sedgwick, R., et al., *Investigating online activity in UK adolescent mental health patients: A feasibility study using a natural language processing approach for electronic health records.* BMJ Open, 2023. **13**(5): p. e061640.
46. Song, J.H., et al., *Exploring Korean adolescent stress on social media: a semantic network analysis.* PeerJ, 2023. **11**: p. e15076.
47. Stevens, H.R., I. Acic, and S. Rhea, *Natural Language Processing Insight into LGBTQ plus Youth Mental Health During the COVID-19 Pandemic: Longitudinal Content Analysis of Anxiety-Provoking Topics and Trends in Emotion in LGBTeens Microcommunity Subreddit.* JMIR PUBLIC HEALTH AND SURVEILLANCE, 2021. **7**(8).
48. Su, C., et al., *Machine learning for suicide risk prediction in children and adolescents with electronic health records.* Translational Psychiatry, 2020. **10**(1): p. 413.
49. van Vuuren, C.L., et al., *Comparing machine learning to a rule-based approach for predicting suicidal behavior among adolescents: Results from a longitudinal population-based survey.* Journal of Affective Disorders, 2021. **295**: p. 1415-1420.
50. Wei, Z. and S. Mukherjee, *Health-Behaviors Associated With the Growing Risk of Adolescent Suicide Attempts: A Data-Driven Cross-Sectional Study.* American journal of health promotion : AJHP, 2021. **35**(5): p. 688-693.
51. Weintraub, M.J., et al., *Word usage in spontaneous speech as a predictor of depressive symptoms among youth at high risk for mood disorders.* Journal of Affective Disorders, 2023. **323**: p. 675-678.
52. Weller, O., et al., *Predicting suicidal thoughts and behavior among adolescents using the risk and protective factor framework: A large-scale machine learning approach.* PLoS ONE, 2021. **16**(November): p. e0258535.
53. Zhang-James, Y., et al., *Machine-Learning prediction of comorbid substance use disorders in ADHD youth using Swedish registry data.* JOURNAL OF CHILD PSYCHOLOGY AND PSYCHIATRY, 2020. **61**(12): p. 1370-1379.
54. Zhong, Y., et al., *A machine learning algorithm-based model for predicting the risk of non-suicidal self-injury among adolescents in western China: A multicentre cross-sectional study.* Journal of affective disorders, 2024. **345**: p. 369-377.
55. Zhou, S.C., et al., *Prediction of non-suicidal self-injury in adolescents at the family level using regression methods and machine learning.* Journal of Affective Disorders, 2024. **352**: p. 67-75.
56. Zhou, Y., et al., *Identifying the risk of depression in a large sample of adolescents: An artificial neural network based on random forest.* Journal of Adolescence, 2024.

57. Bajaj, S., et al., *Machine learning based identification of structural brain alterations underlying suicide risk in adolescents.* Discover Mental Health, 2023. **3**(1): p. 6.
58. Balano, J.B., et al., *Determining the Level of Depression using BDI-II through Voice Recognition*, in *2019 IEEE 6TH INTERNATIONAL CONFERENCE ON INDUSTRIAL ENGINEERING AND APPLICATIONS (ICIEA)*. 2019. p. 387-391.
59. Li, Q., et al., *Analyzing and Identifying Teens' Stressful Periods and Stressor Events From a Microblog.* IEEE journal of biomedical and health informatics, 2017. **21**(5): p. 1434-1448.
60. Lai, T., et al., *The application of artificial intelligence technology in education influences Chinese adolescent's emotional perception.* Current psychology (New Brunswick, N.J.), 2023: p. 1-9.
61. D. Kim et al., "Machine Learning Classification of First-Onset Drug-Naive MDD Using Structural MRI," in IEEE Access, vol. 7, pp. 153977-153985, 2019, doi: 10.1109/ACCESS.2019.2949128.
62. Almuqhim, F. and F. Saeed, *ASD-SAENet: A Sparse Autoencoder, and Deep-Neural Network Model for Detecting Autism Spectrum Disorder (ASD) Using fMRI Data.* Frontiers in Computational Neuroscience, 2021. **15**: p. 654315.
63. Acosta, J.R., et al., *Bullying and psychotic symptoms in youth with bipolar disorder.* JOURNAL OF AFFECTIVE DISORDERS, 2020. **265**: p. 603-610.
64. Aziz, A., B.W. Setyawan, and K. Saddhono, *Using expert system application to diagnose online game addiction in junior high school students: case study in five big city in Indonesia.* Ingenierie des systemes d'information, 2021. **26**(5): p. 445-52.
65. Beaudequin, D., et al., *A novel, complex systems approach to modelling risk of psychological distress in young adolescents.* SCIENTIFIC REPORTS, 2021. **11**(1).
66. Mullick, T., Radovic, A., Shaaban, S., & Doryab, A. (2022). Predicting depression in adolescents using mobile and wearable sensors: Multimodal machine learning–based exploratory study. JMIR Formative Research, 6(6), e35807. https://doi.org/10.2196/35807
67. Li, L., et al., *Hippocampal subfield alterations in pediatric patients with post-traumatic stress disorder.* Social cognitive and affective neuroscience, 2021. **16**(3): p. 334-344.
68. Kiss, O., et al., *The Pandemic's Toll on Young Adolescents: Prevention and Intervention Targets to Preserve Their Mental Health.* JOURNAL OF ADOLESCENT HEALTH, 2022. **70**(3): p. 387-395.
69. Haag, A.-C., et al., *Understanding posttraumatic stress trajectories in adolescent females: A strength-based machine learning approach examining risk and protective factors including online behaviors.* Development and psychopathology, 2023. **35**(4): p. 1794-1807.
70. Kim, K.-W., et al., *Classification of Adolescent Psychiatric Patients at High Risk of Suicide Using the Personality Assessment Inventory by Machine Learning.* Psychiatry investigation, 2021. **18**(11): p. 1137-1143.
71. Bao, J., et al., *Psychological pain and sociodemographic factors classified suicide attempt and non-suicidal self-injury in adolescents.* Acta psychologica, 2024. **246**: p. 104271.
72. Chen, Q., et al., *Unraveling how the adolescent brain deals with criticism using dynamic causal modeling.* NeuroImage, 2024. **286**: p. 120510.

73. Li, Q., Zhao, L., Xue, Y., Jin, L., & Feng, L. (2017). Exploring the impact of co-experiencing stressor events for teens stress forecasting. In W. Wang, Y. Huang, & Y. Xie (Eds.), Web Information Systems Engineering – WISE 2017: 18th International Conference, Puschino, Russia, October 7–11, 2017, Proceedings, Part II (pp. 313–328). Springer. https://doi.org/10.1007/978-3-319-68786-5_25
74. Bellod, H.C., et al., *Analysis of stress and academic-sports commitment through Self-organizing Artificial Neural Networks.* RETOS-NUEVAS TENDENCIAS EN EDUCACION FISICA DEPORTE Y RECREACION, 2021(42): p. 136-144.
75. Cilar Budler, L. and G. Stiglic, *Age, quality of life and mental well-being in adolescent population: a network model tree analysis.* Scientific reports, 2023. **13**(1): p. 17667.
76. Arogundade, O., et al. *Psychotherapeutic Tool for Addressing Depression in Teenagers Through Video Games*. in *International Conference on Hybrid Intelligent Systems, 14-16 Dec. 2021*. 2022. Berlin, Germany: Springer.
77. Anandkumar, K.M., et al. *SOLWOE—A Novel Way to Diagnose Depression Among Teenagers*. in *6th International Conference on Innovative Computing and Communication, ICICC 2023, February 17, 2023 - February 18, 2023*. 2023. Delhi, India: Springer Science and Business Media Deutschland GmbH.
78. Ali, M.R., et al., *A Virtual Conversational Agent for Teens with Autism Spectrum Disorder: Experimental Results and Design Lessons*, in *PROCEEDINGS OF THE 20TH ACM INTERNATIONAL CONFERENCE ON INTELLIGENT VIRTUAL AGENTS (ACM IVA 2020)*. 2020.
79. Li, Q., et al., *Exploration of Adolescent Depression Risk Prediction Based on Census Surveys and General Life Issues*. 2024, arXiv.
80. Ke, F., J. Moon, and Z. Sokolikj. *Tracking Representational Flexibility Development through Speech Data Mining*. in *2020 IEEE Frontiers in Education Conference, FIE 2020, October 21, 2020 - October 24, 2020*. 2020. Uppsala, Sweden: Institute of Electrical and Electronics Engineers Inc.
81. Ahuvia, I.L., et al., *Evaluating a treatment selection approach for online single-session interventions for adolescent depression.* JOURNAL OF CHILD PSYCHOLOGY AND PSYCHIATRY, 2023. **64**(12).
82. Jankowsky, K., D. Steger, and U. Schroeders, *Predicting Lifetime Suicide Attempts in a Community Sample of Adolescents Using Machine Learning Algorithms.* Assessment, 2024. **31**(3): p. 557-573.
83. Agarwal, P., et al. *Multimodal Web Application to Infer Emotional Intelligence of Adolescent Counsellor*. in *2019 Grace Hopper Celebration India, GHCI 2019, November 6, 2019 - November 8, 2019*. 2019. Bangalore, India: Institute of Electrical and Electronics Engineers Inc.
84. Bashiri, A., et al. *Designing a Clinical Decision Support System for Recommending Computerized Cognitive Rehabilitation Programs: The Experience of Attention Deficit Hyperactivity Disorder*. in *2nd National and 1st International Digital Games Research Conference: Trends, Technologies, and Applications, DGRC 2018, November 29, 2018 - November 30, 2018*. 2018. Tehran, Iran: Institute of Electrical and Electronics Engineers Inc.
85. Bakker, T., et al., *Predicting academic success of autistic students in higher education.* Autism, 2023. **27**(6): p. 1803-1816.

86. Kaiser, R.H., et al., *Neurocognitive Risk Phenotyping to Predict Mood Symptoms in Adolescence.* Journal of Psychopathology and Clinical Science, 2024. **133**(1): p. 90-102.
87. Bae SM, Lee SA, Lee SH. Prediction by data mining, of suicide attempts in Korean adolescents: a national study. Neuropsychiatr Dis Treat. 2015 Sep 16;11:2367-75. doi: 10.2147/NDT.S91111. PMID: 26396521; PMCID: PMC4577255.
88. Barrios, J., Gabay, S., Cafiero, F., & Debbané, M. (2023, October 17). Detecting Psychological Disorders with Stylometry: the Case of ADHD in Adolescent Autobiographical Narratives. https://doi.org/10.31234/osf.io/s5cm3
