# Supplementary material for: Use of Artificial Intelligence in Adolescents’ Mental Health Care: Systematic Scoping Review of Current Applications and Future Directions
Source: JMIR Ment Health. 2025 Jun 6;12:e70438. doi: 10.2196/70438 (PMC12165596; doi:10.2196/70438)
Supplement: Multimedia Appendix 4 [file mental-v12-e70438-s004.pdf]

Multimedia Appendix 4: Risk of Bias (ROB) table: based on authors' judgements about each risk of bias item. (+ Low ROB , - High ROB, ? Unclear ROB)

Author	Participants		Predictors			Outcome						Analysis								
	Were appropriate data sources used, e.g: cohort, RCT, or nested case-control study data?	Were all inclusions and exclusions of participants appropriate?	Were predictors defined and assessed in a similar way for all participants ?	Were predictor assessments made without knowledge of outcome data?	Are all predictors available at the time the model is intended to be used?	Was the outcome determined appropriately?	Was a prespecified or standard outcome definition used?	Were predictors excluded from the outcome definition?	Was the outcome defined and determined in a similar way for all participants?	Was the outcome determined without knowledge of predictor information?	Was the time interval between predictor assessment and outcome determination appropriate?	Were there a reasonable number of participants with the outcome?	Were continuous and categorical predictors handled appropriately?	Were all enrolled participants included in the analysis?	Were participants with missing data handled appropriately?	Was selection of predictors based on univariable analysis avoided? (For developmental studies only)	Were complexities in the data (e.g., censoring, competing risks, sampling of control participants) accounted for appropriately?	Were relevant model performance measures evaluated appropriately?	Were model overfitting, underfitting, and optimism in model performance accounted for? (For developmental studies only)	Do predictors and their assigned weights in the final model correspond to the results from the reported multivariable analysis? (For developmental studies only)
Khaleghi, Ali,Sheikhani, Ali,Mohammadi, Mohammad Reza,Nasrabadi, Ali Moti,Vand, Safa Rafiei,Zarafshan, Hadi,Moeini, Mahdi [1]	+	+	+	+	+	+	-	-	+	-	?	-	+	+	?	+	+	-	-	+

Velupillai, Sumithra,Epstein, Sophie,Bittar, Andre,Stephenson, Thomas,Dutta, Rina,Downs, Johnny [2]	+	+	+	+	+	+	+	+	+	+	+	+	+	+	?	?	?	+	-	?
Jin, Li,Xue, Yuanyuan,Li, Qi,Feng, Ling [3]	+	+	-	-	+	-	+	+	+	+	-	-	?	+	-	?	?	+	?	?
Gan, Yuan-Yuan [4]	+	+	+	-	-	-	-	+	+	+	?	-	?	+	?	-	-	+	-	+
Tyulyupo, S. V.,Andrakhanov, A. A.,Dashieva, B. A.,Tyryshkin, A. V. [5]	+	+	+	-	+	+	-	-	+	+	?	-	?	+	?	?	+	+	-	+
Xue, Yuanyuan,Li, Qi,Jin, Li,Feng, Ling,Clifton, David A.,Clifford, Gari D. [6]	-	+	+	+	+	+	+	+	+	+	+	-	-	+	-	-	-	+	-	+
Strigo, I. A.,Murray, S. B.,Simmons, A. N.,Bernard, R. S.,Huang, J. S.,Kaye, W. H. [7]	-	+	+	+	+	+	-	-	+	-	+	-	+	+	+	NA	?	-	NA	NA

Zhang, Z.,Liao, M.,Yao, Z.,Hu, B.,Xie, Y.,Zheng, W.,Hu, T.,Zhao, Y.,Yang, F.,Zhang, Y.,Su, L.,Li, L.,Gutknecht, J.,Majoe, D. [8]	+	+	+	+	+	+	+	+	+	+	+	-	-	+	?	?	-	+	-	+
Duan, X.,Liu, F.,Lu, F.,Ma, X.,Zhang, Y.,Uddin, L. Q.,Chen, H. [9]	+	+	+	+	+	+	+	+	+	+	+	-	+	+	-	-	+	+	-	+
Thakur, S.,Chowdhury, R. [10]	+	-	+	-	?	-	+	+	+	-	?	+	-	-	?	-	-	-	-	+
Foland-Ross, L. C.,Sacchet, M. D.,Prasad, G.,Gilbert, B.,Thompson, P. M.,Gotlib, I. H. [11]	+	+	+	+	+	+	+	+	+	+	+	-	+	+	?	+	+	+	+	+
Hart, H.,Chantiluke, K.,Cubillo, A. I.,Smith, A. B.,Simmons, A.,Brammer, M. J.,Marquand, A. F.,Rubia, K. [12]	+	-	+	-	+	-	+	-	+	-	+	-	-	+	?	-	-	+	-	+
Zhou, Y.,Yu,	+	+	+	+	+	+	+	-	+	+	+	+	-	+	-	?	+	+	-	+

F.,Duong, T. [13]																				
Ang, R. P.,Goh, D. H. [14]	+	+	+	-	+	+	+	+	+	+	+	+	+	+	-	+	+	+	+	+
Gervilla, E.,Cajal, B.,Palmer, A. [15]	+	+	+	+	+	+	+	+	+	+	?	+	-	+	?	-	+	+	+	+
Kashani, J. H.,Nair, S. S.,Rao, V. G.,Nair, J.,Reid, J. C. [16]	+	+	+	+	+	+	+	+	+	+	-	-	-	+	-	NA	-	+	NA	NA
Reid, J. C.,Nair, S. S.,Kashani, J. H.,Rao, V. G. [17]	+	+	+	-	+	+	-	-	+	+	+	+	+	+	-	NA	-	+	NA	NA
Yiping, Li,Jing, Huang,Hao, Wang,Ling, Feng [18]	?	+	+	+	+	+	+	+	+	+	+	?	+	?	?	+	-	+	+	+
DiGuiseppi, G. T.,Davis, J. P.,Leightley, D.,Rice, E. [19]	+	+	+	+	+	+	+	+	+	+	+	-	+	+	?	?	+	+	+	+

Ruan, H.,Zhou, Y.,Luo, Q.,Robert, G. H.,Desrivieres, S.,Quinlan, E. B.,Liu, Z.,Banaschewski, T.,Bokde, A. L. W.,Bromberg, U.,Buchel, C.,Flor, H.,Frouin, V.,Garavan, H.,Gowland, P.,Heinz, A.,Ittermann, B.,Martinot, J. L.,Martinot, M. P.,Nees, F.,Orfanos, D. P.,Poustka, L.,Hohmann, S.,Frohner, J. H.,Smolka, M. N.,Walter, H.,Whelan, R.,Li, F.,Schumann, G.,Feng, J.,Imagen Consortium [20]	+	+	+	+	+	+	+	+	+	+	+	-	+	+	+	+	-	+	+	+

Downs, J.,Dean, H.,Lechler, S.,Sears, N.,Patel, R.,Shetty, H.,Hotopf, M.,Ford, T.,Kyriakopoulos, M.,Diaz-Caneja, C. M.,Arango, C.,MacCabe, J. H.,Hayes, R. D.,Pina-Camacho, L. [21]	+	?	+	+	+	+	+	-	+	+	+	+	-	+	?	+	-	+	+	+
Fitzgerald, A.,Mac Giollabhui, N.,Dolphin, L.,Whelan, R.,Dooley, B. [22]	+	+	+	+	+	+	+	+	+	+	?	+	+	+	?	+	?	+	+	+
Fujisawa, T. X.,Shimada, K.,Takiguchi, S.,Mizushima, S.,Kosaka, H.,Teicher, M. H.,Tomoda, A. [23]	+	-	-	+	+	+	+	+	+	+	+	-	-	+	?	+	?	+	-	+
Barzman, D.,Ni, Y.,Griffey, M.,Bachtel, A.,Lin, K.,Jackson, H.,Sorter,	+	+	+	+	+	+	+	+	+	+	-	+	-	+	-	NA	-	+	NA	NA

M.,DelBello, M. [24]																				
Lenhard, F.,Sauer, S.,Andersson, E.,Mansson, K. N.,Mataix-Cols, D.,Ruck, C.,Serlachius, E. [25]	+	**?**	+	+	+	+	+	+	+	+	+	**-**	+	+	+	+	**-**	+	+	+
Geraci, J.,Wilansky, P.,de Luca, V.,Roy, A.,Kennedy, J. L.,Strauss, J. [26]	+	+	+	+	+	+	+	+	+	+	+	+	+	+	+	+	**-**	+	+	+
Squeglia, L. M.,Ball, T. M.,Jacobus, J.,Brumback, T.,McKenna, B. S.,Nguyen-Louie, T. T.,Sorg, S. F.,Paulus, M. P.,Tapert, S. F. [27]	+	+	+	+	+	+	+	+	+	**-**	**?**	**-**	**-**	+	**-**	+	**-**	+	+	+
Fahad Almuqhim and Fahad Saeed [28]	+	+	+	+	+	+	+	+	+	+	+	+	+	+	+	+	+	+	+	+

Helio Carratalá Bellod, Vicente Buendía Ramón, Eduardo Carballeira Fernández, José Francisco Guzmán Luján [29]	+	+	+	?	?	+	+	+	+	?	+	+	+	+	?	+	?	+	?	+
Lei Li, Nanfang Pan, Lianqing Zhang, Su Lui, Xiaoqi Huang, Xin Xu, Song Wang, Du Lei, Lingjiang Li, Graham J. Kemp, and Qiyong Gong [30]	+	+	+	+	+	+	+	+	+	+	+	+	+	+	?	+	+	+	+	+
Donghwa Kim, Pilsung Kang, Junhong Kim, Czang Yeob Kim, Jong-Ha Lee, Sangil Suh, and Moon-Soo Lee [31]	+	+	+	+	+	+	+	+	+	+	+	+	+	+	+	+	+	+	+	+
Kyung-Won Kim, Jae Seok Lim, Chan-Mo Yang, Seung-Ho Jang, Sang-Yeol Lee [32]	+	+	+	+	+	+	+	+	+	+	+	+	+	+	+	+	+	+	+	+

Orsolya Kiss, Elisabet Alzueta, Dilara Yuksel, Kilian M. Pohl, Massimiliano de Zambotti, Eva M. M}uller-Oehring, Devin Prouty, Ingrid Durley, William E. Pelham III, Connor J. McCabe, Marybel R. Gonzalez, Sandra A. Brown, Natasha E. Wade, Andrew T. Marshall, Elizabeth R. Sowell, Florence J. Breslin, Krista M. Lisdahl, Anthony S. Dick, Chandni S. Sheth, Bruce D. McCandliss, Mathieu Guillaume, Amandine M. Van Rinsveld, Gayathri J. Dowling, Susan F. Tapert, Fiona C.	+	+	+	+	+	+	+	+	+	+	+	+	+	+	+	+	+	+	+	+

Baker [33]																				

Denise Beaudequin, Paul Schwenn, Larisa T. McLoughlin, Marcella Parker, Amanda Boyes, Gabrielle Simcock, Jim Lagopoulos, Daniel F. Hermens [34]	+	+	+	?	+	+	+	+	+	?	+	+	+	+	?	+	+	+	+	+
Jiamin Bao, Jiachen Wan, Huanhuan Li, Fang Sun [35]	+	+	+	+	?	+	+	+	+	+	+	+	+	+	+	-	?	+	+	+
Ann-Christin Haag, George A. Bonanno, Shuquan Chen, Toria Herd, Sienna Strong-Jones, Sunshine S., Jennie G. Noll [36]	+	+	+	+	+	+	+	+	+	+	+	+	+	+	+	+	+	+	+	+
Sung M Bae, Seung A Lee, Seung-Hwan Lee [37]	+	+	+	?	?	+	+	+	+	?	+	+	+	+	+	+	?	+	+	+

Roselinde H. Kaiser, Amelia D. Moser, Chiara Neilson, Jenna Jones, Elena C. Peterson, Luke Ruzic, Benjamin M. Rosenberg, Christina M. Hough, Christina Sandman, Christopher D. Schneck, David J. Miklowitz. [38]	+	+	+	?	?	+	+	+	+	?	+	+	+	+	+	+	?	+	+	+
Leona Cilar Budler, Gregor Stiglic [39]	+	+	+	+	+	+	+	+	+	+	+	+	+	+	+	+	+	+	+	+
Sahil Bajaj, Karina S. Blair, Matthew Dobbertin, Kaustubh R. Patil, Patrick M. Tyler, Jay L. Ringle, Johannah Bashford-Largo, Avantika Mathur, Jaimie Elowsky, Ahria Dominguez, Lianne Schmaal, R. James R. Blair [40]	+	+	+	+	+	+	+	+	+	+	+	+	+	+	+	+	+	+	+	+

Justin Brian Balano, Vanessa Ley Huerto, Sigfried Sanchez, Aresh Saharkhiz, Joel De Goma [41]	+	+	+	?	?	+	+	+	+	?	+	+	+	+	?	+	?	+	+	+
Juan Barrios, Simon Gabay, Florian Cafiero, Martin Debbané [42]	+	+	+	?	?	+	+	+	+	?	+	+	+	+	?	+	?	+	+	+
J. S. Lim, C. M. Yang, J. W. Baek, S. Y. Lee and B. N. Kim [43]	+	+	+	-	+	+	?	?	+	-	+	+	+	+	?	+	-	+	+	+
R. B. Penfold, E. Johnson, S. M. Shortreed, R. A. Ziebell, F. L. Lynch, G. N. Clarke, et al. [44]	+	+	+	-	+	+	+	+	+	-	+	+	+	+	?	+	?	+	?	+
R. Qasrawi, S. P. V. Polo, D. Abu Al-Halawa, S. Hallaq and Z. Abdeen [45]	+	+	+	+	+	+	+	+	+	-	+	+	+	+	?	+	?	+	?	+
R. M. D. S. Rajapaksha, F. Filbey, S. Biswas and P. Choudhary [46]	+	+	+	-	+	+	+	+	+	-	+	+	+	+	?	+	?	+	?	+

W. A. Rothenberg, A. Bizzego, G. Esposito, J. E. Lansford, S. M. Al-Hassan, D. Bacchini, et al. [47]	+	+	+	+	+	+	+	+	+	-	+	+	+	+	?	+	+	+	?	+
C. Su, R. Aseltine, R. Doshi, K. Chen, S. C. Rogers and F. Wang [48]	+	+	+	?	+	+	+	+	+	-	+	+	+	+	?	+	?	+	?	+
M. J. Weintraub, F. Posta, M. C. Ichinose, A. C. Arevian and D. J. Miklowitz [49]	+	+	+	-	+	+	+	+	+	-	+	+	+	+	?	+	?	+	?	+
O. Weller, L. Sagers, C. Hanson, M. Barnes, Q. Snell and E. Shannon Tass [50]	+	+	+	-	+	+	?	?	+	?	+	+	+	+	?	+	+	+	?	+
Y. Zhang-James, Q. Chen, R. Kuja-Halkola, P. Lichtenstein, H. Larsson and S. V. Faraone [51]	+	+	+	+	+	+	+	+	+	?	+	+	+	+	+	+	+	+	+	+

Y. Zhong, J. He, J. Luo, J. Zhao, Y. Cen, Y. Song, et al. [52]	+	+	+	-	+	+	-	+	+	?	+	+	+	+	?	+	+	+	?	+
S. C. Zhou, Z. Zhou, Q. Tang, P. Yu, H. Zou, Q. Liu, et al. [53]	+	+	+	?	+	+	+	+	+	?	+	+	+	+	?	+	+	+	+	+

References

1. Khaleghi A, Sheikhani A, Mohammadi MR, Nasrabadi AM, Vand SR, Zarafshan H, Moeini M. EEG classification of adolescents with type I and type II of bipolar disorder. Australas Phys Eng Sci Med. 2015 Dec;38(4):551-9. doi: 10.1007/s13246-015-0375-0. PMID: 26472650.
2. Velupillai S, Epstein S, Bittar A, Stephenson T, Dutta R, Downs J. Identifying Suicidal Adolescents from Mental Health Records Using Natural Language Processing. Stud Health Technol Inform. 2019 Aug 21;264:413-417. doi: 10.3233/SHTI190254. PMID: 31437956.
3. Jin, L., Xue, Y., Li, Q., Feng, L. (2016). Integrating Human Mobility and Social Media for Adolescent Psychological Stress Detection. In: Navathe, S., Wu, W., Shekhar, S., Du, X., Wang, S., Xiong, H. (eds) Database Systems for Advanced Applications. DASFAA 2016. Lecture Notes in Computer Science(), vol 9643. Springer, Cham. https://doi.org/10.1007/978-3-319-32049-6_23
4. Y. -y. Gan.(2012)) Evaluation on life satisfaction of left-behind junior high school children based on LVQ network, 8th International Conference on Natural Computation, Chongqing, China, 2012, pp. 405-408, doi: 10.1109/ICNC.2012.6234755.
5. S. V. Tyulyupo, A. A. Andrakhanov, B. A. Dashieva and A. V. Tyryshkin, "Adolescents Psychological Well-Being Estimation Based on a Data Mining Algorithm," 2018 IEEE 13th International Scientific and Technical Conference on Computer Sciences and Information Technologies (CSIT), Lviv, Ukraine, 2018, pp. 475-478, doi: 10.1109/STC-CSIT.2018.8526628.
6. Xue, Y., Li, Q., Jin, L., Feng, L., Clifton, D.A., Clifford, G.D. (2014). Detecting Adolescent Psychological Pressures from Micro-Blog. In: Zhang, Y., Yao, G., He, J., Wang, L., Smalheiser, N.R., Yin, X. (eds) Health Information Science. HIS 2014. Lecture Notes in Computer Science, vol 8423. Springer, Cham. https://doi.org/10.1007/978-3-319-06269-3_10
7. Strigo IA, Murray SB, Simmons AN, Bernard RS, Huang JS, Kaye WH. The clinical application of fMRI data in a single-patient diagnostic conundrum: Classifying brain response to experimental pain to distinguish between gastrointestinal, depressive and eating disorder symptoms. J Clin Neurosci. 2017 Nov;45:149-153. doi: 10.1016/j.jocn.2017.07.023. Epub 2017 Aug 16. PMID: 28823587.

8. Zhang Z, Liao M, Yao Z, Hu B, Xie Y, Zheng W, Hu T, Zhao Y, Yang F, Zhang Y, Su L, Li L, Gutknecht J, Majoe D. Frequency-Specific Functional Connectivity Density as an Effective Biomarker for Adolescent Generalized Anxiety Disorder. Front Hum Neurosci. 2017 Dec 5;11:549. doi: 10.3389/fnhum.2017.00549. PMID: 29259549; PMCID: PMC5723402.
9. Chen H, Duan X, Liu F, Lu F, Ma X, Zhang Y, Uddin LQ, Chen H. Multivariate classification of autism spectrum disorder using frequency-specific resting-state functional connectivity--A multi-center study. Prog Neuropsychopharmacol Biol Psychiatry. 2016 Jan 4;64:1-9. doi: 10.1016/j.pnpbp.2015.06.014. Epub 2015 Jul 4. PMID: 26148789.
10. Thakur, S. (2016). Identification of Chief Characteristics of Alcohol Consumption Traits in Schools Using Rough Set and Formal Concept Analysis.
11. Foland-Ross LC, Sacchet MD, Prasad G, Gilbert B, Thompson PM, Gotlib IH. Cortical thickness predicts the first onset of major depression in adolescence. Int J Dev Neurosci. 2015 Nov;46:125-31. doi: 10.1016/j.ijdevneu.2015.07.007. Epub 2015 Aug 24. PMID: 26315399; PMCID: PMC4604750.
12. Hart H, Chantiluke K, Cubillo AI, Smith AB, Simmons A, Brammer MJ, Marquand AF, Rubia K. Pattern classification of response inhibition in ADHD: toward the development of neurobiological markers for ADHD. Hum Brain Mapp. 2014 Jul;35(7):3083-94. doi: 10.1002/hbm.22386. Epub 2013 Oct 11. PMID: 24123508; PMCID: PMC4190683.
13. Zhou Y, Yu F, Duong T. Multiparametric MRI characterization and prediction in autism spectrum disorder using graph theory and machine learning. PLoS One. 2014 Jun 12;9(6):e90405. doi: 10.1371/journal.pone.0090405. PMID: 24922325; PMCID: PMC4055499.
14. Ang RP, Goh DH. Predicting juvenile offending: a comparison of data mining methods. Int J Offender Ther Comp Criminol. 2013 Feb;57(2):191-207. doi: 10.1177/0306624X11431132. Epub 2011 Dec 12. PMID: 22158911.
15. Gervilla E, Cajal B, Palmer A. Quantification of the influence of friends and antisocial behaviour in adolescent consumption of cannabis using the ZINB model and data mining. Addict Behav. 2011 Apr;36(4):368-74. doi: 10.1016/j.addbeh.2010.12.007. Epub 2010 Dec 10. PMID: 21190799.
16. Kashani JH, Nair SS, Rao VG, Nair J, Reid JC. Relationship of personality, environmental, and DICA variables to adolescent hopelessness: a neural network sensitivity approach. J Am Acad Child Adolesc Psychiatry. 1996 May;35(5):640-5. doi: 10.1097/00004583-199605000-00019. PMID: 8935211.
17. Reid JC, Nair SS, Kashani JH, Rao VG. Detecting dysfunctional behavior in adolescents: the examination of relationships using neural networks. Proc Annu Symp Comput Appl Med Care. 1994:743-6. PMID: 7950023; PMCID: PMC2247956.
18. Y. Li, J. Huang, H. Wang and L. Feng, "Predicting Teenager's Future Stress Level from Micro-Blog," 2015 IEEE 28th International Symposium on Computer-Based Medical Systems, Sao Carlos, Brazil, 2015, pp. 208-213, doi: 10.1109/CBMS.2015.25.
19. DiGuiseppi GT, Davis JP, Leightley D, Rice E. Predictors of Adolescents' First Episode of Homelessness Following Substance Use Treatment. J Adolesc Health. 2020 Apr;66(4):408-415. doi: 10.1016/j.jadohealth.2019.11.312. Epub 2020 Feb 10. PMID: 32057607.

20. Ruan H, Zhou Y, Luo Q, Robert GH, Desrivières S, Quinlan EB, Liu Z, Banaschewski T, Bokde ALW, Bromberg U, Büchel C, Flor H, Frouin V, Garavan H, Gowland P, Heinz A, Ittermann B, Martinot JL, Martinot MP, Nees F, Orfanos DP, Poustka L, Hohmann S, Fröhner JH, Smolka MN, Walter H, Whelan R, Li F, Schumann G, Feng J; IMAGEN Consortium. Adolescent binge drinking disrupts normal trajectories of brain functional organization and personality maturation. Neuroimage Clin. 2019;22:101804. doi: 10.1016/j.nicl.2019.101804. Epub 2019 Mar 31. PMID: 30991616; PMCID: PMC6451196.
21. Downs J, Dean H, Lechler S, Sears N, Patel R, Shetty H, Hotopf M, Ford T, Kyriakopoulos M, Diaz-Caneja CM, Arango C, MacCabe JH, Hayes RD, Pina-Camacho L. Negative Symptoms in Early-Onset Psychosis and Their Association With Antipsychotic Treatment Failure. Schizophr Bull. 2019 Jan 1;45(1):69-79. doi: 10.1093/schbul/sbx197. PMID: 29370404; PMCID: PMC6293208.
22. Fitzgerald, A., Mac Giollabhui, N., Dolphin, L., Whelan, R., & Dooley, B. (2018). Dissociable psychosocial profiles of adolescent substance users. PLOS ONE, 13(8), e0202498. https://doi.org/10.1371/journal.pone.0202498
23. Fujisawa TX, Shimada K, Takiguchi S, Mizushima S, Kosaka H, Teicher MH, Tomoda A. Type and timing of childhood maltreatment and reduced visual cortex volume in children and adolescents with reactive attachment disorder. Neuroimage Clin. 2018 Jul 23;20:216-221. doi: 10.1016/j.nicl.2018.07.018. PMID: 30094171; PMCID: PMC6080635.
24. Barzman D, Ni Y, Griffey M, Bachtel A, Lin K, Jackson H, Sorter M, DelBello M. Automated Risk Assessment for School Violence: a Pilot Study. Psychiatr Q. 2018 Dec;89(4):817-828. doi: 10.1007/s11126-018-9581-8. PMID: 29713946.
25. Lenhard F, Sauer S, Andersson E, Månsson KN, Mataix-Cols D, Rück C, Serlachius E. Prediction of outcome in internet-delivered cognitive behaviour therapy for paediatric obsessive-compulsive disorder: A machine learning approach. Int J Methods Psychiatr Res. 2018 Mar;27(1):e1576. doi: 10.1002/mpr.1576. Epub 2017 Jul 28. PMID: 28752937; PMCID: PMC6877165.
26. Geraci J, Wilansky P, de Luca V, Roy A, Kennedy JL, Strauss J. Applying deep neural networks to unstructured text notes in electronic medical records for phenotyping youth depression. Evid Based Ment Health. 2017 Aug;20(3):83-87. doi: 10.1136/eb-2017-102688. Epub 2017 Jul 24. PMID: 28739578; PMCID: PMC5566092.
27. Squeglia LM, Ball TM, Jacobus J, Brumback T, McKenna BS, Nguyen-Louie TT, Sorg SF, Paulus MP, Tapert SF. Neural Predictors of Initiating Alcohol Use During Adolescence. Am J Psychiatry. 2017 Feb 1;174(2):172-185. doi: 10.1176/appi.ajp.2016.15121587. Epub 2016 Aug 19. Erratum in: Am J Psychiatry. 2017 Jan 1;174(1):80. doi: 10.1176/appi.ajp.2016.1741correction. PMID: 27539487; PMCID: PMC5288131.
28. Almuqhim F, Saeed F. ASD-SAENet: A Sparse Autoencoder, and Deep-Neural Network Model for Detecting Autism Spectrum Disorder (ASD) Using fMRI Data. Front Comput Neurosci. 2021 Apr 8;15:654315. doi: 10.3389/fncom.2021.654315. PMID: 33897398; PMCID: PMC8060560.
29. Bellod, H. C., Ramón, V. B., Fernández, E. C., & Luján, J. F. G. (2021). Analysis of stress and academic-sports commitment through Self-organizing Artificial Neural Networks. Retos: nuevas tendencias en educación física, deporte y recreación, (42), 136-144.

30. Li L, Pan N, Zhang L, Lui S, Huang X, Xu X, Wang S, Lei D, Li L, Kemp GJ, Gong Q. Hippocampal subfield alterations in pediatric patients with post-traumatic stress disorder. Soc Cogn Affect Neurosci. 2021 Mar 5;16(3):334-344. doi: 10.1093/scan/nsaa162. PMID: 33315100; PMCID: PMC7943370.
31. D. Kim et al., "Machine Learning Classification of First-Onset Drug-Naive MDD Using Structural MRI," in IEEE Access, vol. 7, pp. 153977-153985, 2019, doi: 10.1109/ACCESS.2019.2949128.
32. Kim KW, Lim JS, Yang CM, Jang SH, Lee SY. Classification of Adolescent Psychiatric Patients at High Risk of Suicide Using the Personality Assessment Inventory by Machine Learning. Psychiatry Investig. 2021 Nov;18(11):1137-1143. doi: 10.30773/pi.2021.0191. Epub 2021 Nov 5. PMID: 34732031; PMCID: PMC8600215.
33. Kiss O, Alzueta E, Yuksel D, Pohl KM, de Zambotti M,Műller-Oehring EM, Prouty D, Durley I, Pelham WE 3rd, McCabe CJ, Gonzalez MR, Brown SA, Wade NE, Marshall AT, Sowell ER, Breslin FJ, Lisdahl KM, Dick AS, Sheth CS, McCandliss BD, Guillaume M, Van Rinsveld AM, Dowling GJ, Tapert SF, Baker FC. The Pandemic's Toll on Young Adolescents: Prevention and Intervention Targets to Preserve Their Mental Health. J Adolesc Health. 2022 Mar;70(3):387-395. doi: 10.1016/j.jadohealth.2021.11.023. Epub 2022 Jan 26. PMID: 35090817; PMCID: PMC8789404.
34. Beaudequin, D., Schwenn, P., McLoughlin, L.T. et al. A novel, complex systems approach to modelling risk of psychological distress in young adolescents. Sci Rep 11, 9428 (2021). https://doi.org/10.1038/s41598-021-88932-y
35. Bao J, Wan J, Li H, Sun F. Psychological pain and sociodemographic factors classified suicide attempt and non-suicidal self-injury in adolescents. Acta Psychol (Amst). 2024 Jun;246:104271. doi: 10.1016/j.actpsy.2024.104271. Epub 2024 Apr 16. PMID: 38631150.
36. Haag AC, Bonanno GA, Chen S, Herd T, Strong-Jones S, S S, Noll JG. Understanding posttraumatic stress trajectories in adolescent females: A strength-based machine learning approach examining risk and protective factors including online behaviors. Dev Psychopathol. 2023 Oct;35(4):1794-1807. doi: 10.1017/S0954579422000475. Epub 2022 May 30. PMID: 35635211; PMCID: PMC9708933.
37. Bae SM, Lee SA, Lee SH. Prediction by data mining, of suicide attempts in Korean adolescents: a national study. Neuropsychiatr Dis Treat. 2015 Sep 16;11:2367-75. doi: 10.2147/NDT.S91111. PMID: 26396521; PMCID: PMC4577255.
38. Kaiser RH, Moser AD, Neilson C, Jones J, Peterson EC, Ruzic L, Rosenberg BM, Hough CM, Sandman C, Schneck CD, Miklowitz DJ. Neurocognitive risk phenotyping to predict mood symptoms in adolescence. J Psychopathol Clin Sci. 2024 Jan;133(1):90-102. doi: 10.1037/abn0000866. Epub 2023 Dec 7. PMID: 38059934; PMCID: PMC10752243.
39. Cilar Budler, L., Stiglic, G. Age, quality of life and mental well-being in adolescent population: a network model tree analysis. Sci Rep 13, 17667 (2023). https://doi.org/10.1038/s41598-023-44493-w
40. Bajaj, S., Blair, K.S., Dobbertin, M. et al. Machine learning based identification of structural brain alterations underlying suicide risk in adolescents. Discov Ment Health 3, 6 (2023). https://doi.org/10.1007/s44192-023-00033-6

41. J. B. Balano, V. L. Huerto, S. Sanchez, A. Saharkhiz and J. D. Goma, "Determining the Level of Depression using BDI-II through Voice Recognition," 2019 IEEE 6th International Conference on Industrial Engineering and Applications (ICIEA), Tokyo, Japan, 2019, pp. 387-391, doi: 10.1109/IEA.2019.8715187.
42. Barrios, J., Gabay, S., Cafiero, F., & Debbané, M. (2023, October 17). Detecting Psychological Disorders with Stylometry: the Case of ADHD in Adolescent Autobiographical Narratives. https://doi.org/10.31234/osf.io/s5cm3
43. Lim JS, Yang CM, Baek JW, Lee SY, Kim BN. Prediction Models for Suicide Attempts among Adolescents Using Machine Learning Techniques. Clin Psychopharmacol Neurosci. 2022 Nov 30;20(4):609-620. doi: 10.9758/cpn.2022.20.4.609. PMID: 36263637; PMCID: PMC9606439.
44. Penfold RB, Johnson E, Shortreed SM, Ziebell RA, Lynch FL, Clarke GN, Coleman KJ, Waitzfelder BE, Beck AL, Rossom RC, Ahmedani BK, Simon GE. Predicting suicide attempts and suicide deaths among adolescents following outpatient visits. J Affect Disord. 2021 Nov 1;294:39-47. doi: 10.1016/j.jad.2021.06.057. Epub 2021 Jul 1. PMID: 34265670; PMCID: PMC8820270.
45. Qasrawi R, Vicuna Polo SP, Abu Al-Halawa D, Hallaq S, Abdeen Z. Assessment and Prediction of Depression and Anxiety Risk Factors in Schoolchildren: Machine Learning Techniques Performance Analysis. JMIR Form Res. 2022 Aug 31;6(8):e32736. doi: 10.2196/32736. PMID: 35665695; PMCID: PMC9475423.
46. Rajapaksha RMDS, Filbey F, Biswas S, Choudhary P. A Bayesian learning model to predict the risk for cannabis use disorder. Drug Alcohol Depend. 2022 Jul 1;236:109476. doi: 10.1016/j.drugalcdep.2022.109476. Epub 2022 Apr 29. PMID: 35588608.
47. Rothenberg WA, Bizzego A, Esposito G, Lansford JE, Al-Hassan SM, Bacchini D, Bornstein MH, Chang L, Deater-Deckard K, Di Giunta L, Dodge KA, Gurdal S, Liu Q, Long Q, Oburu P, Pastorelli C, Skinner AT, Sorbring E, Tapanya S, Steinberg L, Tirado LMU, Yotanyamaneewong S, Alampay LP. Predicting Adolescent Mental Health Outcomes Across Cultures: A Machine Learning Approach. J Youth Adolesc. 2023 Aug;52(8):1595-1619. doi: 10.1007/s10964-023-01767-w. Epub 2023 Apr 19. PMID: 37074622; PMCID: PMC10113992.
48. Su, C., Aseltine, R., Doshi, R. et al. Machine learning for suicide risk prediction in children and adolescents with electronic health records. Transl Psychiatry 10, 413 (2020). https://doi.org/10.1038/s41398-020-01100-0
49. Weintraub MJ, Posta F, Ichinose MC, Arevian AC, Miklowitz DJ. Word usage in spontaneous speech as a predictor of depressive symptoms among youth at high risk for mood disorders. J Affect Disord. 2023 Feb 15;323:675-678. doi: 10.1016/j.jad.2022.12.047. Epub 2022 Dec 14. PMID: 36528134; PMCID: PMC9848879.
50. Weller O, Sagers L, Hanson C, Barnes M, Snell Q, Tass ES. Predicting suicidal thoughts and behavior among adolescents using the risk and protective factor framework: A large-scale machine learning approach. PLoS One. 2021 Nov 3;16(11):e0258535. doi: 10.1371/journal.pone.0258535. PMID: 34731169; PMCID: PMC8565727.
51. Zhang-James Y, Chen Q, Kuja-Halkola R, Lichtenstein P, Larsson H, Faraone SV. Machine-Learning prediction of comorbid substance use disorders in ADHD youth using Swedish registry data. J Child Psychol Psychiatry. 2020 Dec;61(12):1370-1379. doi: 10.1111/jcpp.13226. Epub 2020 Apr 1. PMID: 32237241; PMCID: PMC7754321.

52. Zhong Y, He J, Luo J, Zhao J, Cen Y, Song Y, Wu Y, Lin C, Pan L, Luo J. A machine learning algorithm-based model for predicting the risk of non-suicidal self-injury among adolescents in western China: A multicentre cross-sectional study. J Affect Disord. 2024 Jan 15;345:369-377. doi: 10.1016/j.jad.2023.10.110. Epub 2023 Oct 26. PMID: 37898476.
53. Zhou SC, Zhou Z, Tang Q, Yu P, Zou H, Liu Q, Wang XQ, Jiang J, Zhou Y, Liu L, Yang BX, Luo D. Prediction of non-suicidal self-injury in adolescents at the family level using regression methods and machine learning. J Affect Disord. 2024 May 1;352:67-75. doi: 10.1016/j.jad.2024.02.039. Epub 2024 Feb 13. PMID: 38360362.
